# Supplementary figures and images for: Pdk3’s role in RANKL-induced osteoclast differentiation: insights from a bone marrow macrophage model
Source: PeerJ. 2024 Oct 9;12:e18222. doi: 10.7717/peerj.18222 (PMC11470767; doi:10.7717/peerj.18222)

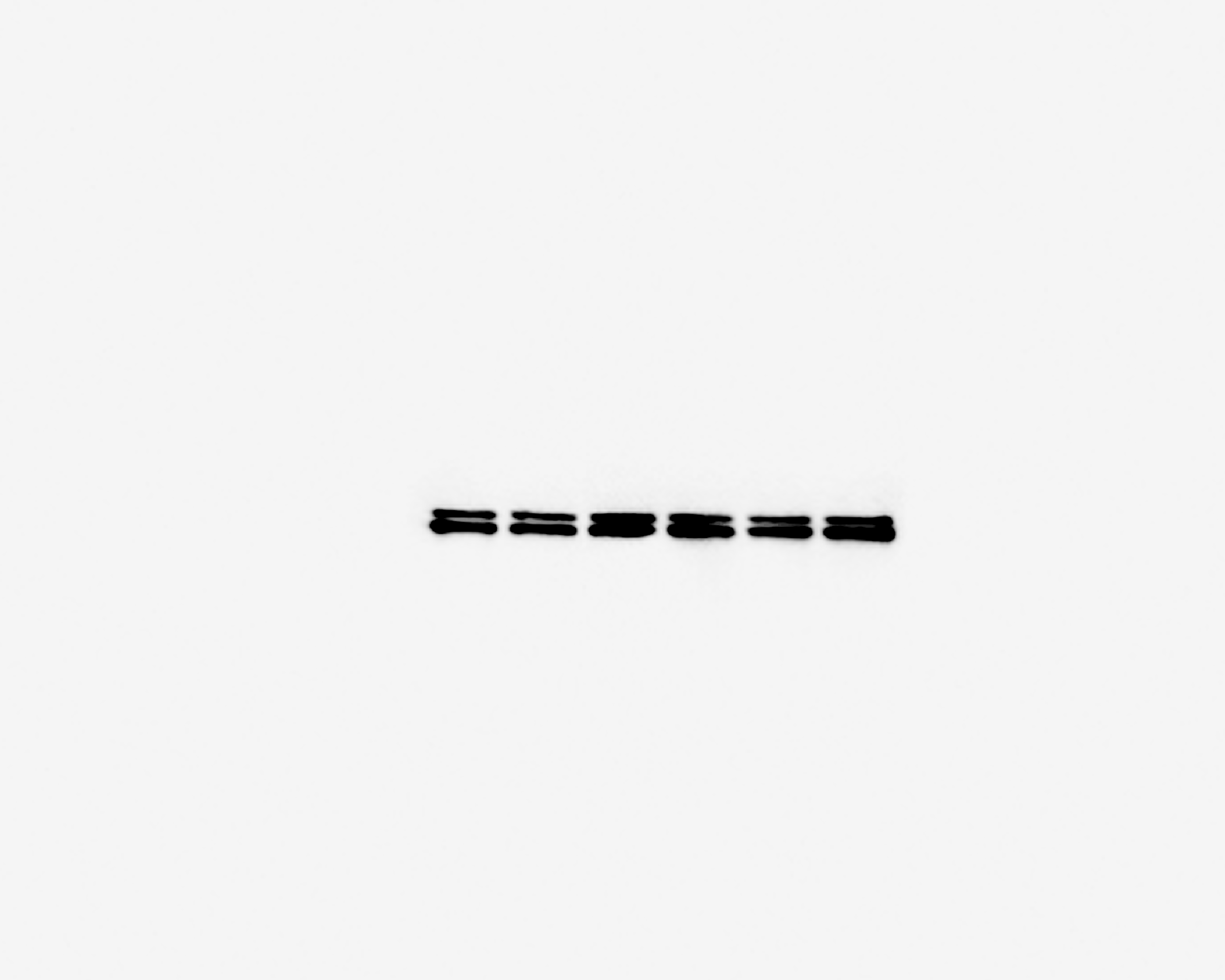

Supplement: Supplemental Information 2 [file peerj-12-18222-s002.zip › erk/perk+erk 1_1(Chemiluminescence).tif]

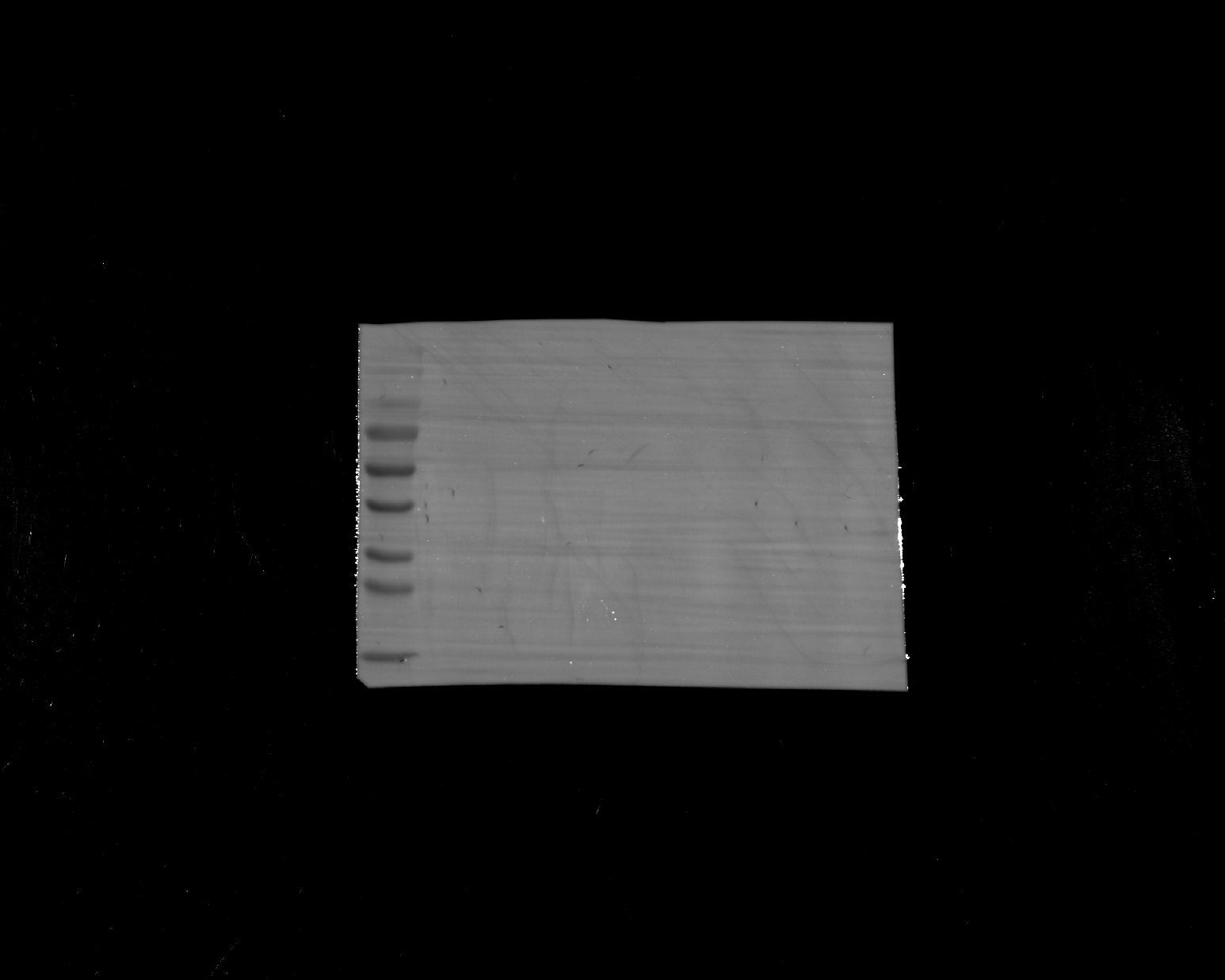

Supplement: Supplemental Information 2 [file peerj-12-18222-s002.zip › erk/perk+erk 1_1(Colorimetric).tif]

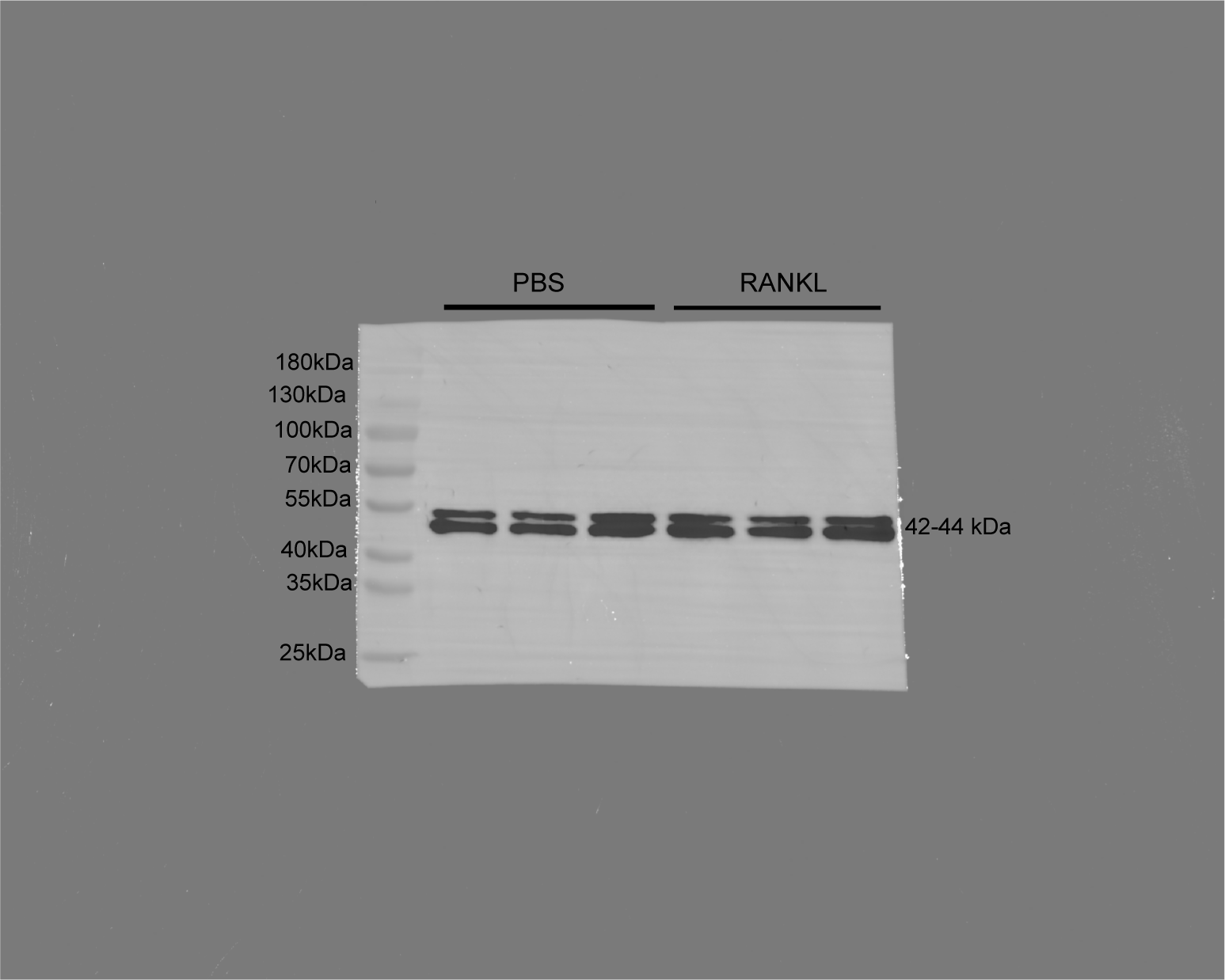

Supplement: Supplemental Information 2 [file peerj-12-18222-s002.zip › erk/perk+erk 1_1(Composite)-01.tif]

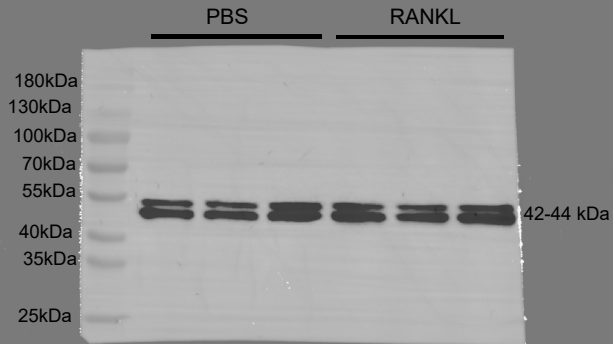

Supplement: Supplemental Information 2 [file peerj-12-18222-s002.zip › erk/perk+erk 1_1(Composite).pdf]

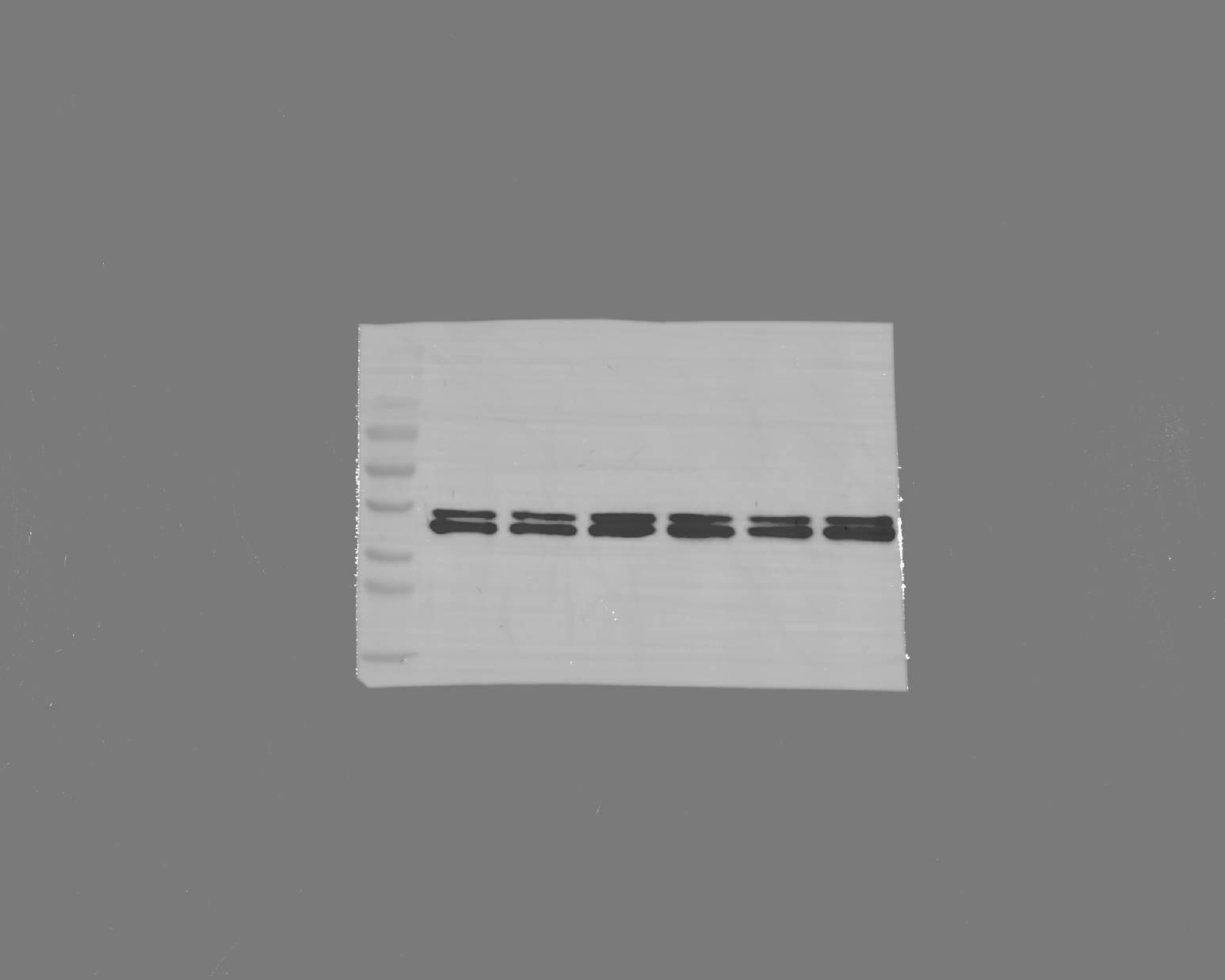

Supplement: Supplemental Information 2 [file peerj-12-18222-s002.zip › erk/perk+erk 1_1(Composite).tif]

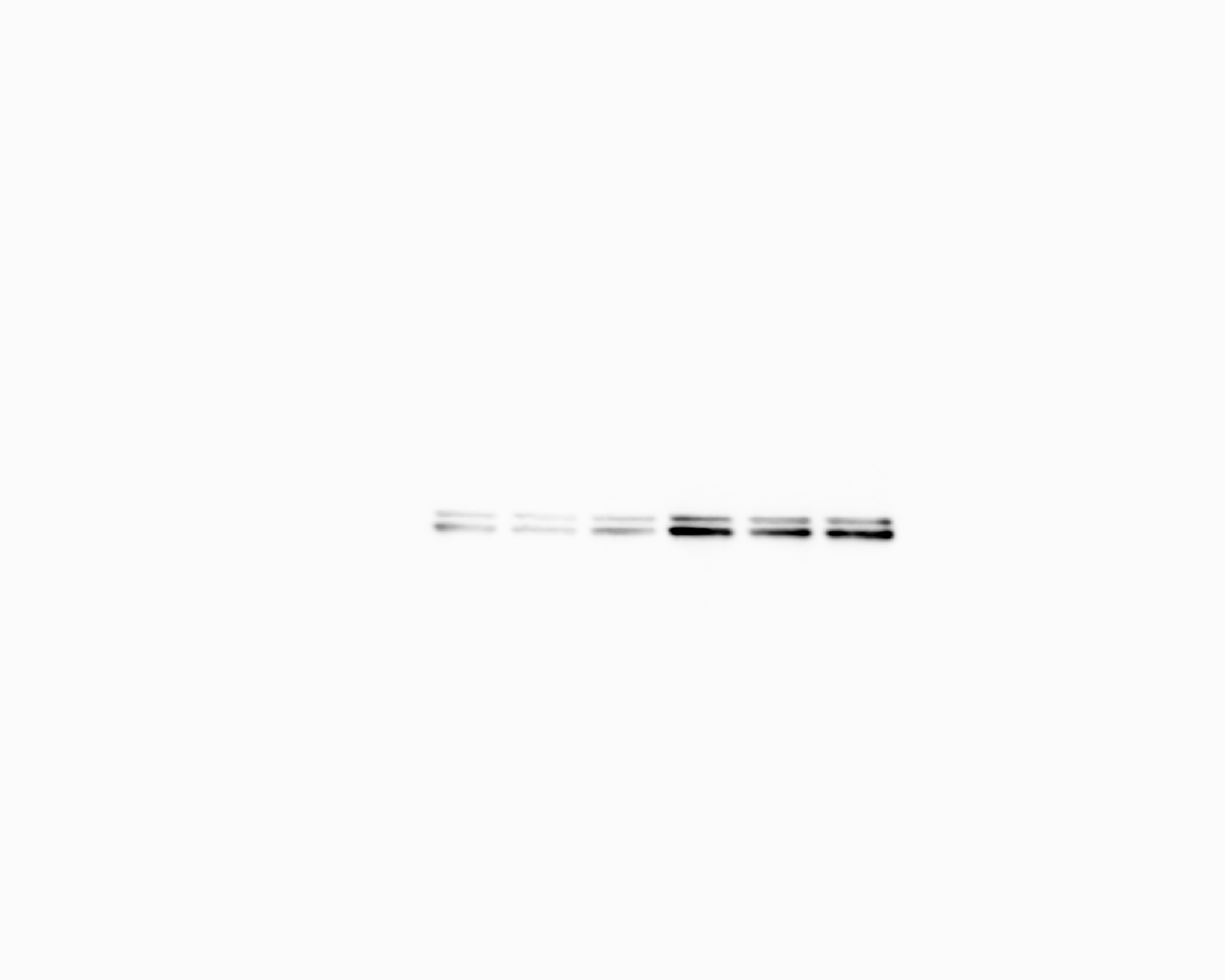

Supplement: Supplemental Information 2 [file peerj-12-18222-s002.zip › erk/perk+erk 1_2(Chemiluminescence).tif]

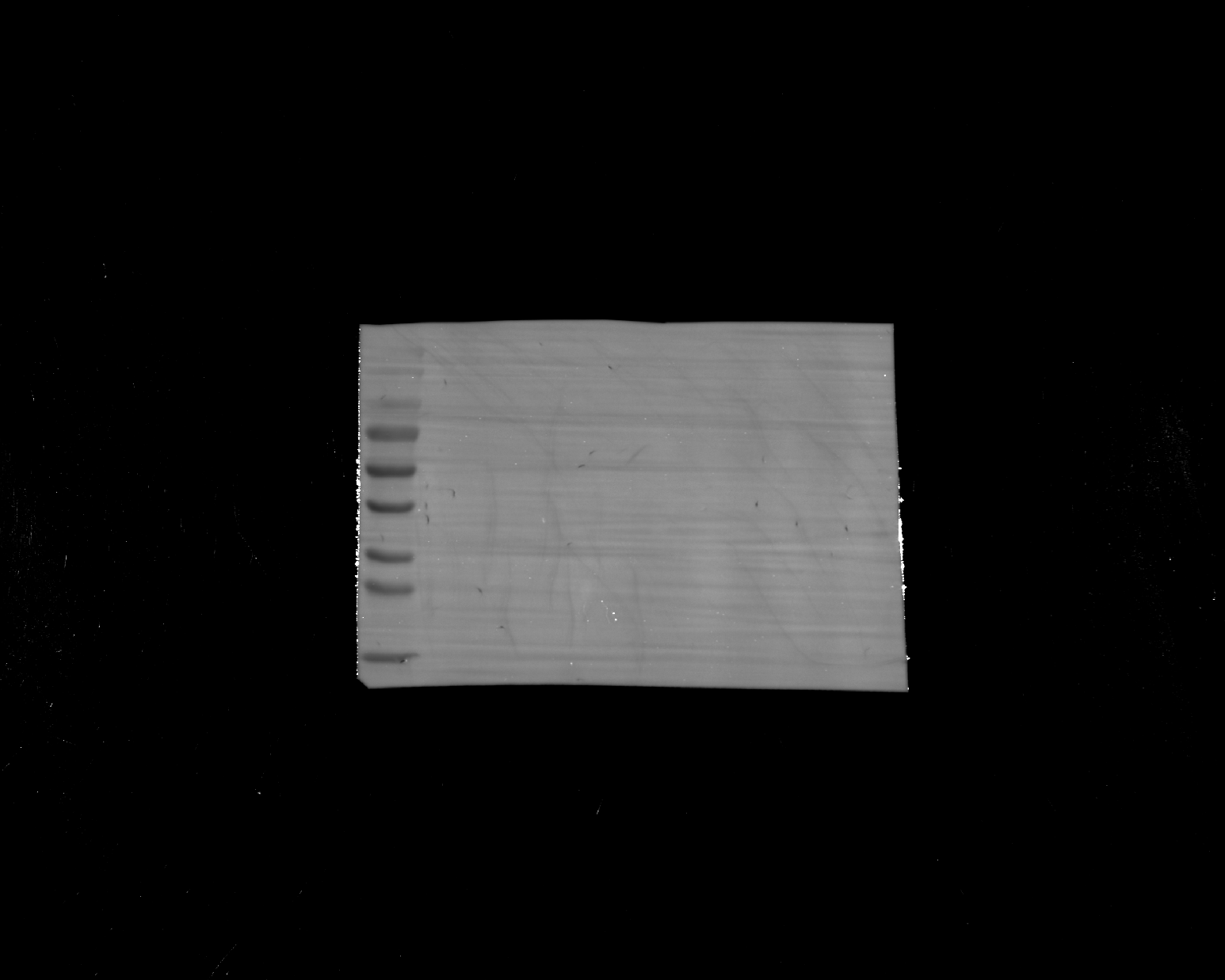

Supplement: Supplemental Information 2 [file peerj-12-18222-s002.zip › erk/perk+erk 1_2(Colorimetric).tif]

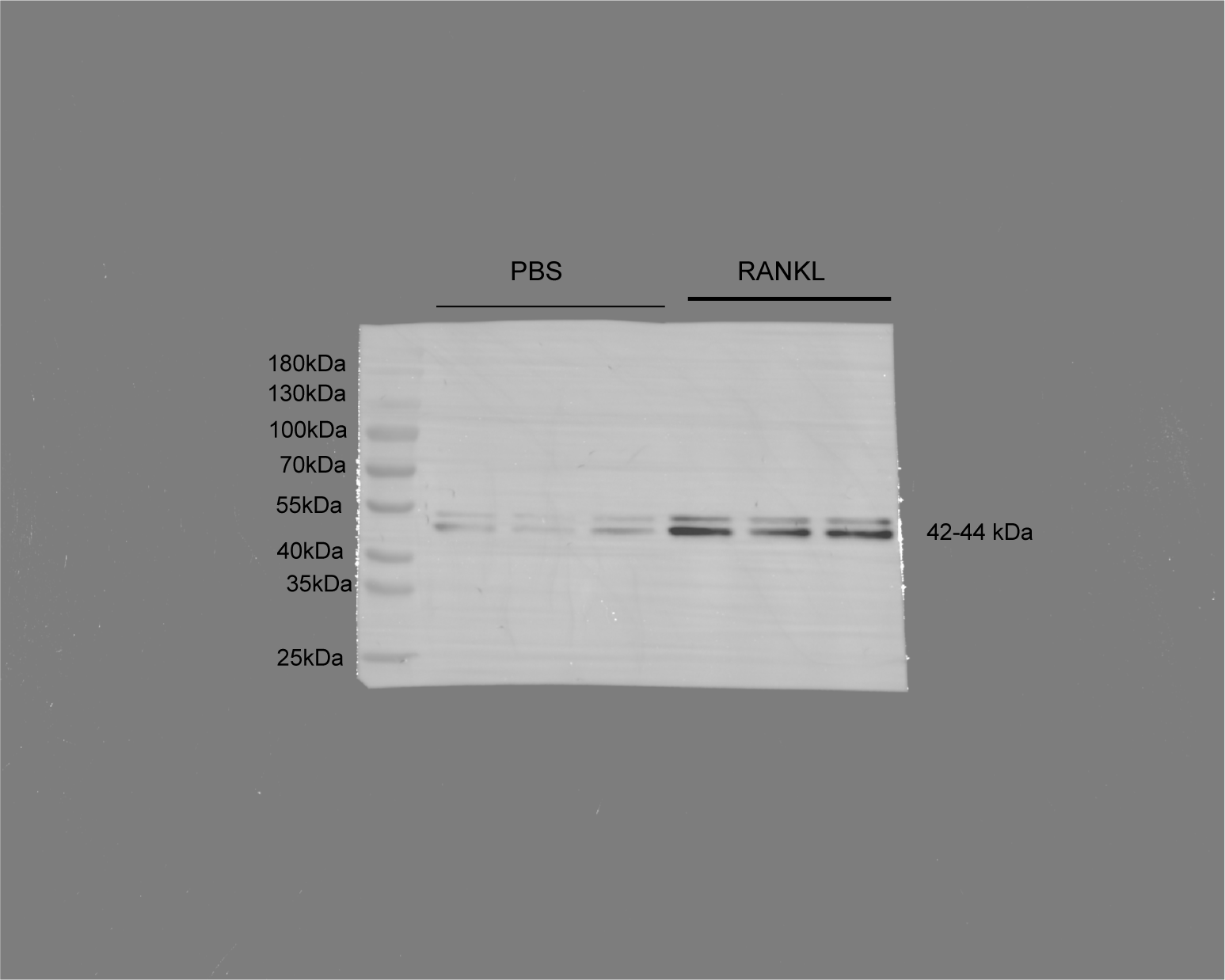

Supplement: Supplemental Information 2 [file peerj-12-18222-s002.zip › erk/perk+erk 1_2(Composite)-01.tif]

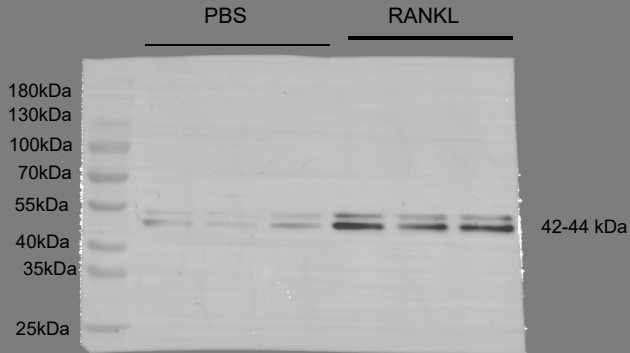

Supplement: Supplemental Information 2 [file peerj-12-18222-s002.zip › erk/perk+erk 1_2(Composite).pdf]

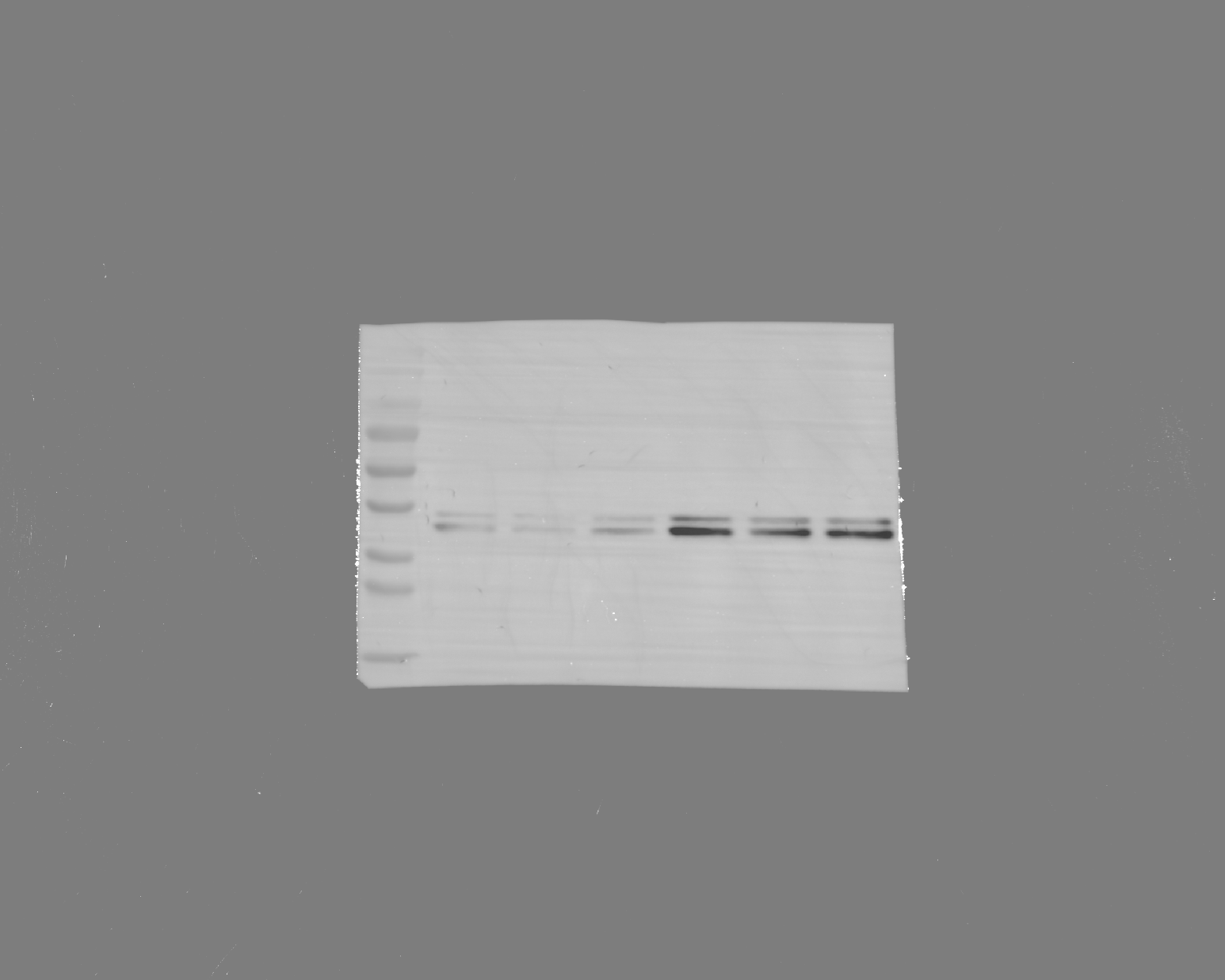

Supplement: Supplemental Information 2 [file peerj-12-18222-s002.zip › erk/perk+erk 1_2(Composite).tif]

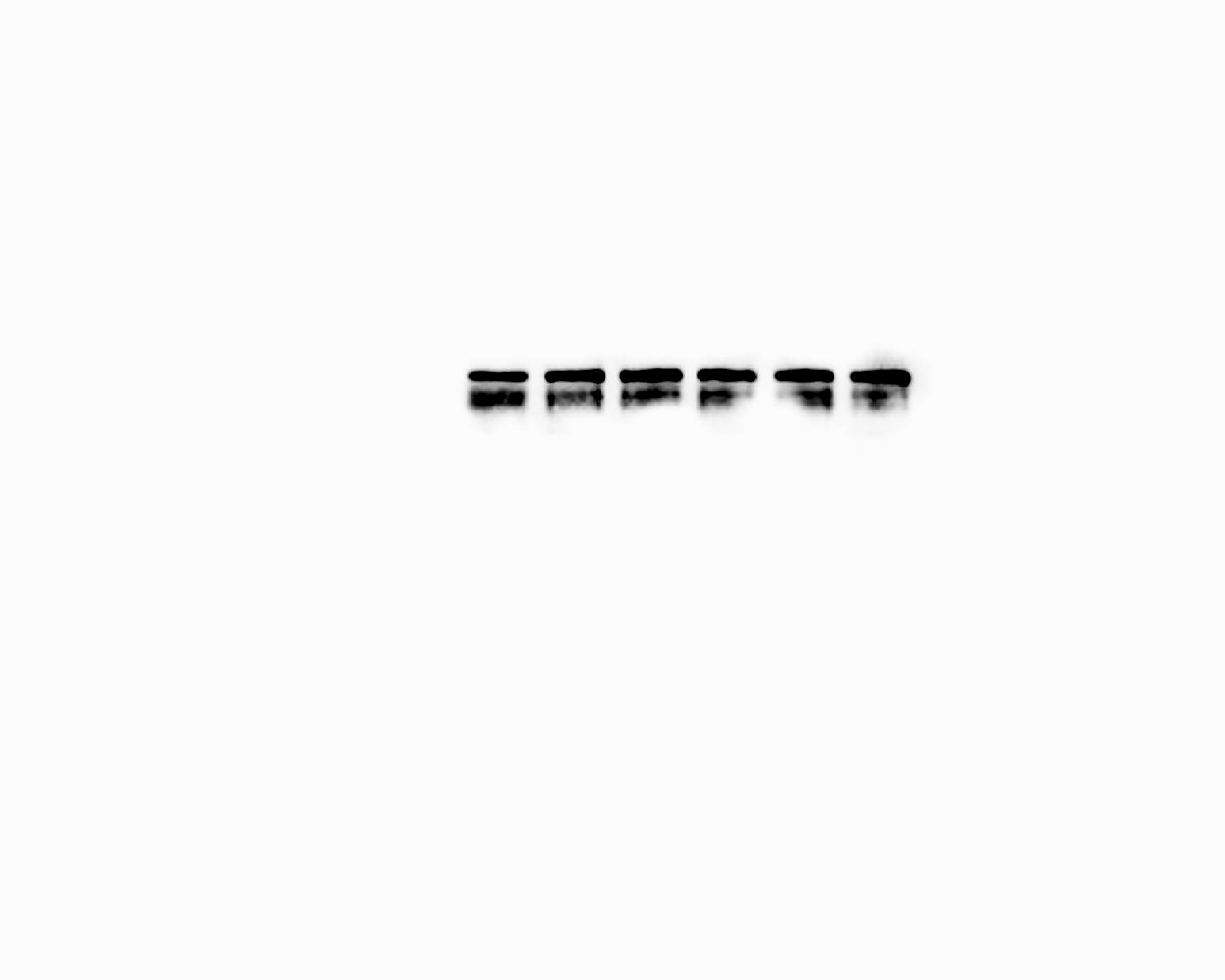

Supplement: Supplemental Information 2 [file peerj-12-18222-s002.zip › jak/pjak1+jak1 2_1(Chemiluminescence).tif]

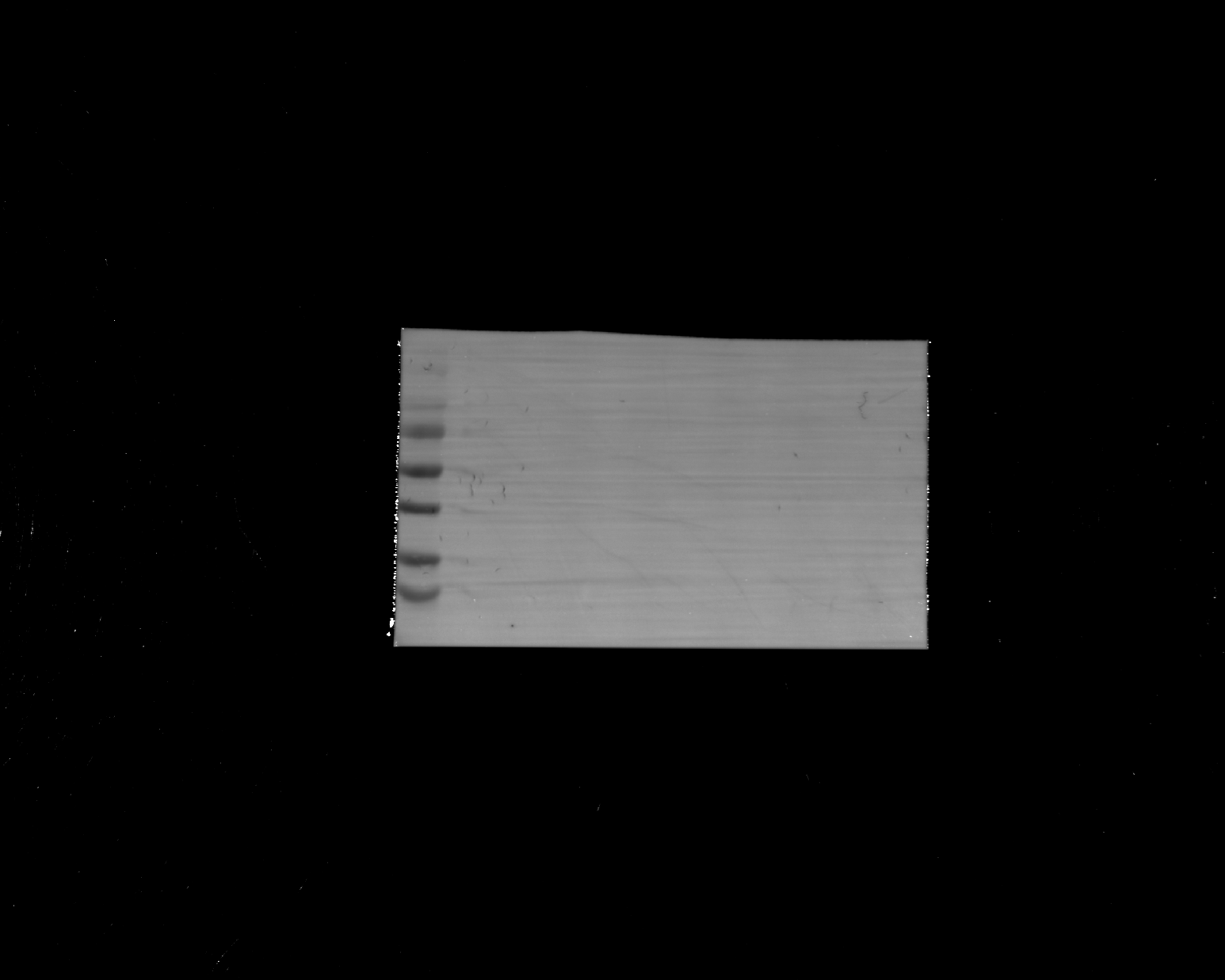

Supplement: Supplemental Information 2 [file peerj-12-18222-s002.zip › jak/pjak1+jak1 2_1(Colorimetric).tif]

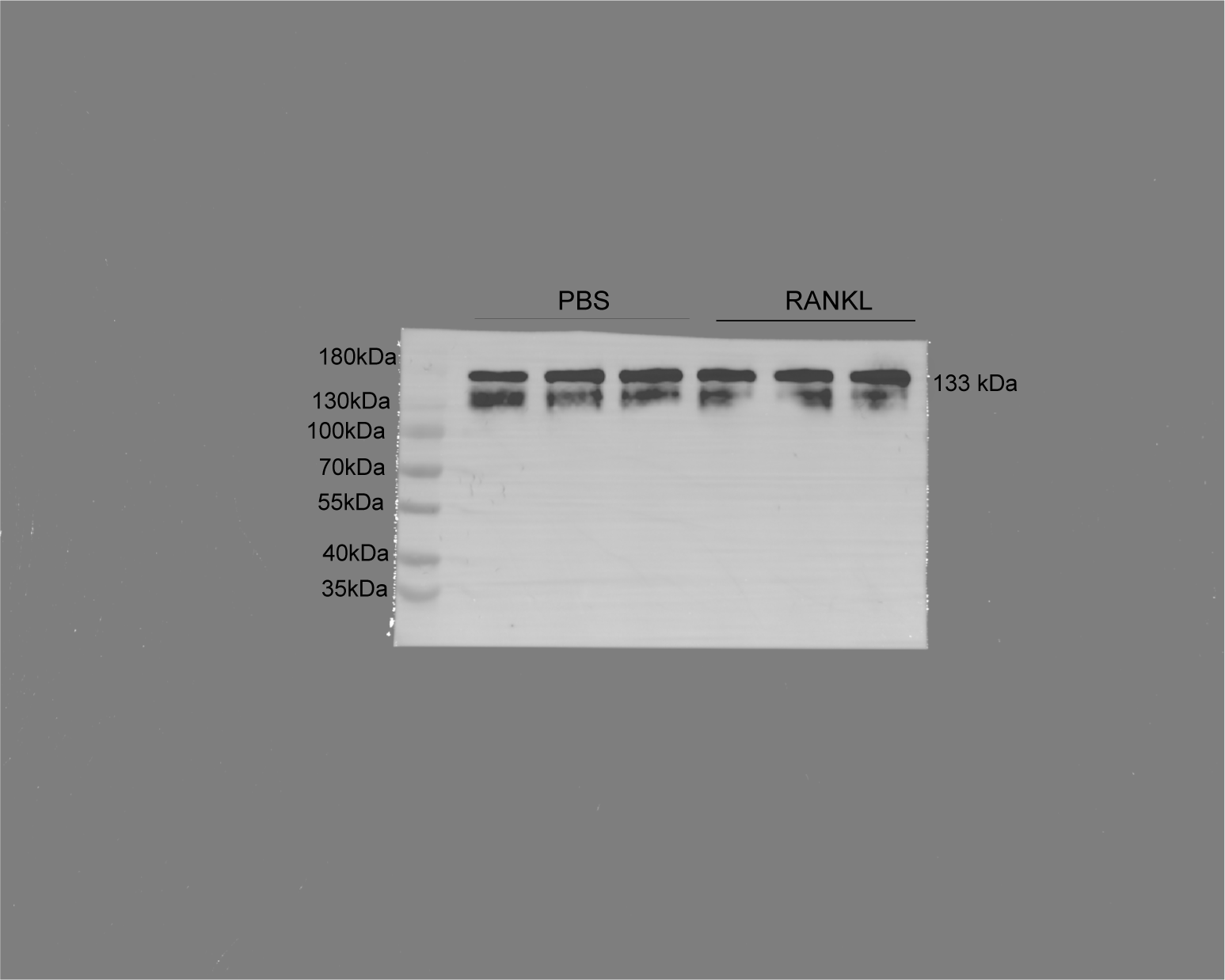

Supplement: Supplemental Information 2 [file peerj-12-18222-s002.zip › jak/pjak1+jak1 2_1(Composite)-01.tif]

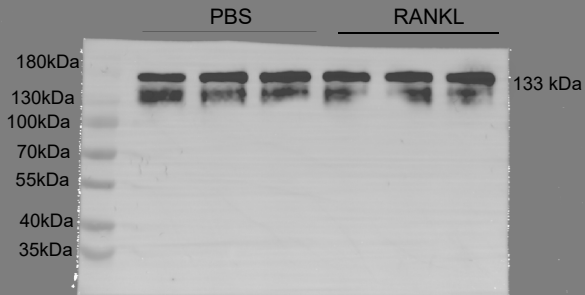

Supplement: Supplemental Information 2 [file peerj-12-18222-s002.zip › jak/pjak1+jak1 2_1(Composite).pdf]

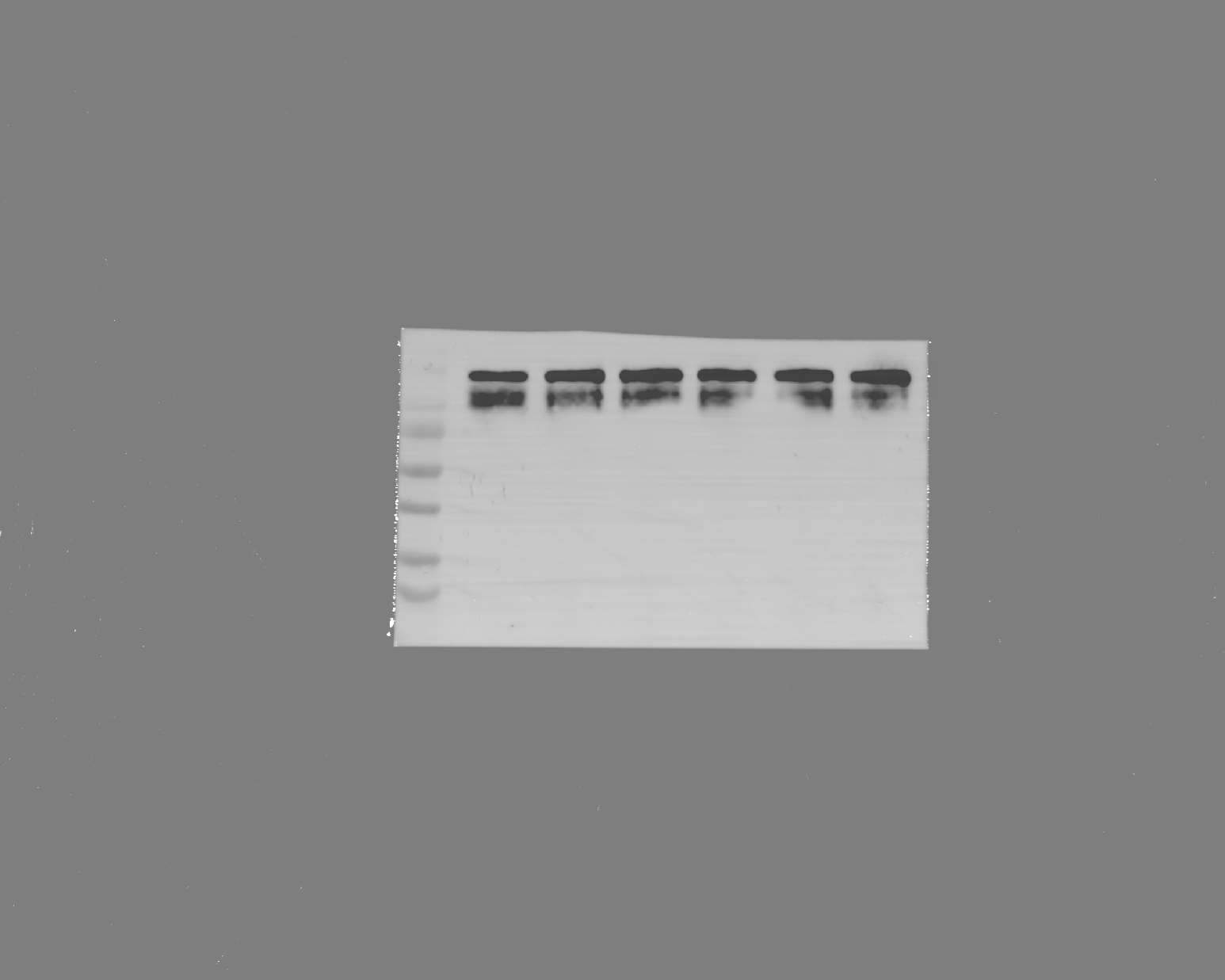

Supplement: Supplemental Information 2 [file peerj-12-18222-s002.zip › jak/pjak1+jak1 2_1(Composite).tif]

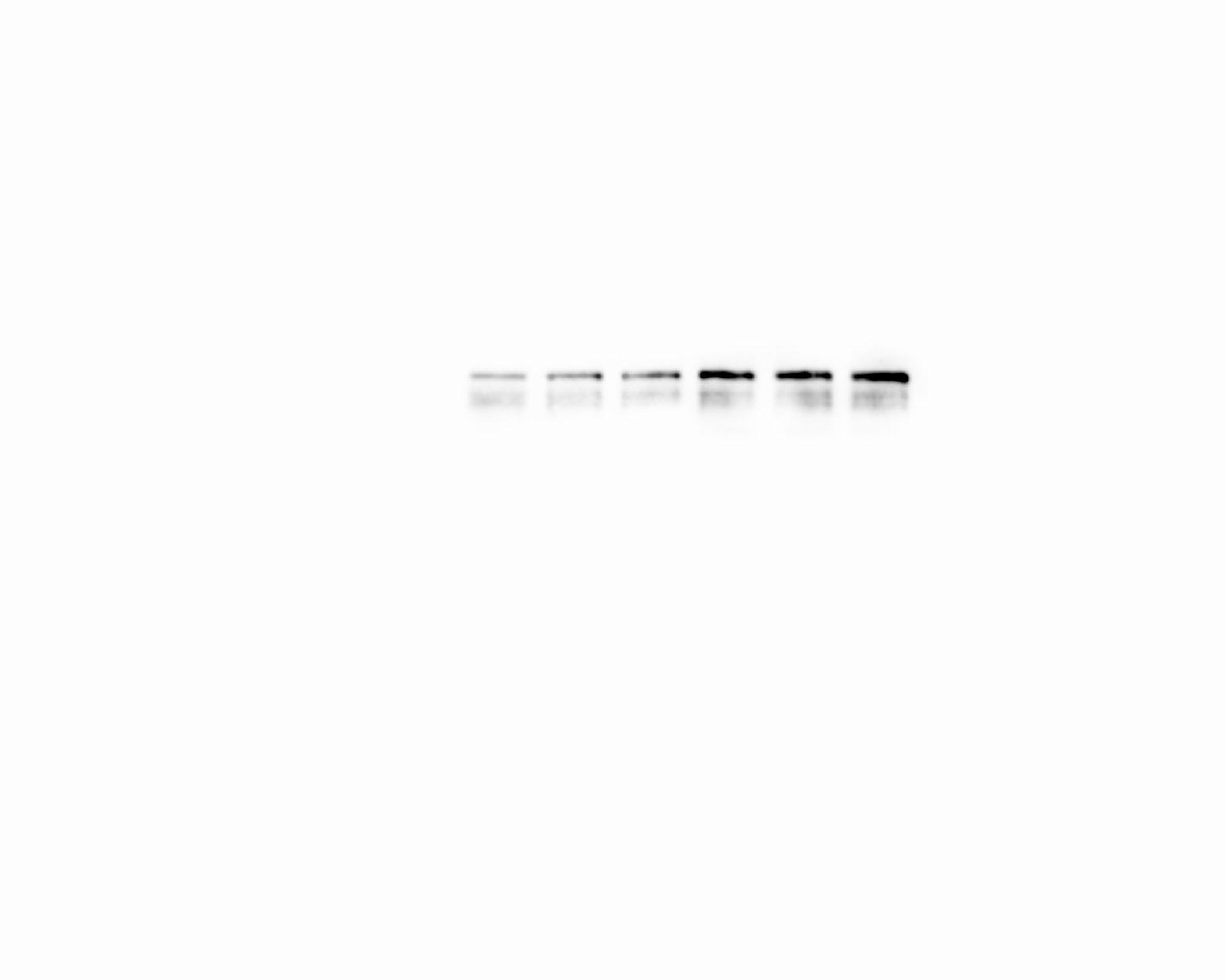

Supplement: Supplemental Information 2 [file peerj-12-18222-s002.zip › jak/pjak1+jak1 2_2(Chemiluminescence).tif]

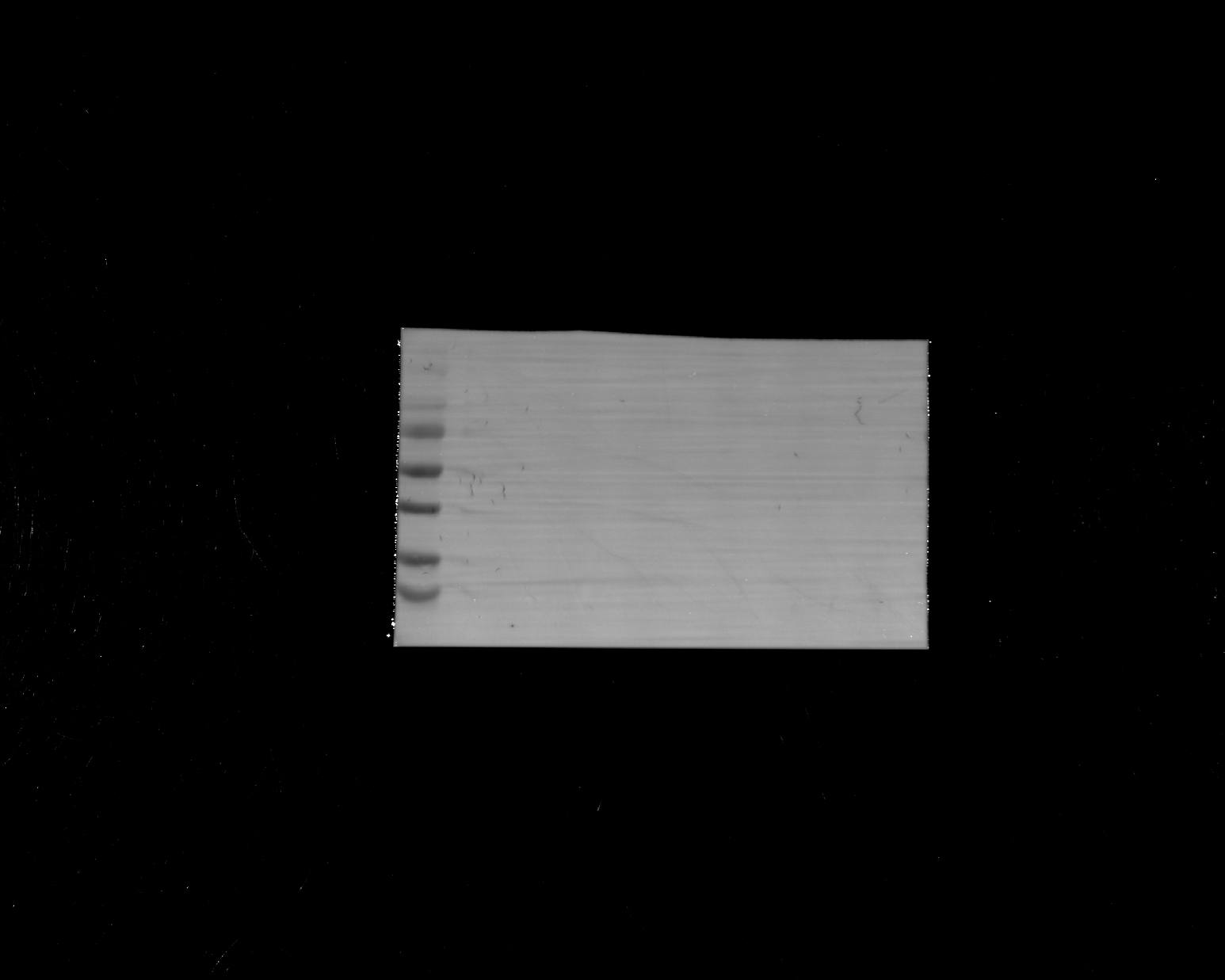

Supplement: Supplemental Information 2 [file peerj-12-18222-s002.zip › jak/pjak1+jak1 2_2(Colorimetric).tif]

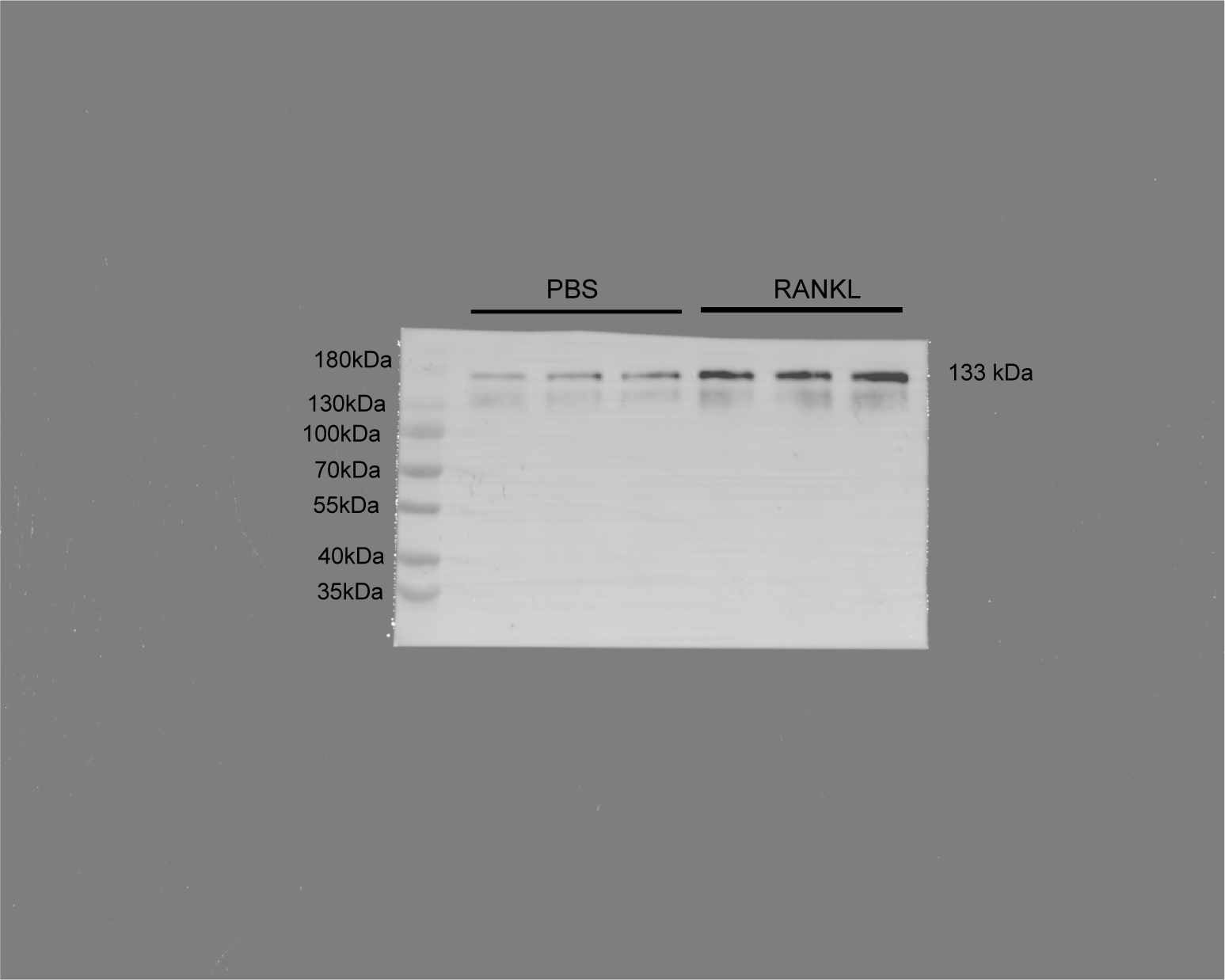

Supplement: Supplemental Information 2 [file peerj-12-18222-s002.zip › jak/pjak1+jak1 2_2(Composite)-01.tif]

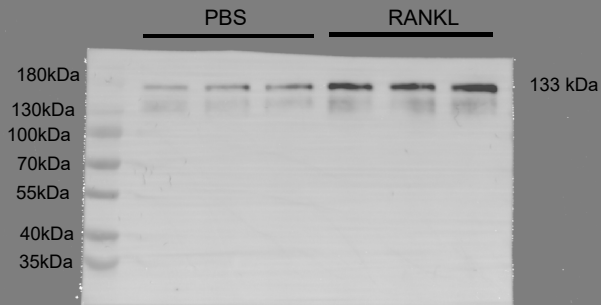

Supplement: Supplemental Information 2 [file peerj-12-18222-s002.zip › jak/pjak1+jak1 2_2(Composite).pdf]

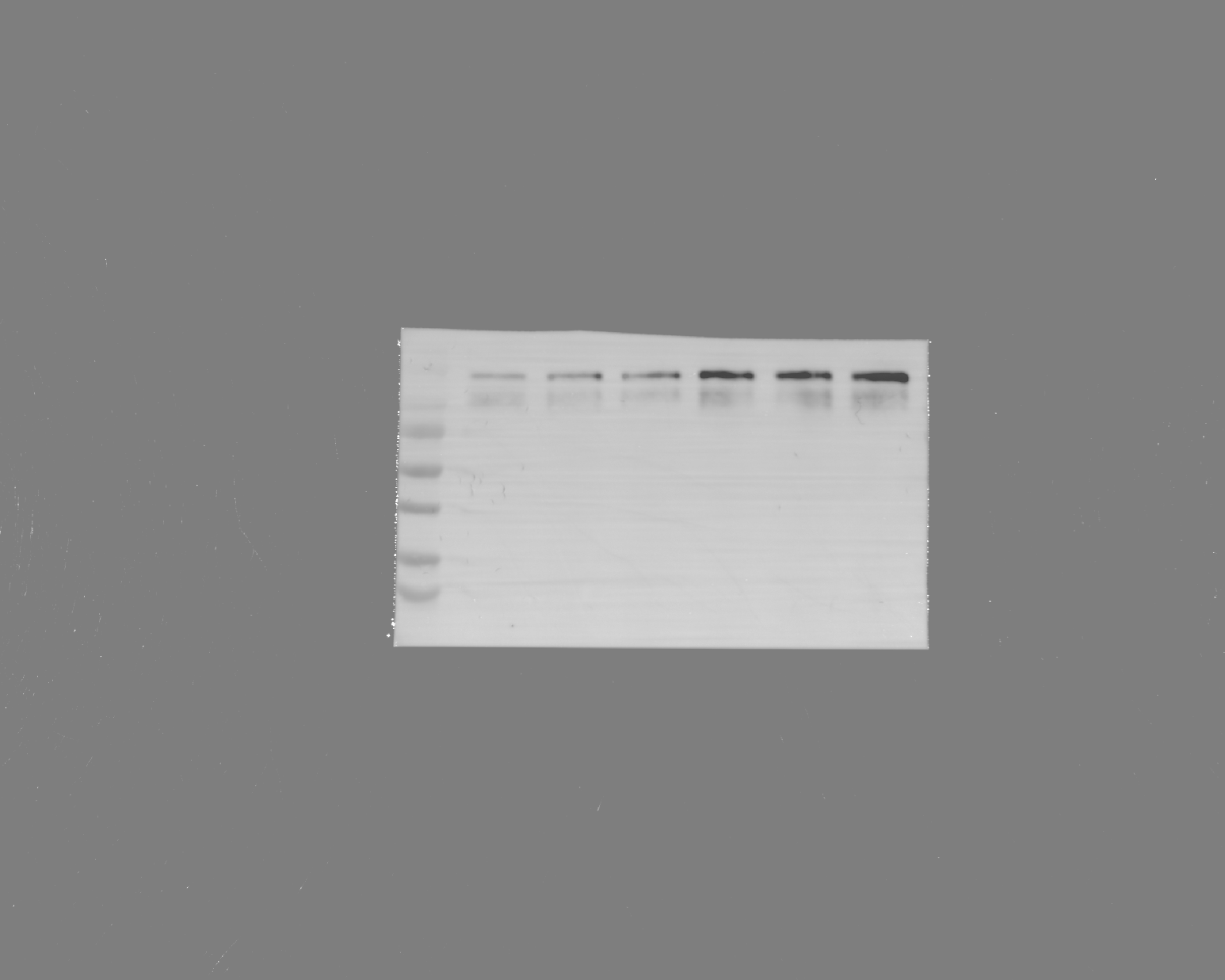

Supplement: Supplemental Information 2 [file peerj-12-18222-s002.zip › jak/pjak1+jak1 2_2(Composite).tif]

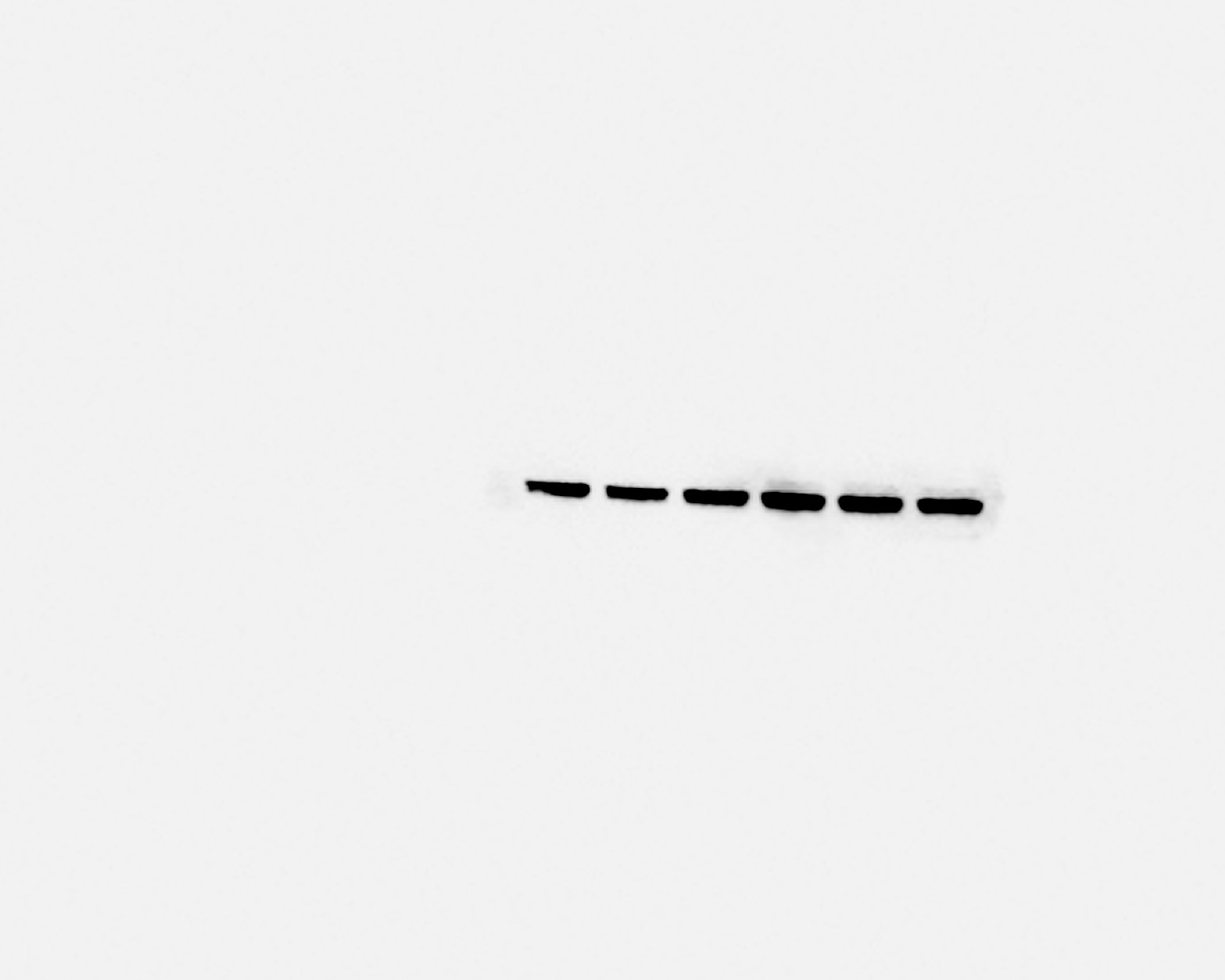

Supplement: Supplemental Information 2 [file peerj-12-18222-s002.zip › p65/6 pp65+p65 1_1(Chemiluminescence).tif]

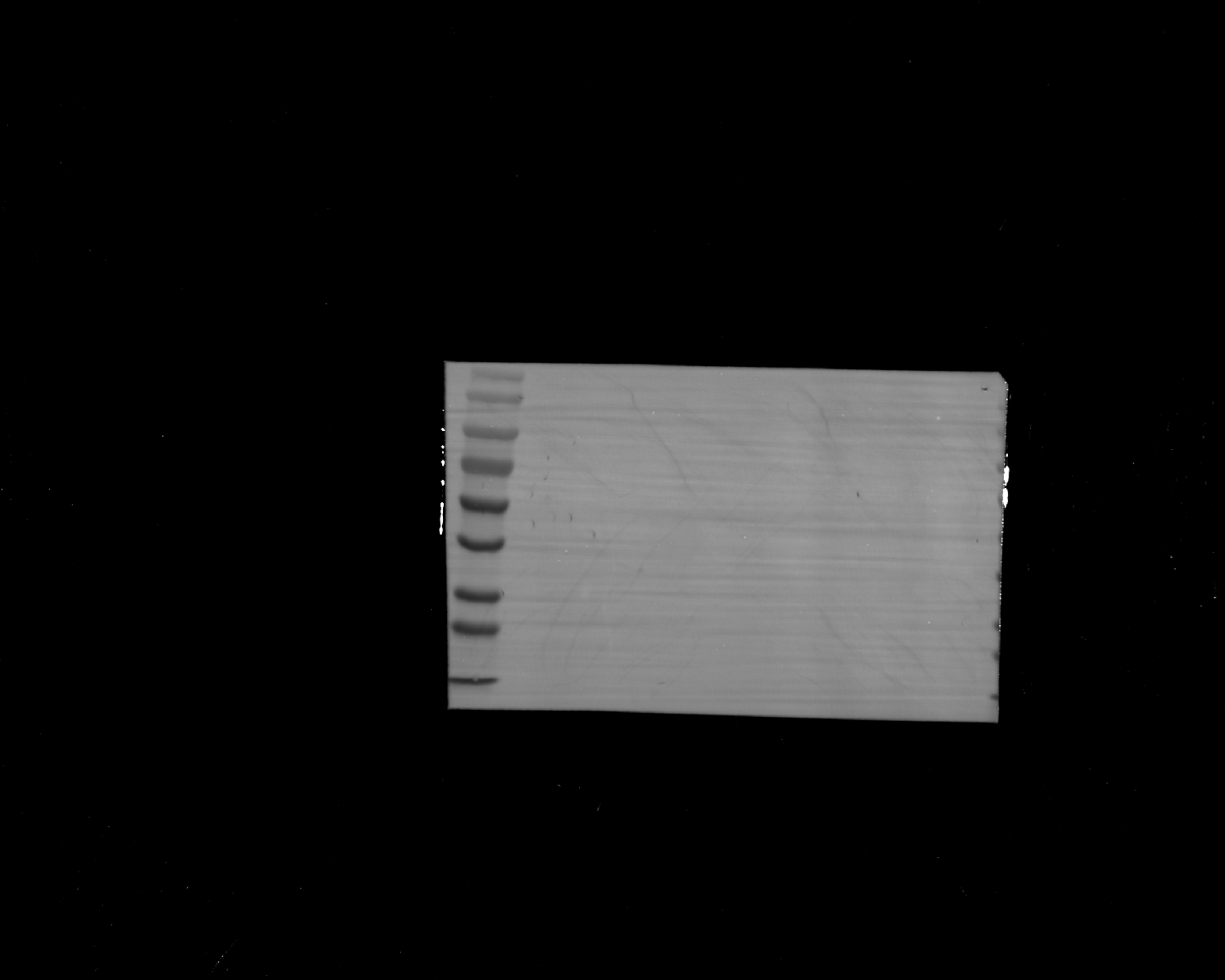

Supplement: Supplemental Information 2 [file peerj-12-18222-s002.zip › p65/6 pp65+p65 1_1(Colorimetric).tif]

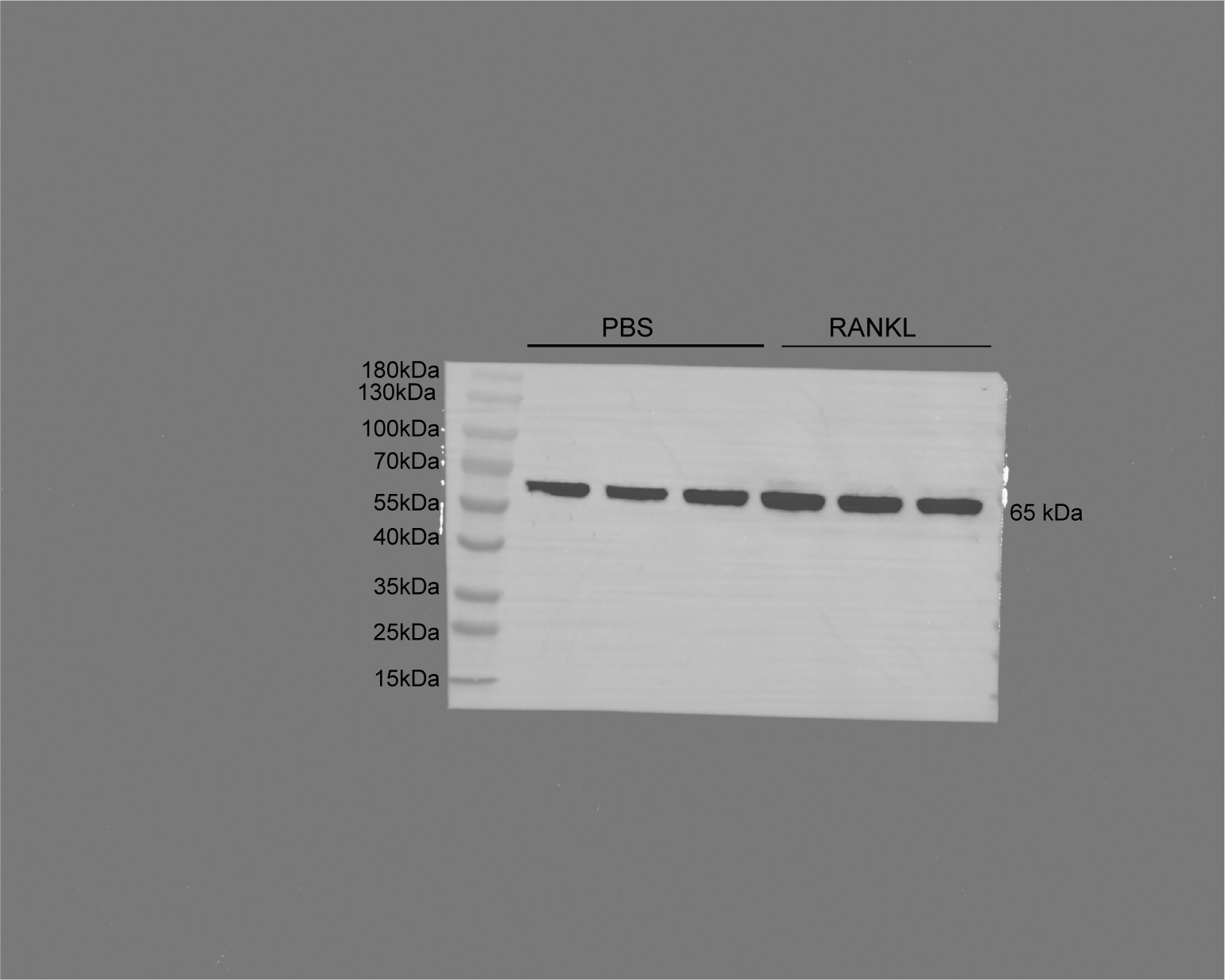

Supplement: Supplemental Information 2 [file peerj-12-18222-s002.zip › p65/6 pp65+p65 1_1(Composite)-01.tif]

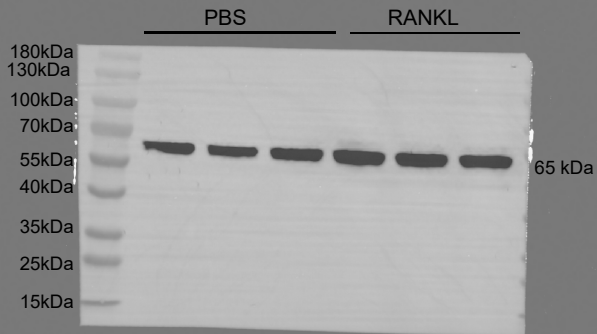

Supplement: Supplemental Information 2 [file peerj-12-18222-s002.zip › p65/6 pp65+p65 1_1(Composite).pdf]

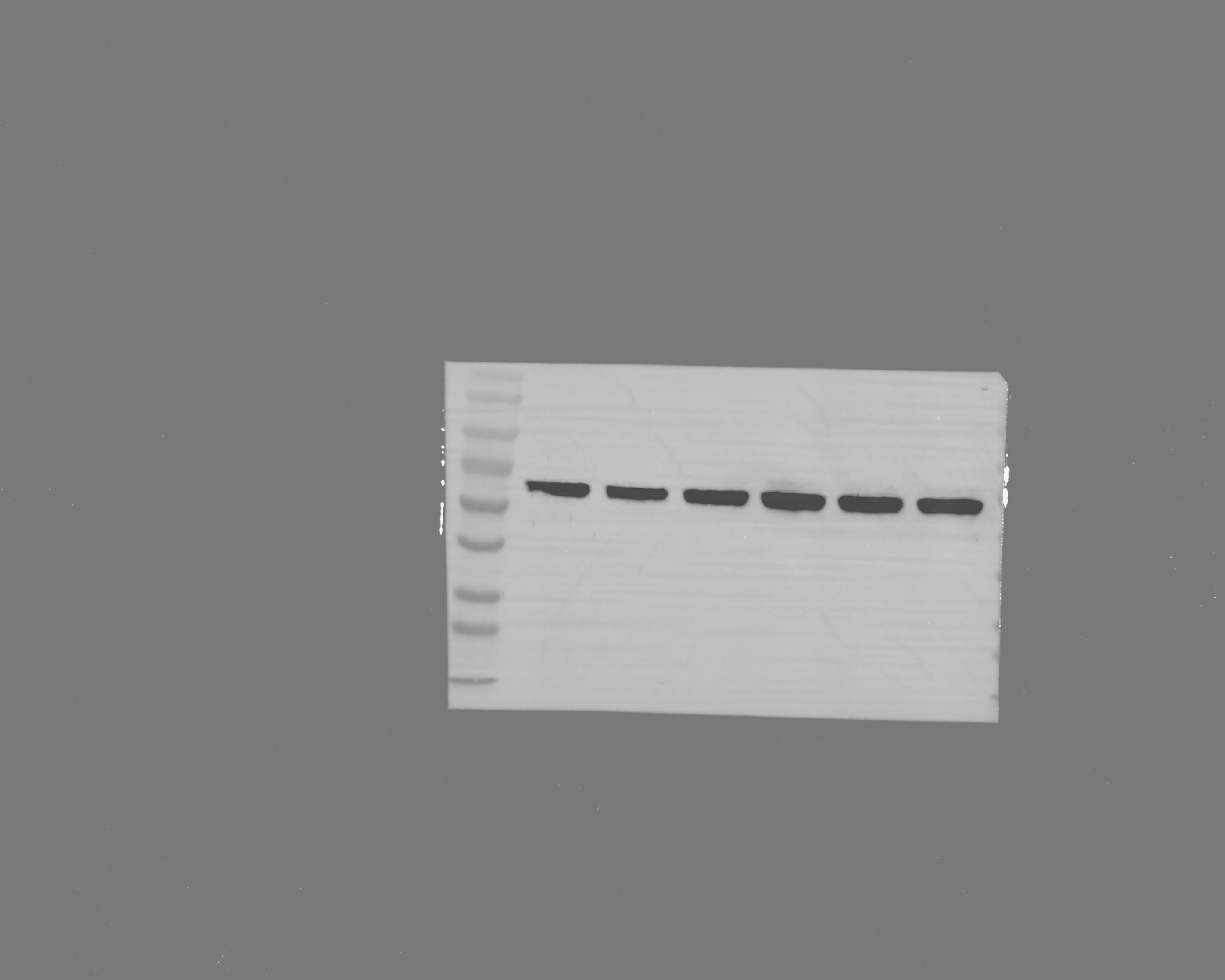

Supplement: Supplemental Information 2 [file peerj-12-18222-s002.zip › p65/6 pp65+p65 1_1(Composite).tif]

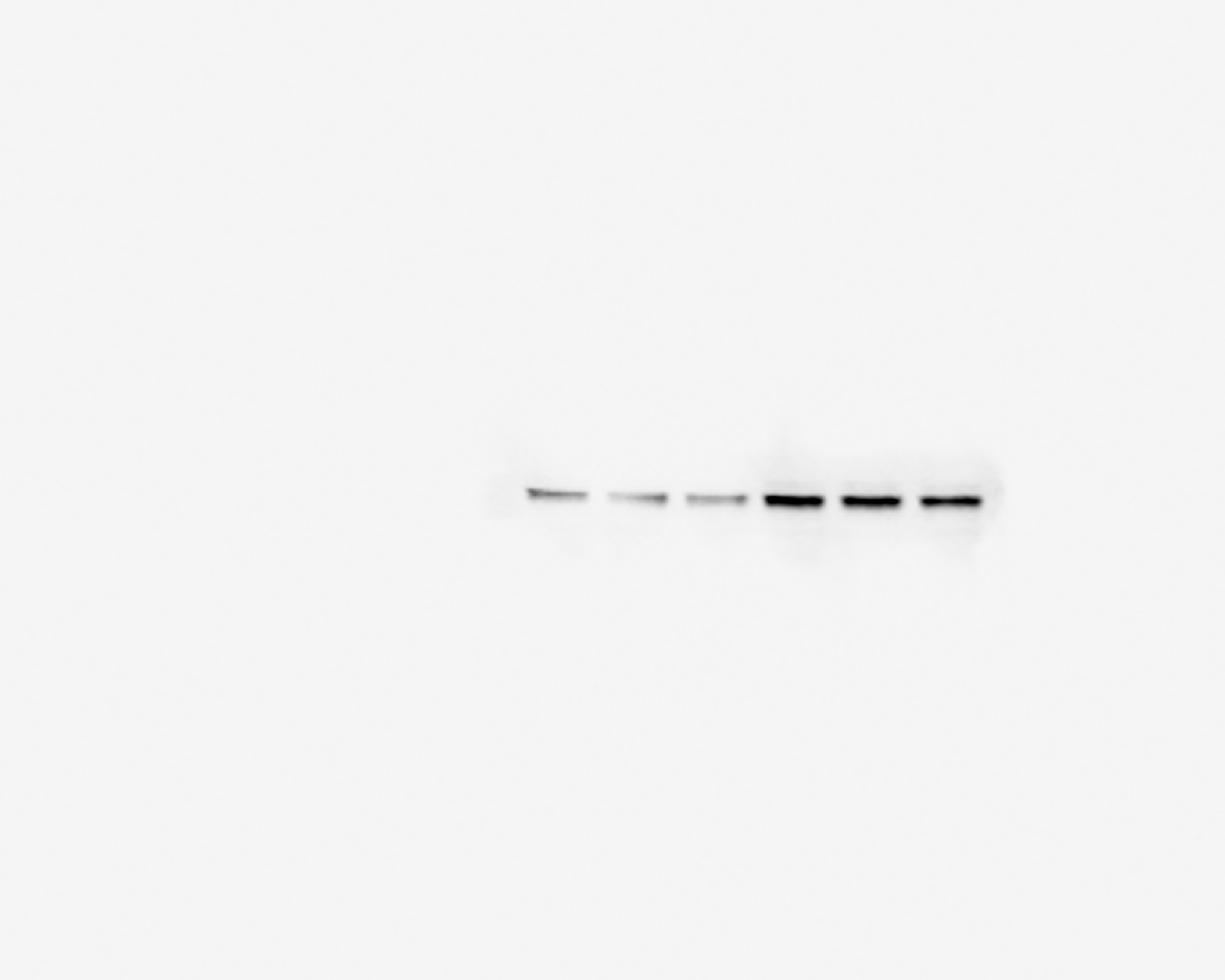

Supplement: Supplemental Information 2 [file peerj-12-18222-s002.zip › p65/6 pp65+p65 1_2(Chemiluminescence).tif]

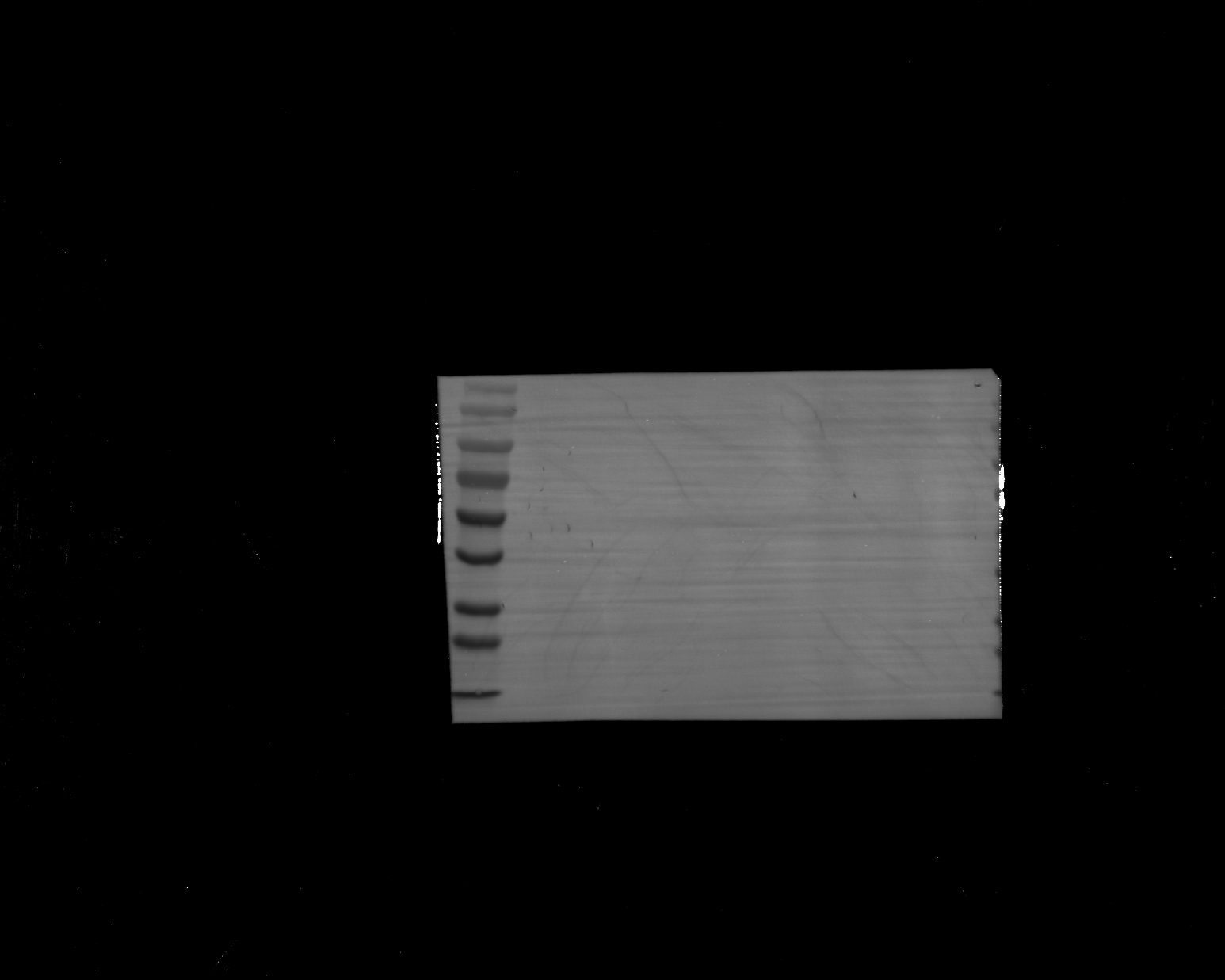

Supplement: Supplemental Information 2 [file peerj-12-18222-s002.zip › p65/6 pp65+p65 1_2(Colorimetric).tif]

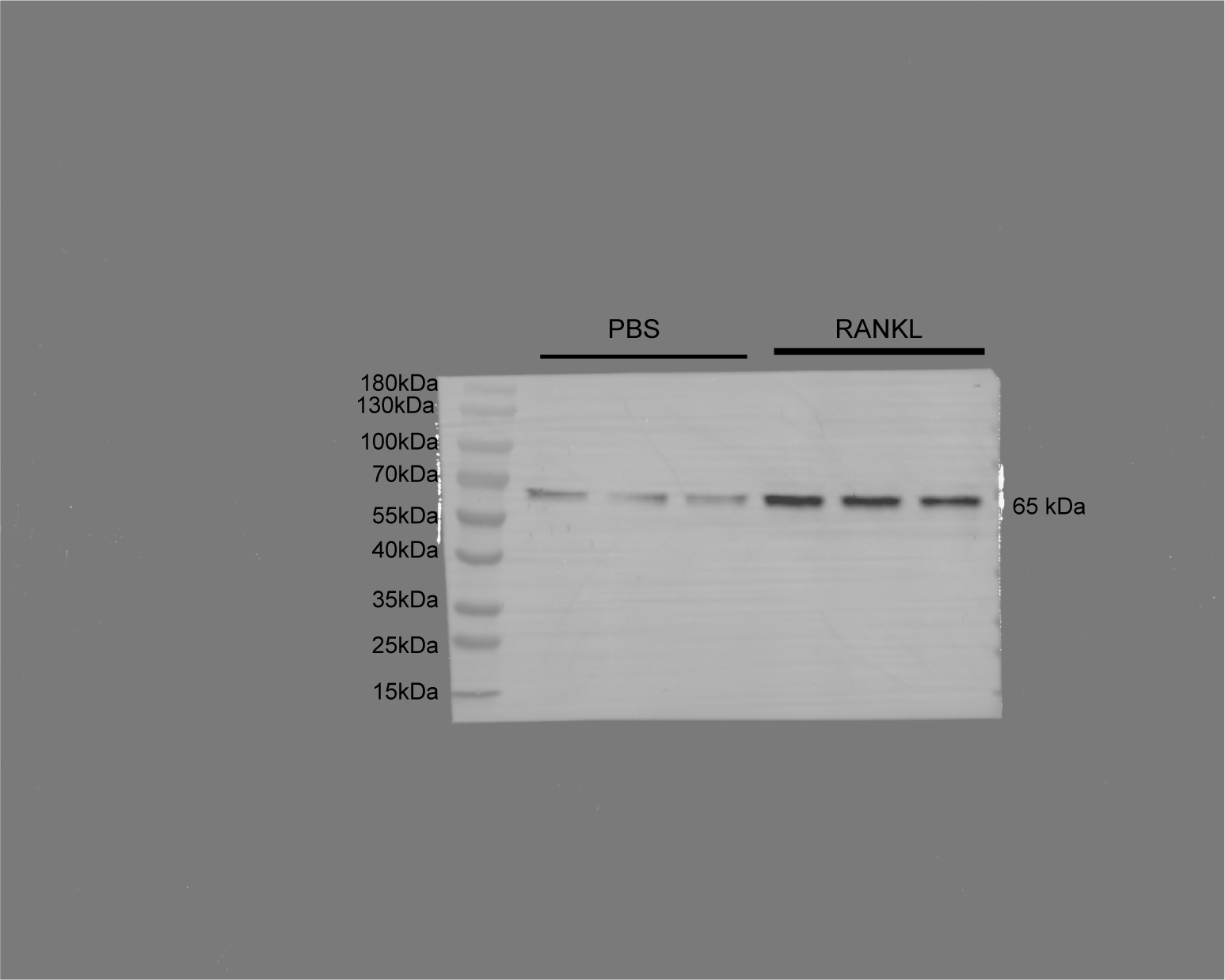

Supplement: Supplemental Information 2 [file peerj-12-18222-s002.zip › p65/6 pp65+p65 1_2(Composite)-01.tif]

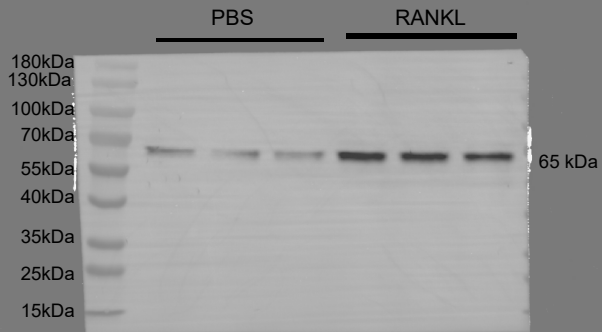

Supplement: Supplemental Information 2 [file peerj-12-18222-s002.zip › p65/6 pp65+p65 1_2(Composite).pdf]

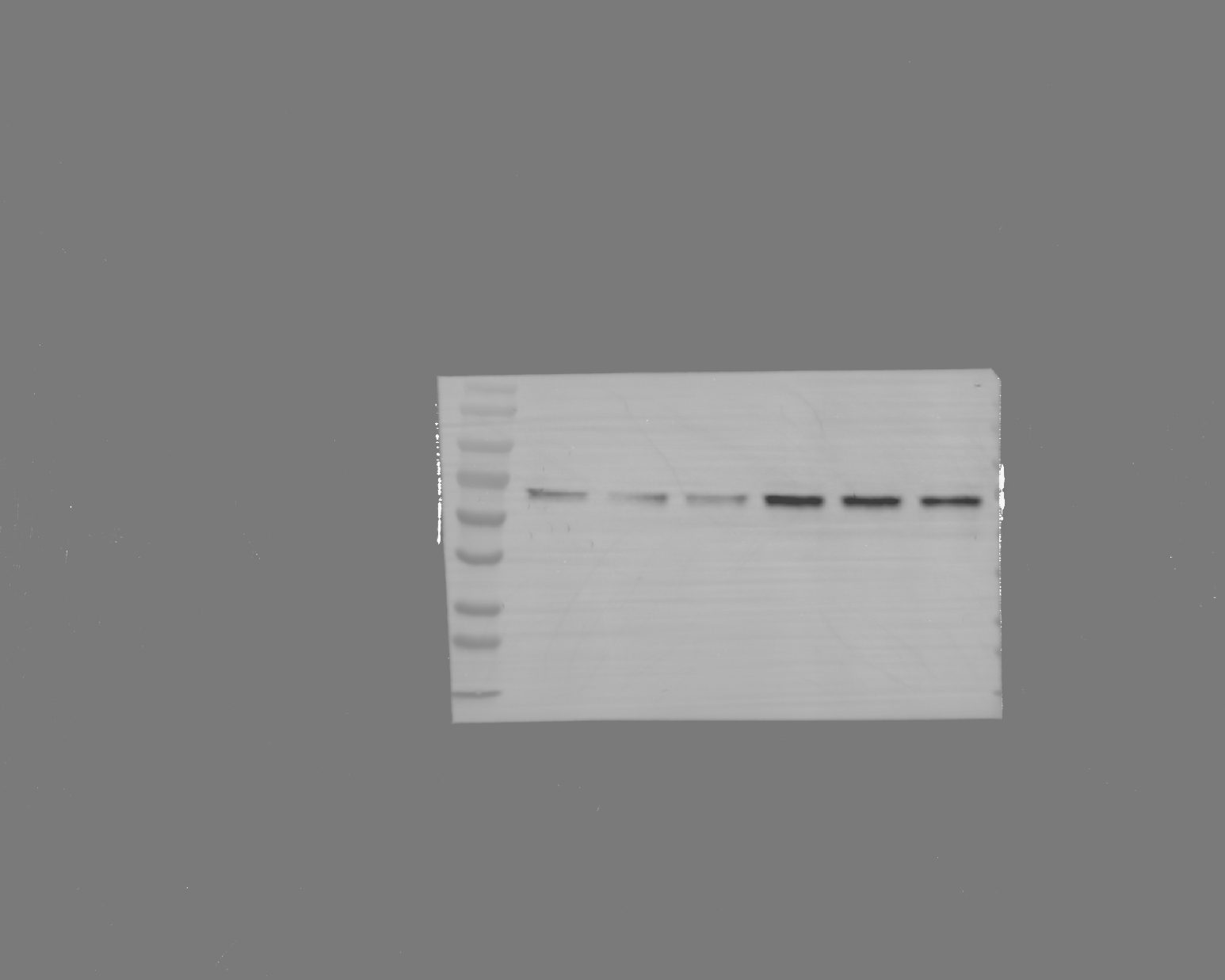

Supplement: Supplemental Information 2 [file peerj-12-18222-s002.zip › p65/6 pp65+p65 1_2(Composite).tif]

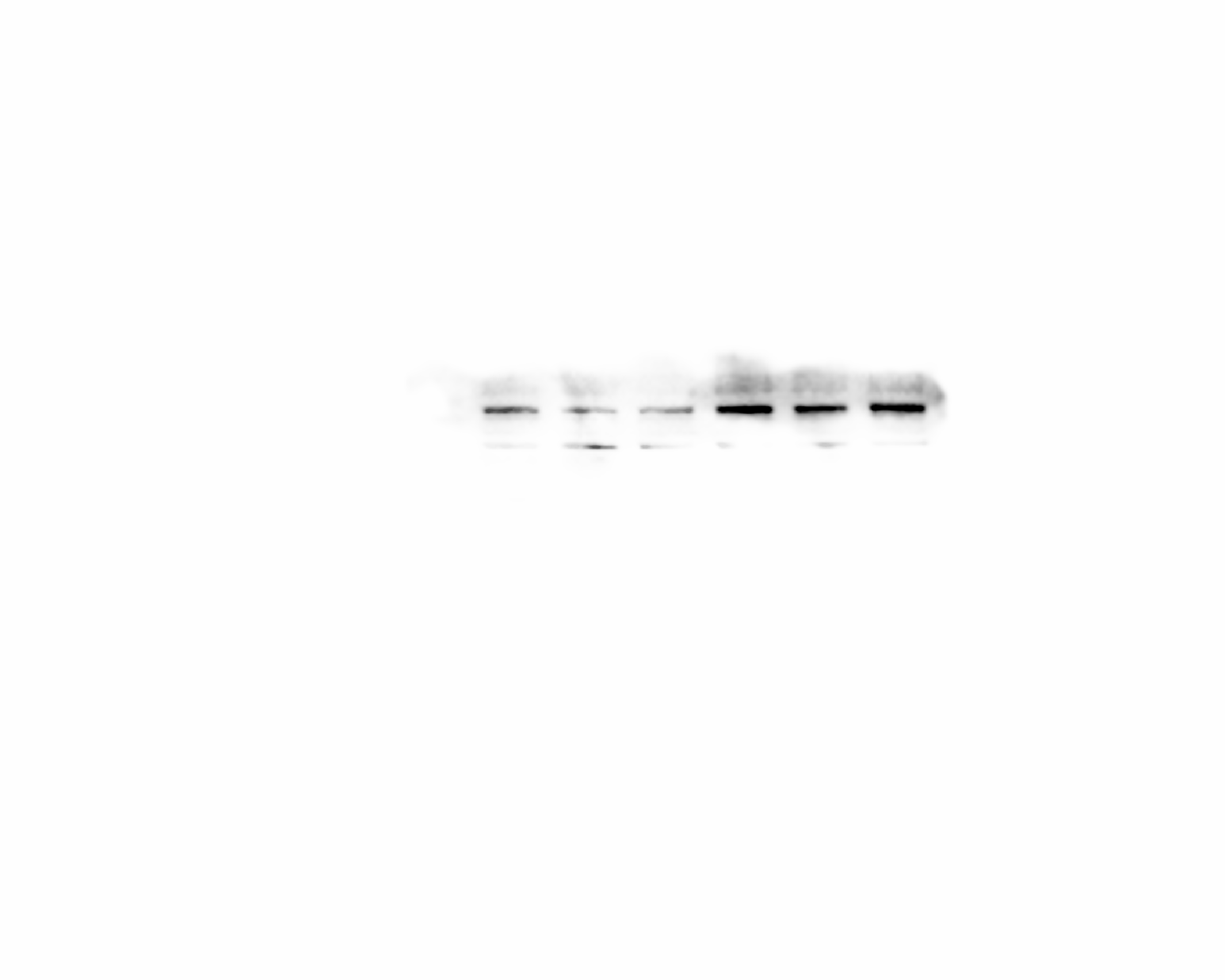

Supplement: Supplemental Information 2 [file peerj-12-18222-s002.zip › stat1/6 pstat1+stat11_1(Chemiluminescence).tif]

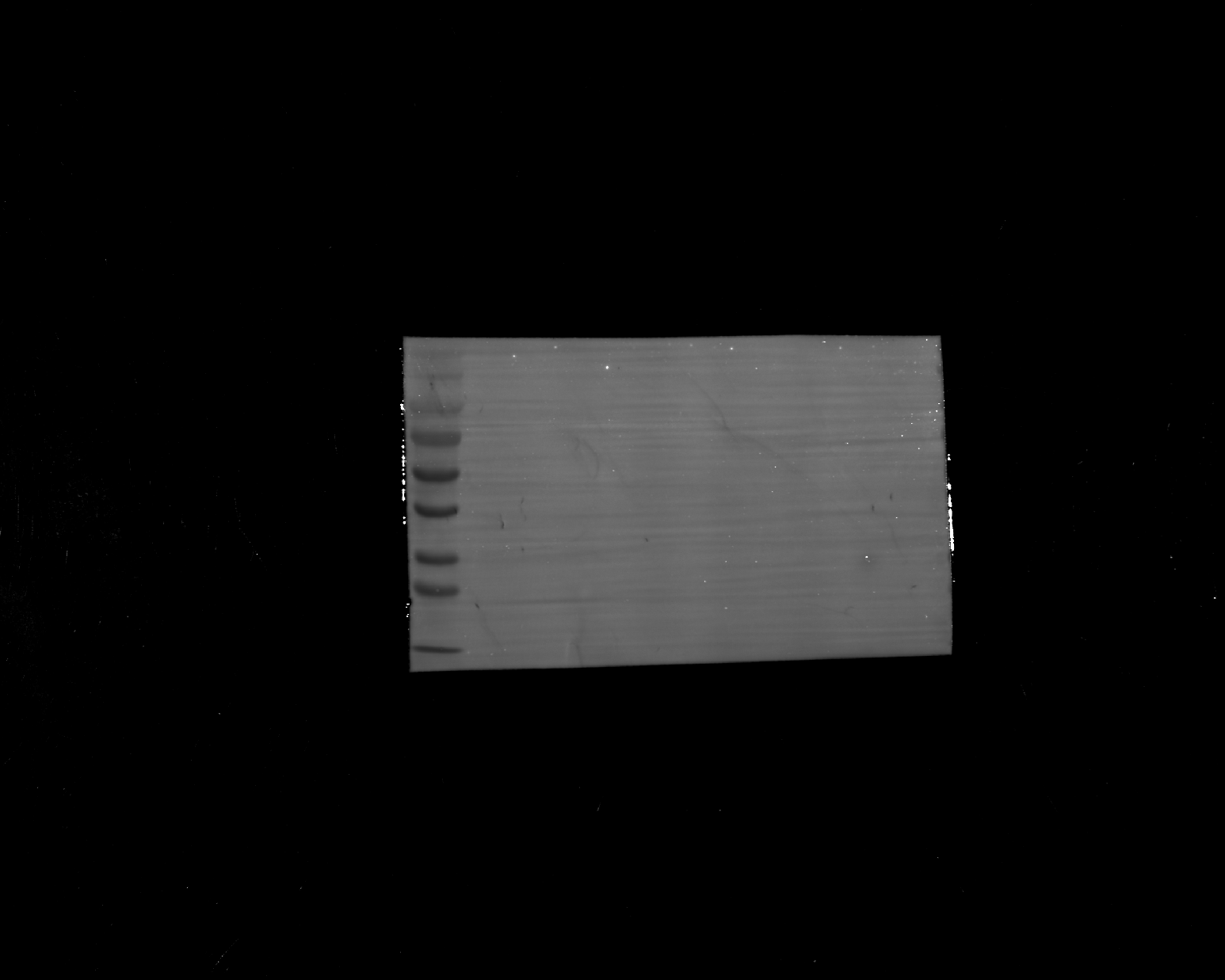

Supplement: Supplemental Information 2 [file peerj-12-18222-s002.zip › stat1/6 pstat1+stat11_1(Colorimetric).tif]

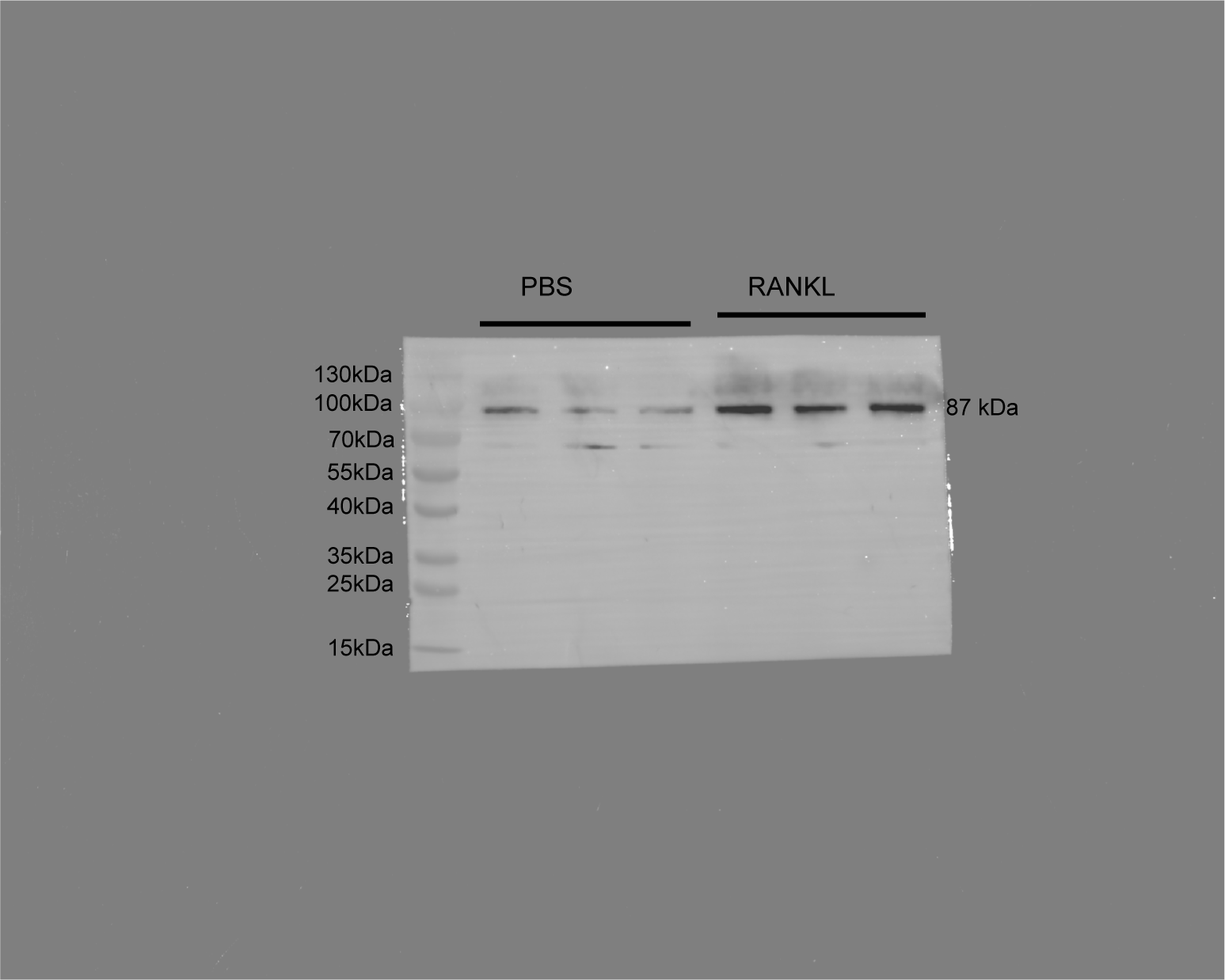

Supplement: Supplemental Information 2 [file peerj-12-18222-s002.zip › stat1/6 pstat1+stat11_1(Composite)-01.tif]

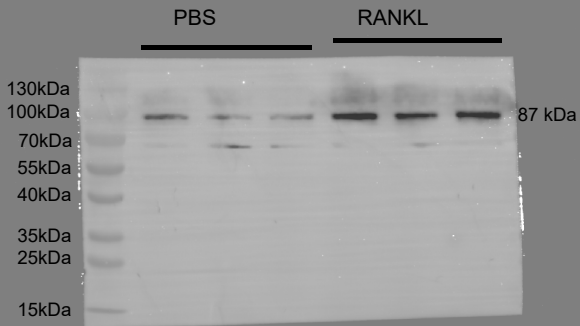

Supplement: Supplemental Information 2 [file peerj-12-18222-s002.zip › stat1/6 pstat1+stat11_1(Composite).pdf]

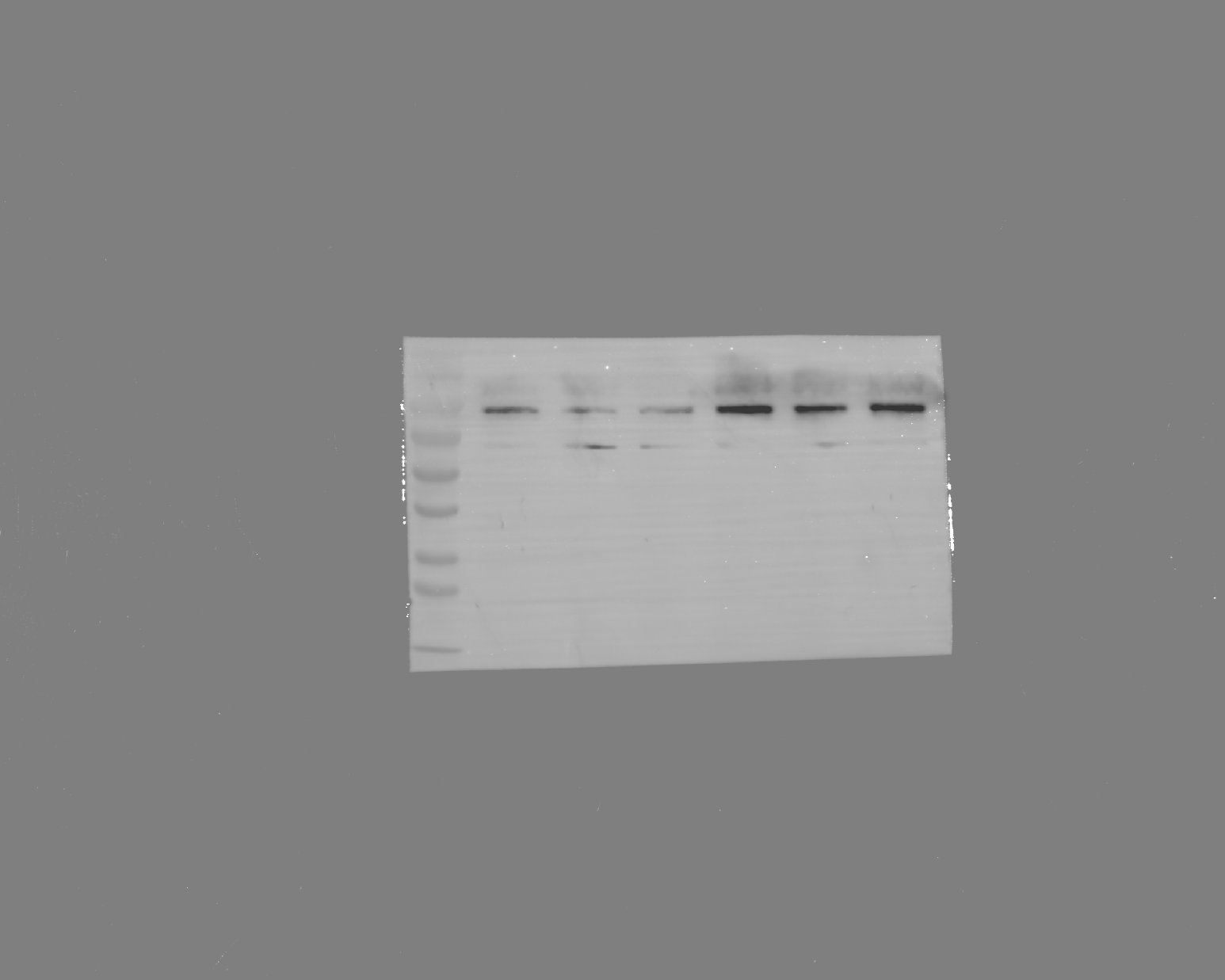

Supplement: Supplemental Information 2 [file peerj-12-18222-s002.zip › stat1/6 pstat1+stat11_1(Composite).tif]

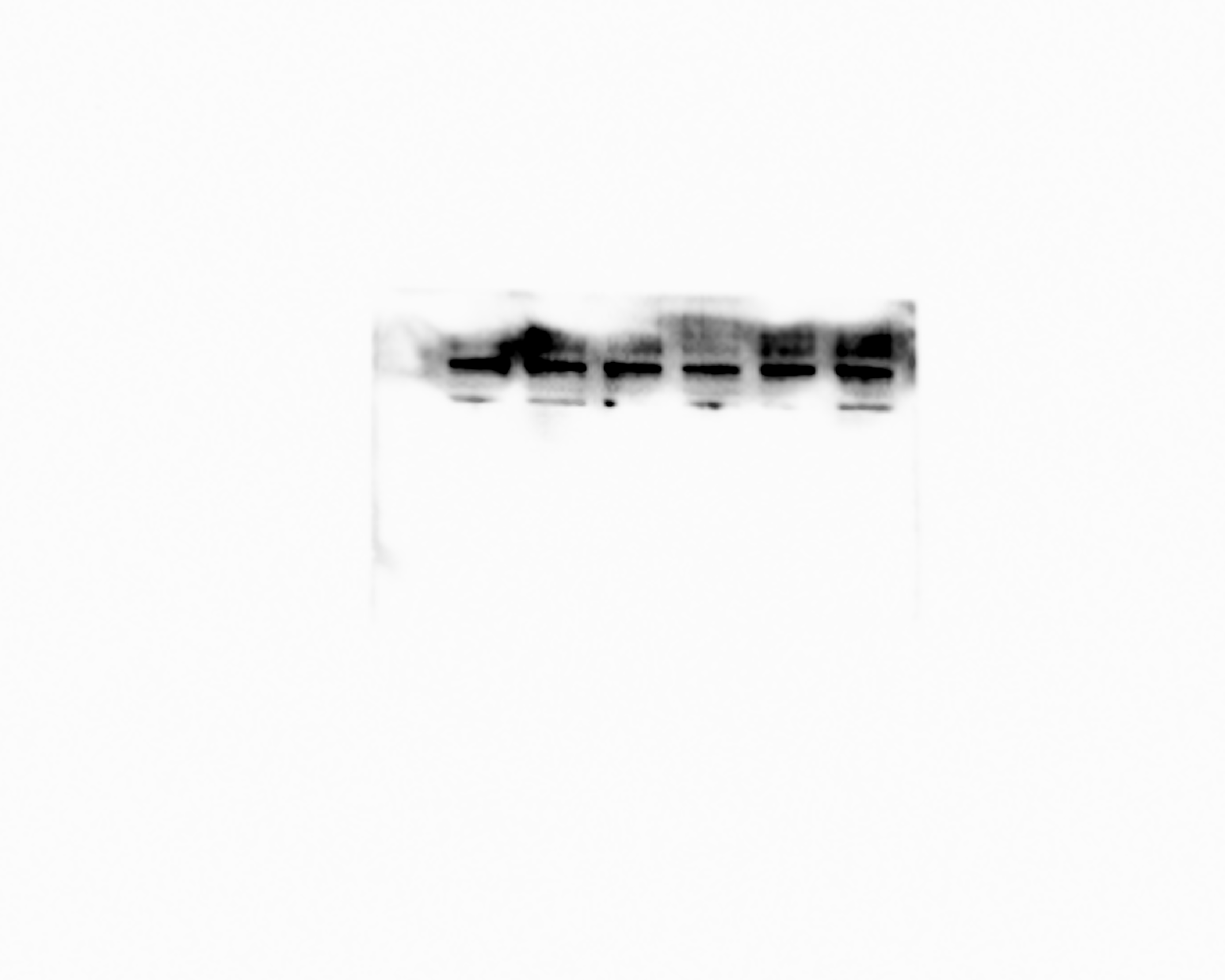

Supplement: Supplemental Information 2 [file peerj-12-18222-s002.zip › stat1/6 pstat1+stat11_2(Chemiluminescence).tif]

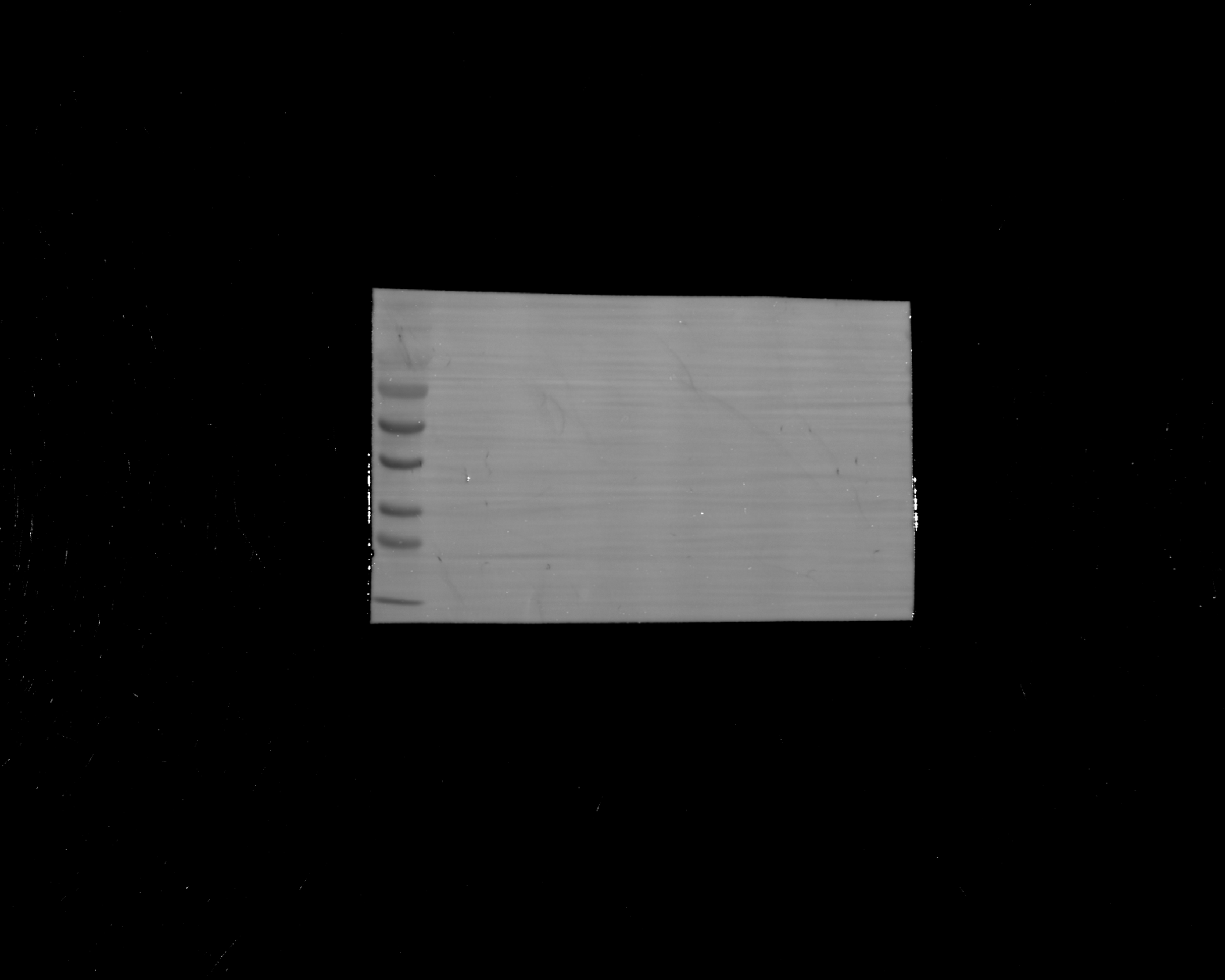

Supplement: Supplemental Information 2 [file peerj-12-18222-s002.zip › stat1/6 pstat1+stat11_2(Colorimetric).tif]

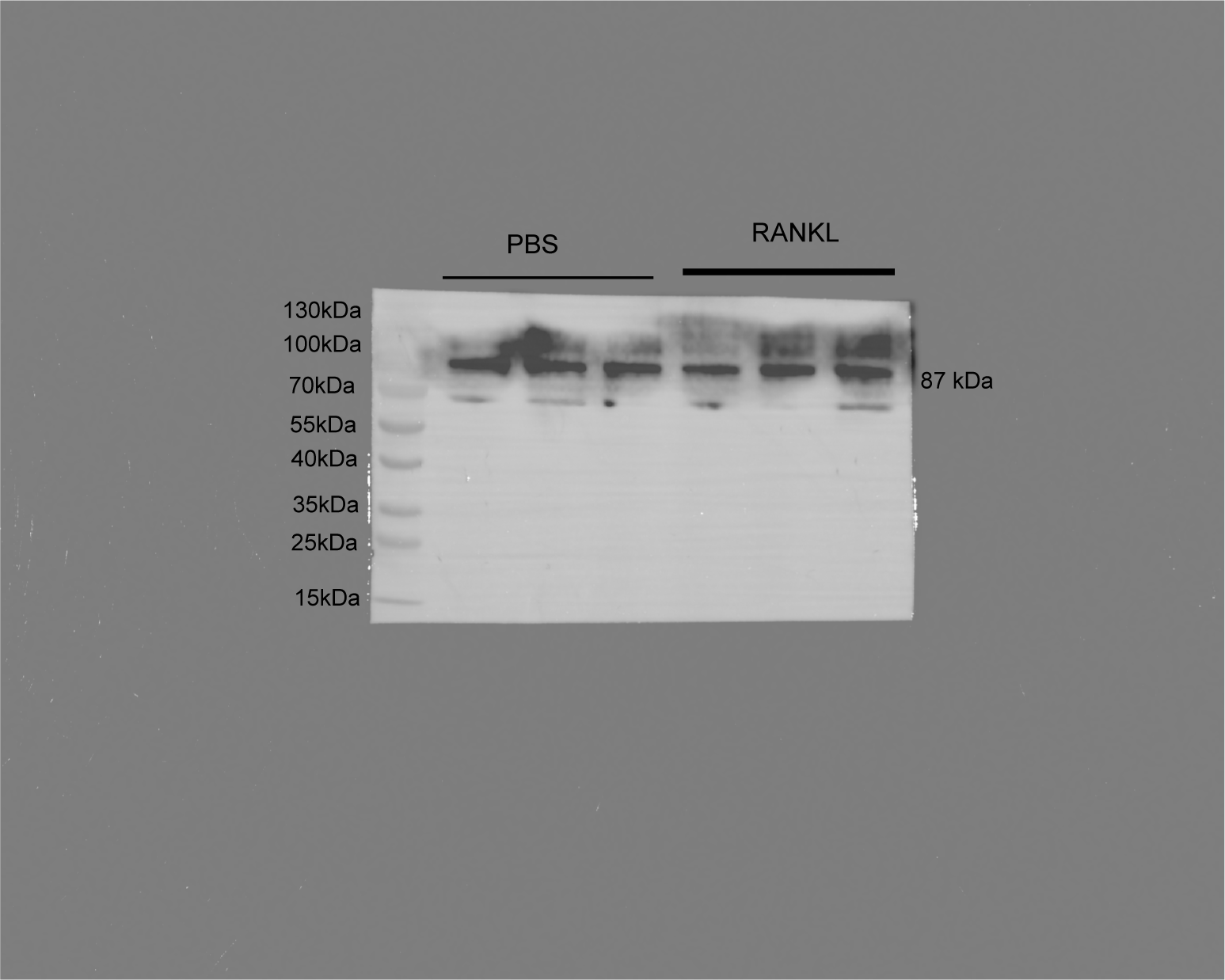

Supplement: Supplemental Information 2 [file peerj-12-18222-s002.zip › stat1/6 pstat1+stat11_2(Composite)-01.tif]

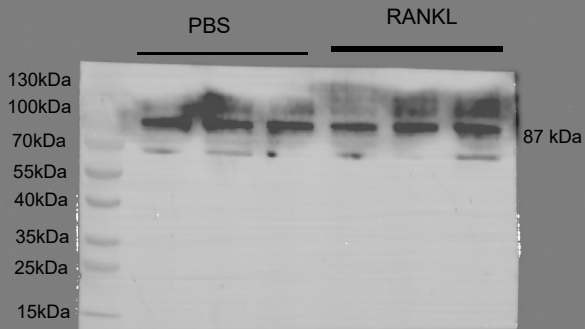

Supplement: Supplemental Information 2 [file peerj-12-18222-s002.zip › stat1/6 pstat1+stat11_2(Composite).pdf]

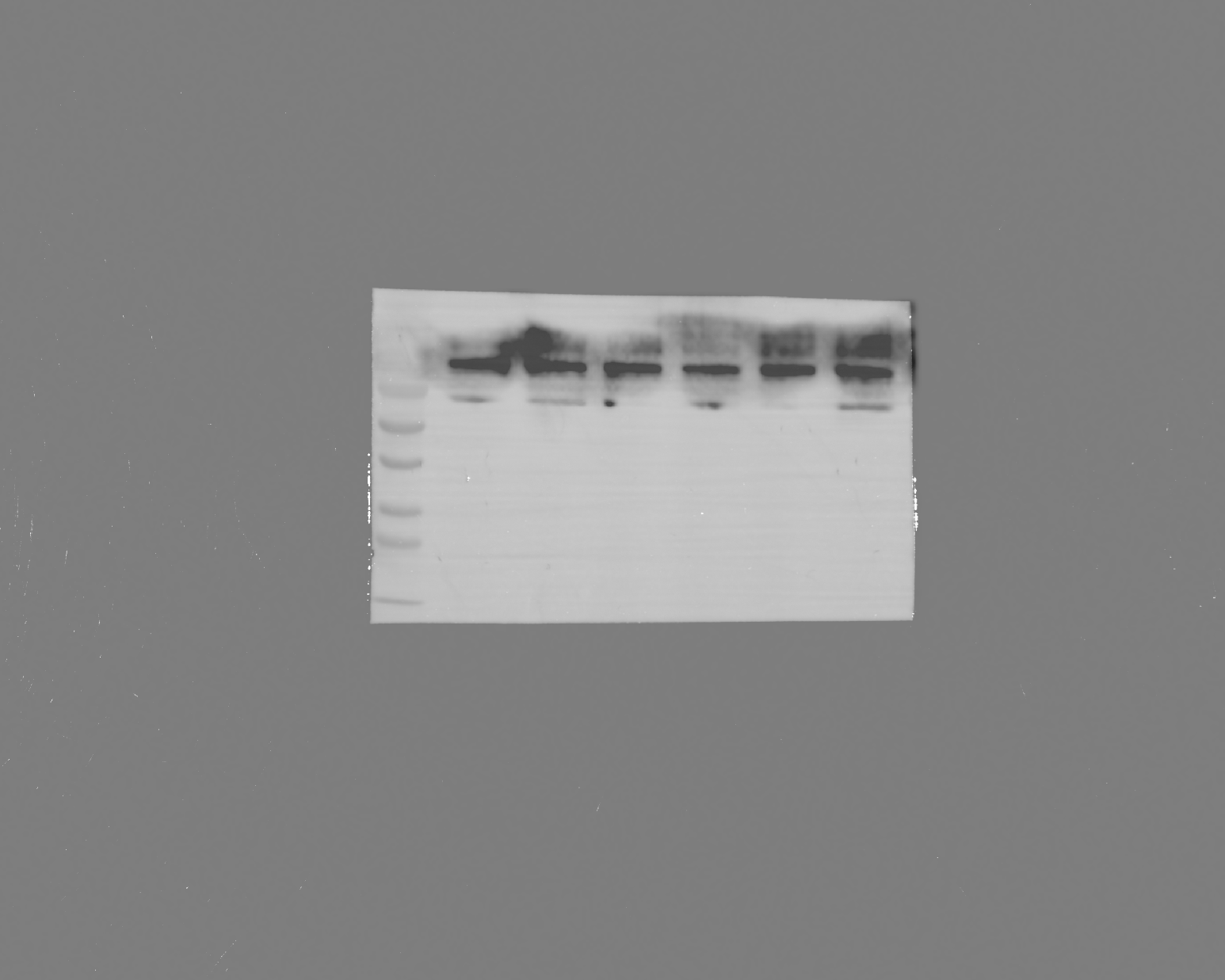

Supplement: Supplemental Information 2 [file peerj-12-18222-s002.zip › stat1/6 pstat1+stat11_2(Composite).tif]

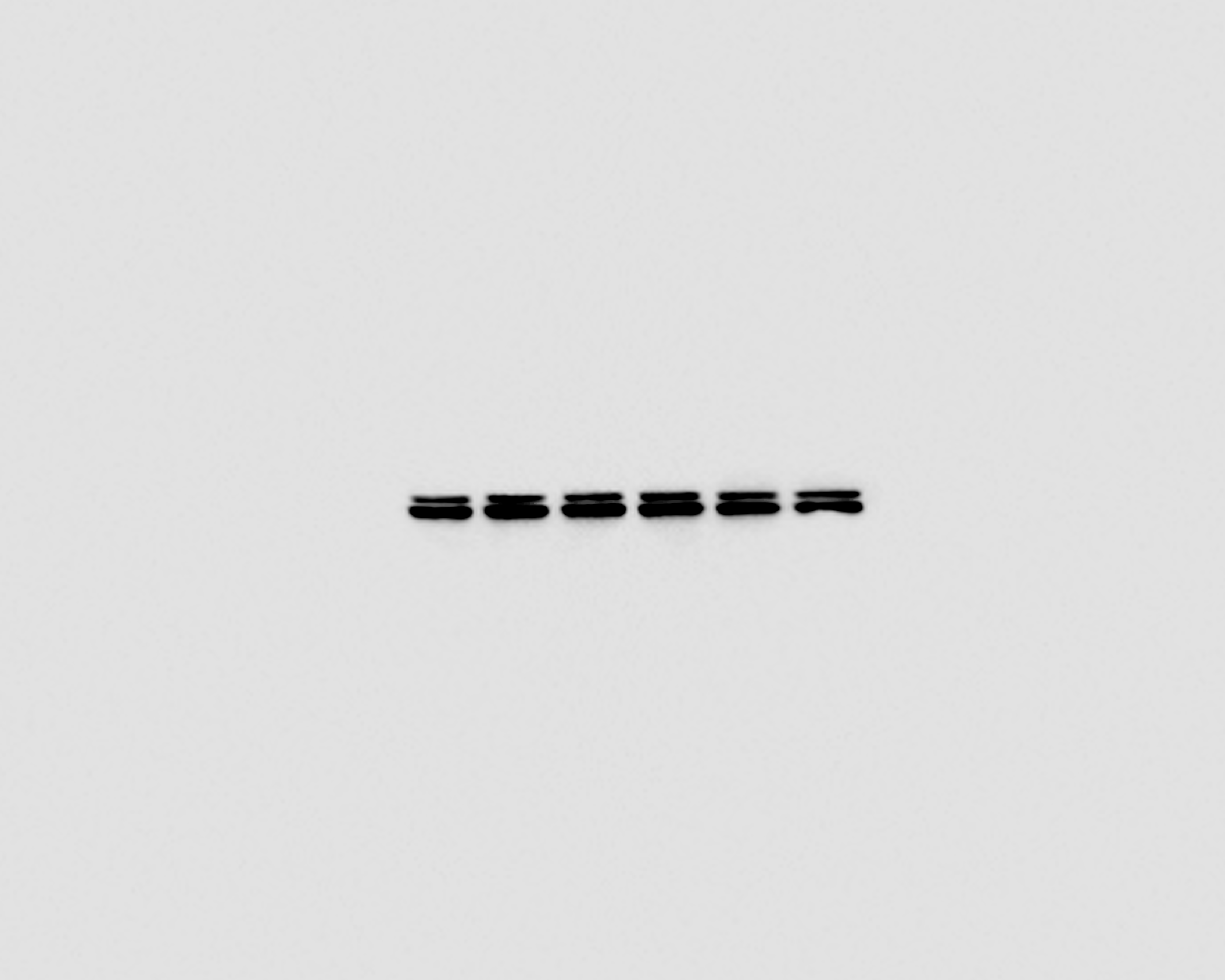

Supplement: Supplemental Information 3 [file peerj-12-18222-s003.zip › ERK/perk+erk 2_1(Chemiluminescence).tif]

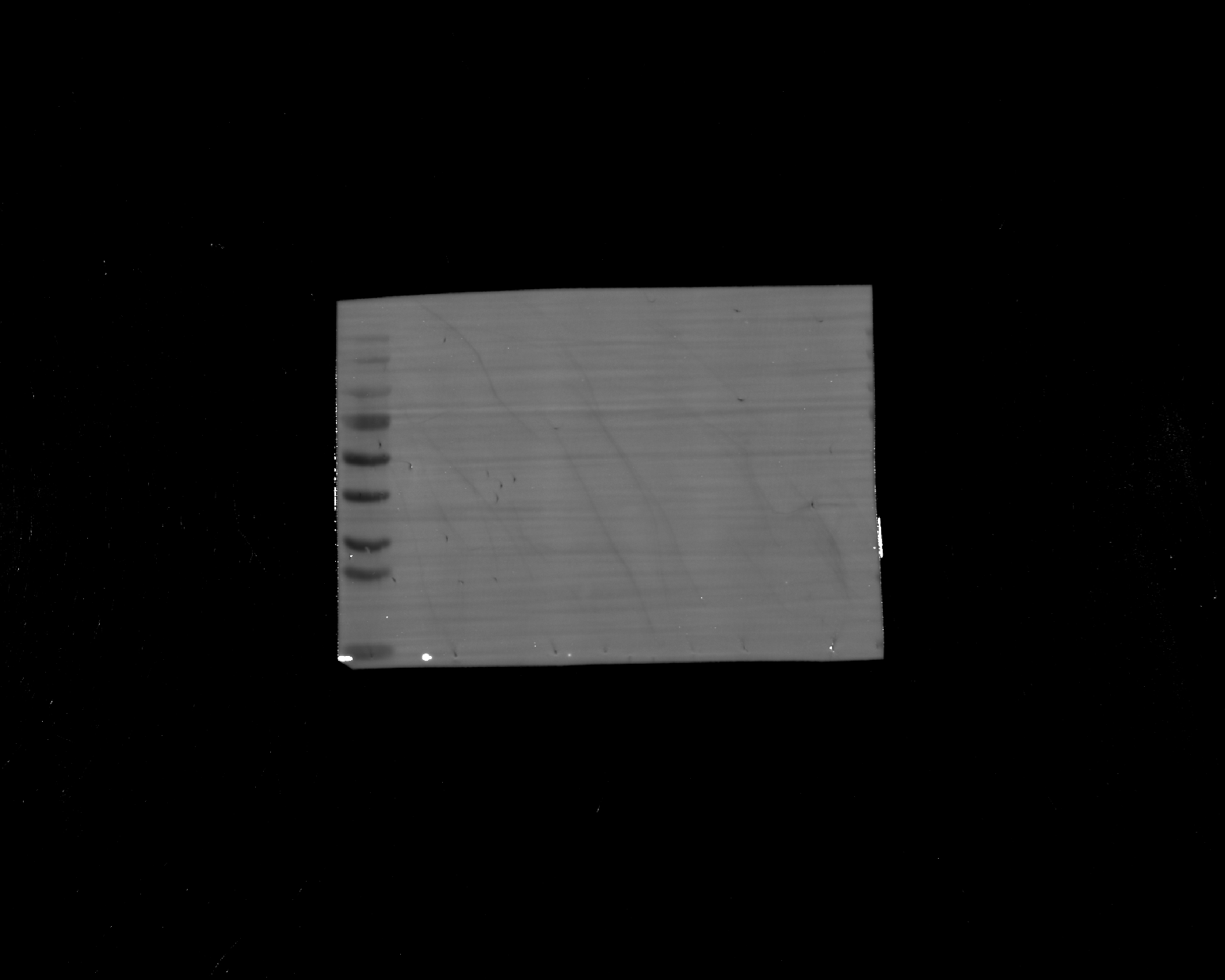

Supplement: Supplemental Information 3 [file peerj-12-18222-s003.zip › ERK/perk+erk 2_1(Colorimetric).tif]

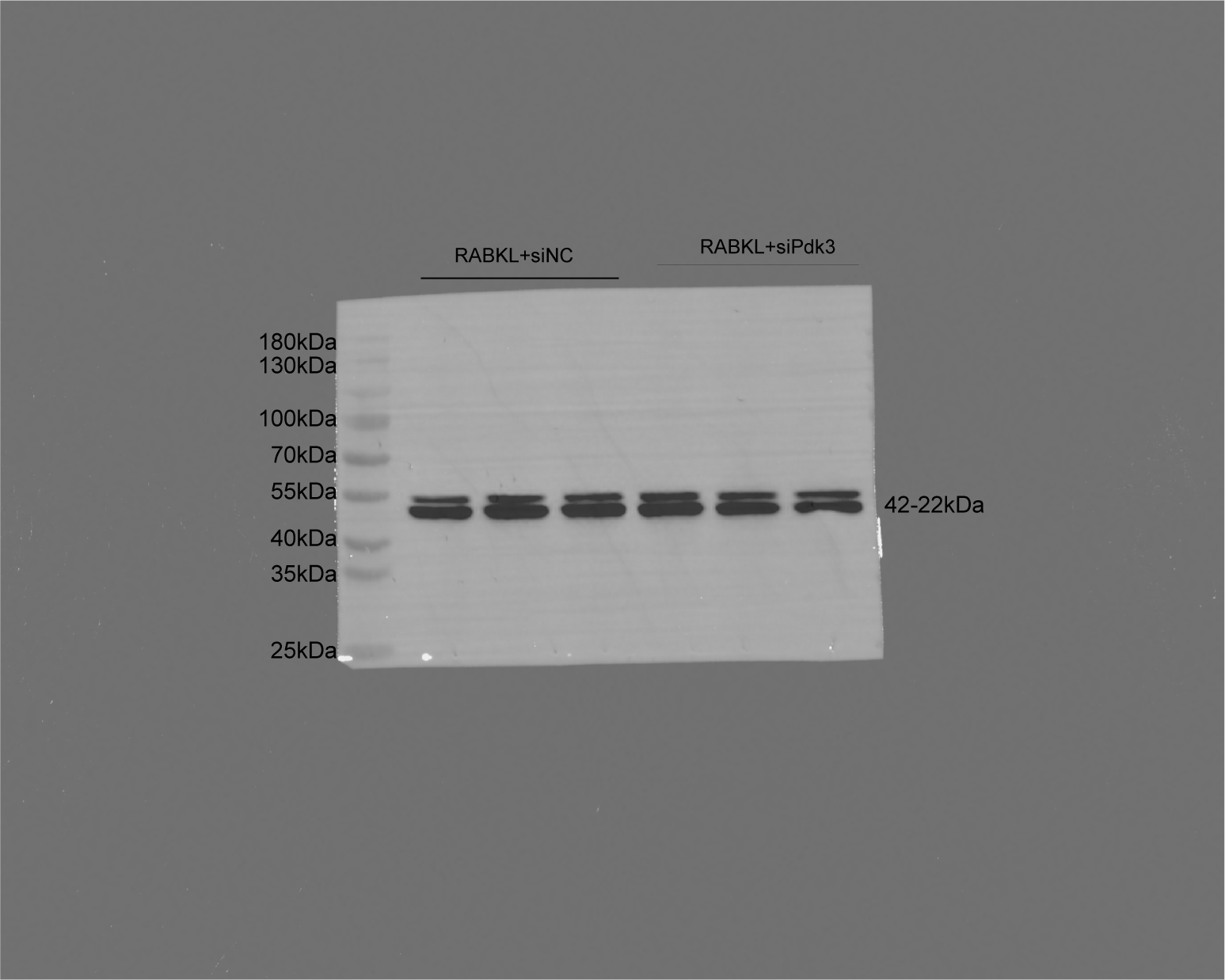

Supplement: Supplemental Information 3 [file peerj-12-18222-s003.zip › ERK/perk+erk 2_1(Composite)-01.tif]

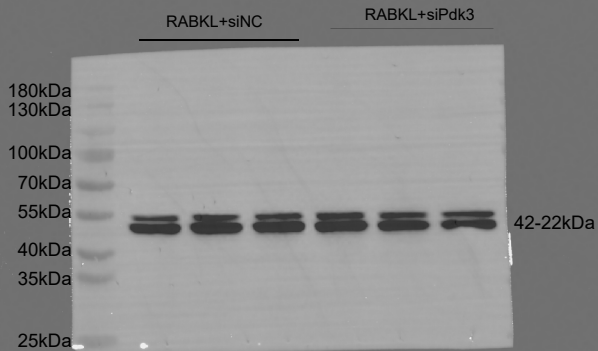

Supplement: Supplemental Information 3 [file peerj-12-18222-s003.zip › ERK/perk+erk 2_1(Composite).pdf]

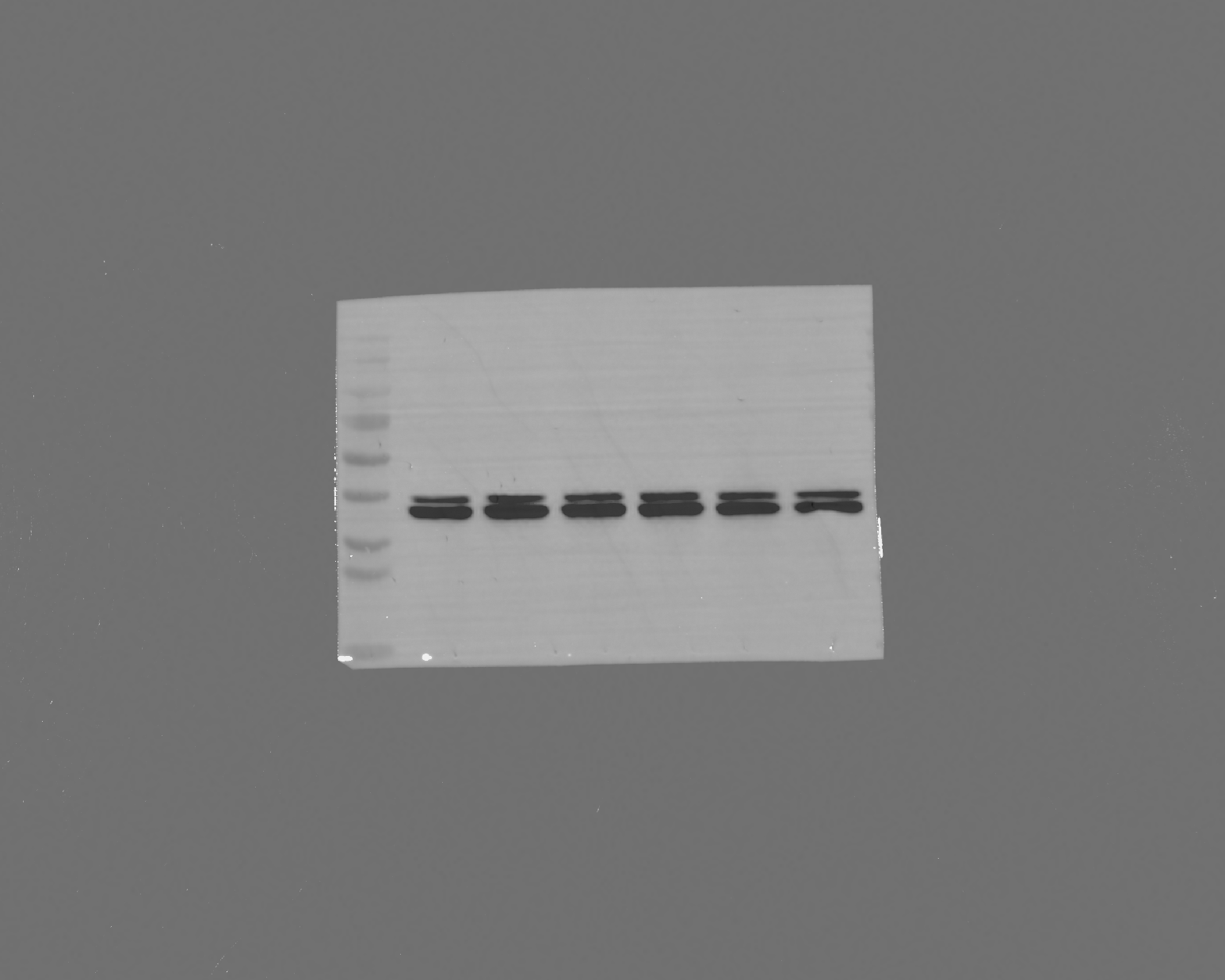

Supplement: Supplemental Information 3 [file peerj-12-18222-s003.zip › ERK/perk+erk 2_1(Composite).tif]

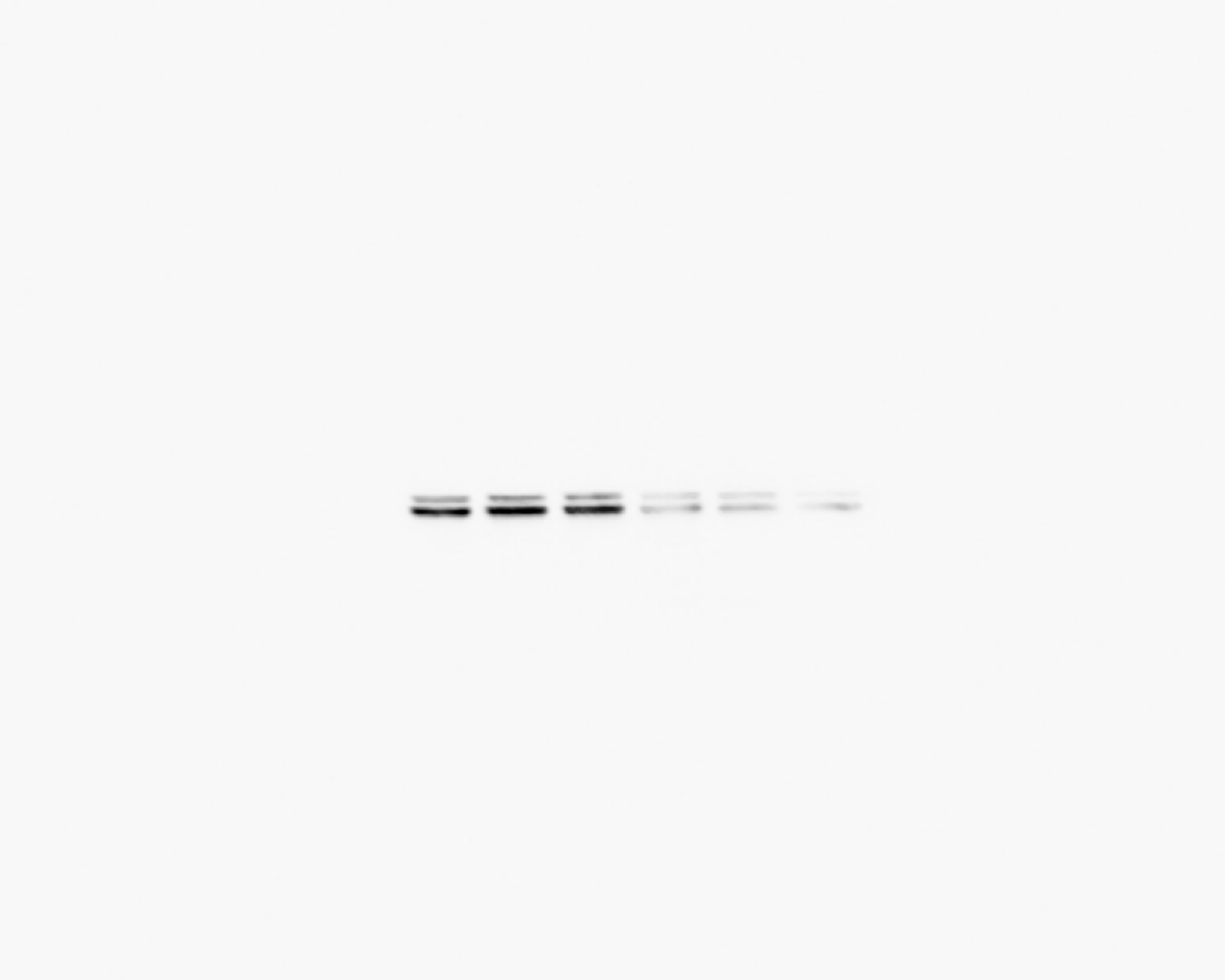

Supplement: Supplemental Information 3 [file peerj-12-18222-s003.zip › ERK/perk+erk 2_2(Chemiluminescence).tif]

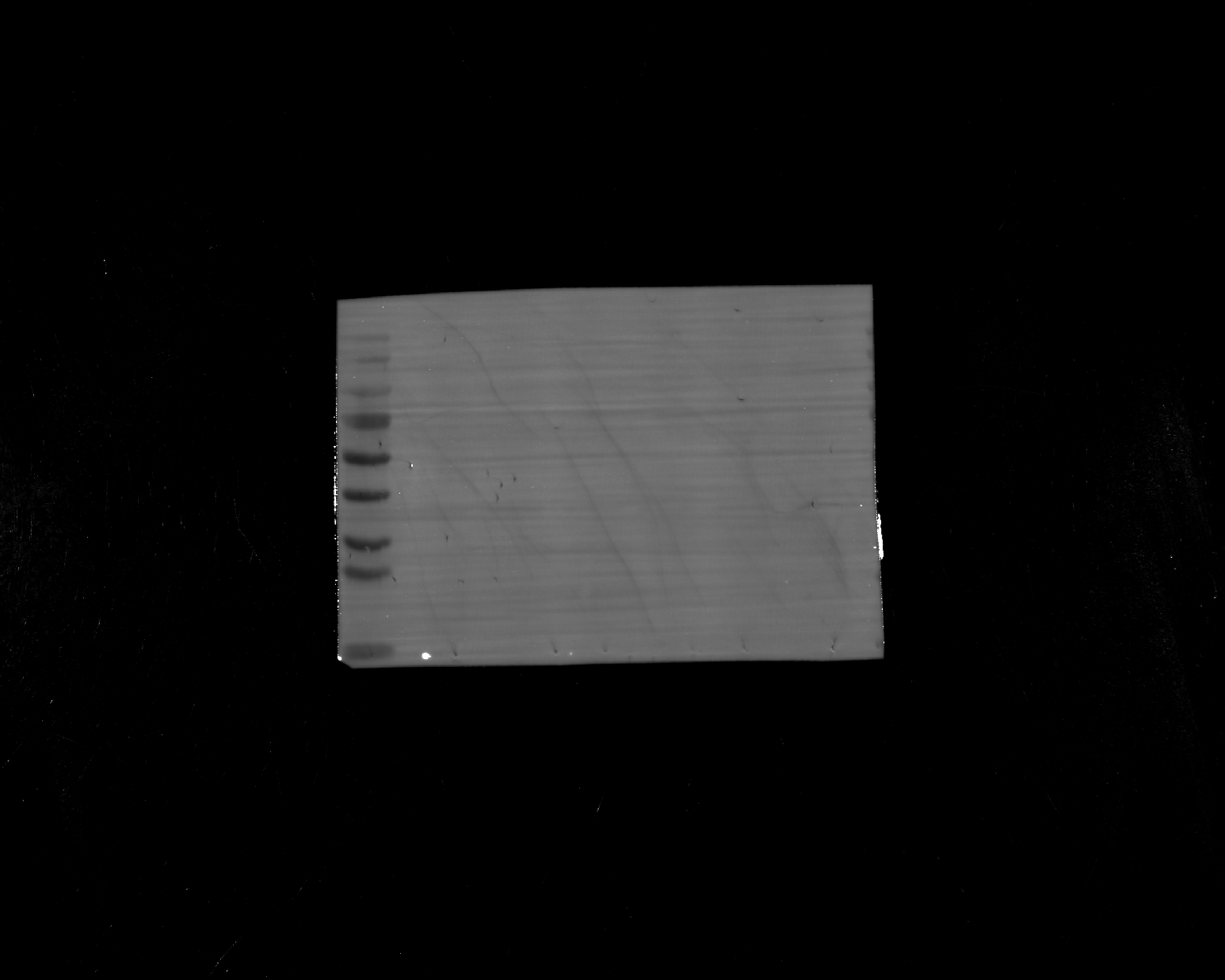

Supplement: Supplemental Information 3 [file peerj-12-18222-s003.zip › ERK/perk+erk 2_2(Colorimetric).tif]

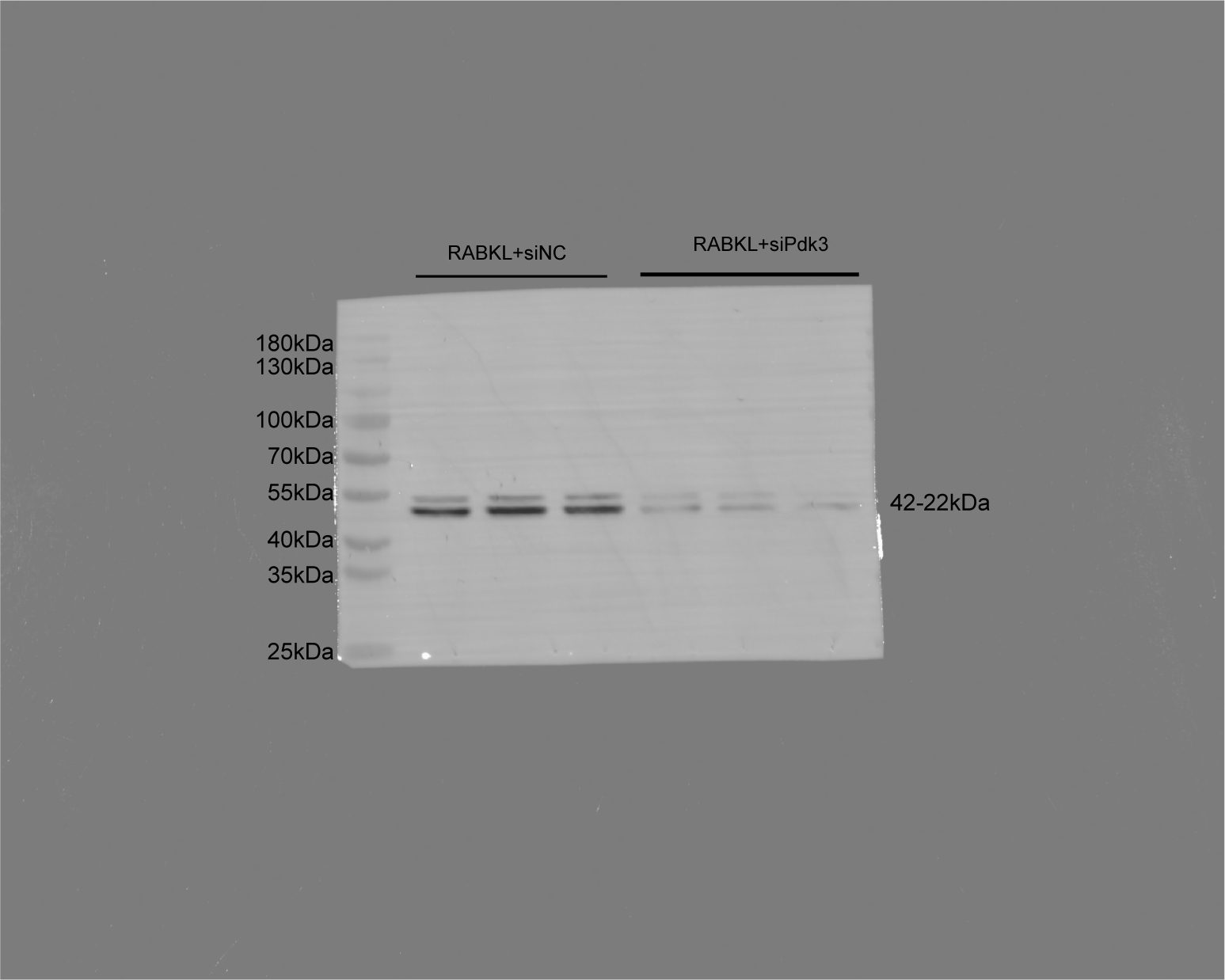

Supplement: Supplemental Information 3 [file peerj-12-18222-s003.zip › ERK/perk+erk 2_2(Composite)-01.tif]

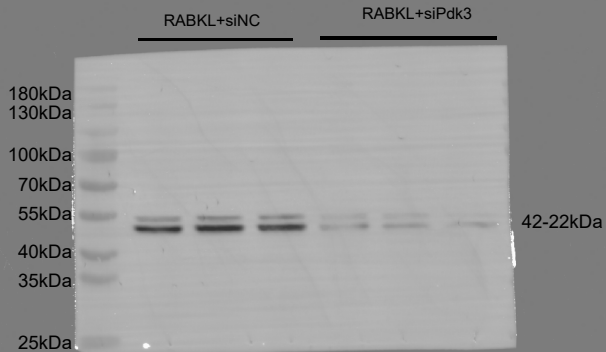

Supplement: Supplemental Information 3 [file peerj-12-18222-s003.zip › ERK/perk+erk 2_2(Composite).pdf]

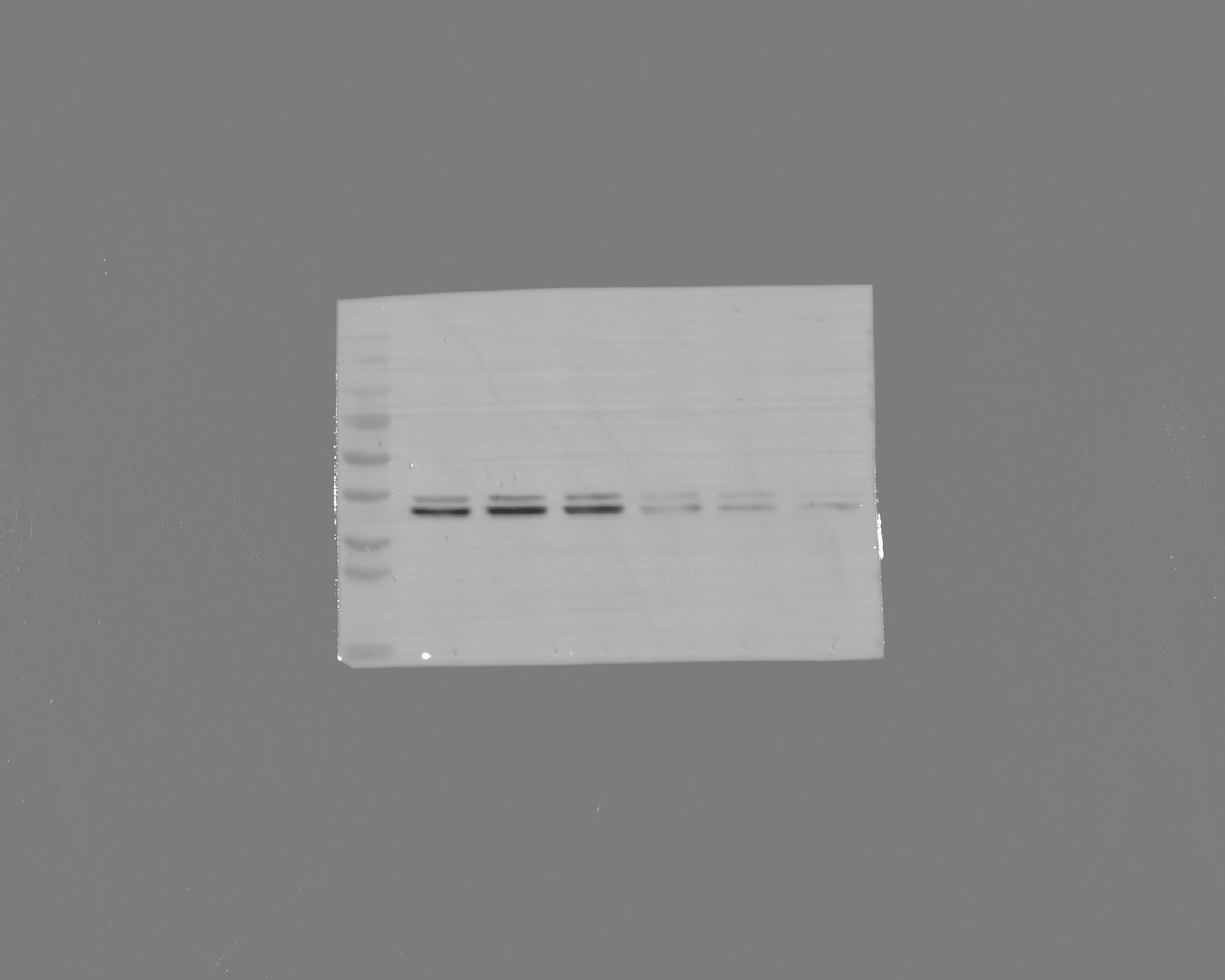

Supplement: Supplemental Information 3 [file peerj-12-18222-s003.zip › ERK/perk+erk 2_2(Composite).tif]

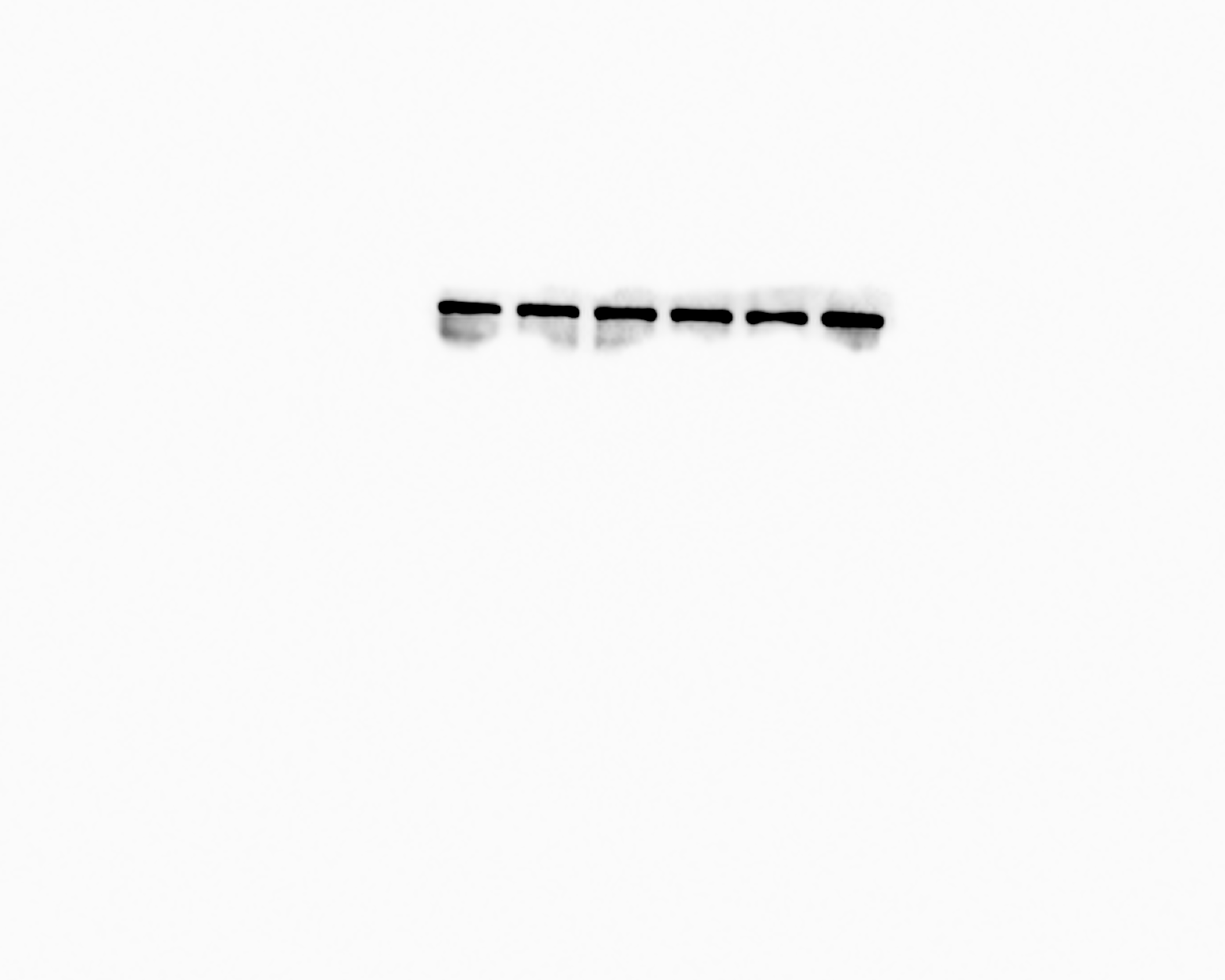

Supplement: Supplemental Information 3 [file peerj-12-18222-s003.zip › JAK1/pjak1+jak1 1_1(Chemiluminescence).tif]

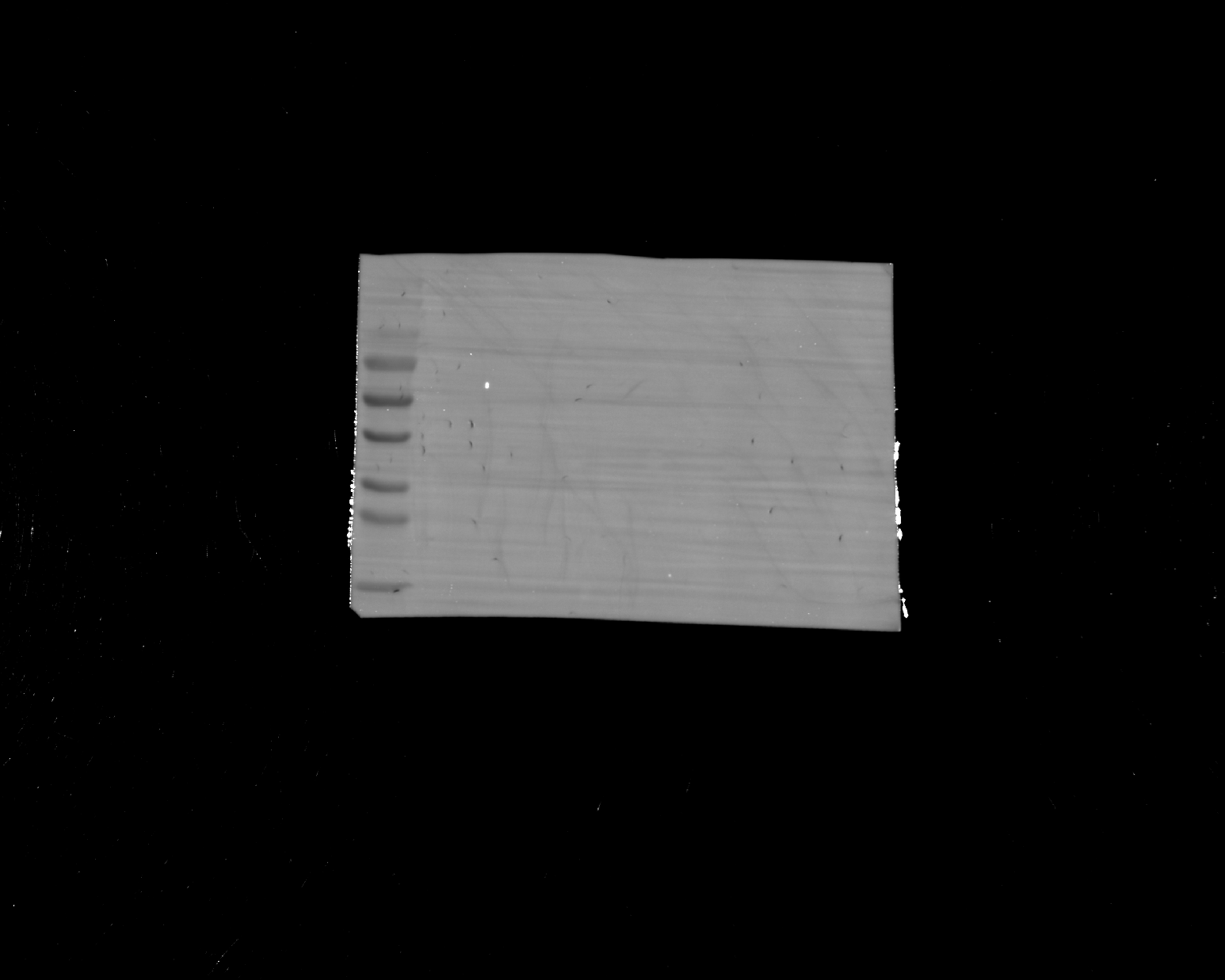

Supplement: Supplemental Information 3 [file peerj-12-18222-s003.zip › JAK1/pjak1+jak1 1_1(Colorimetric).tif]

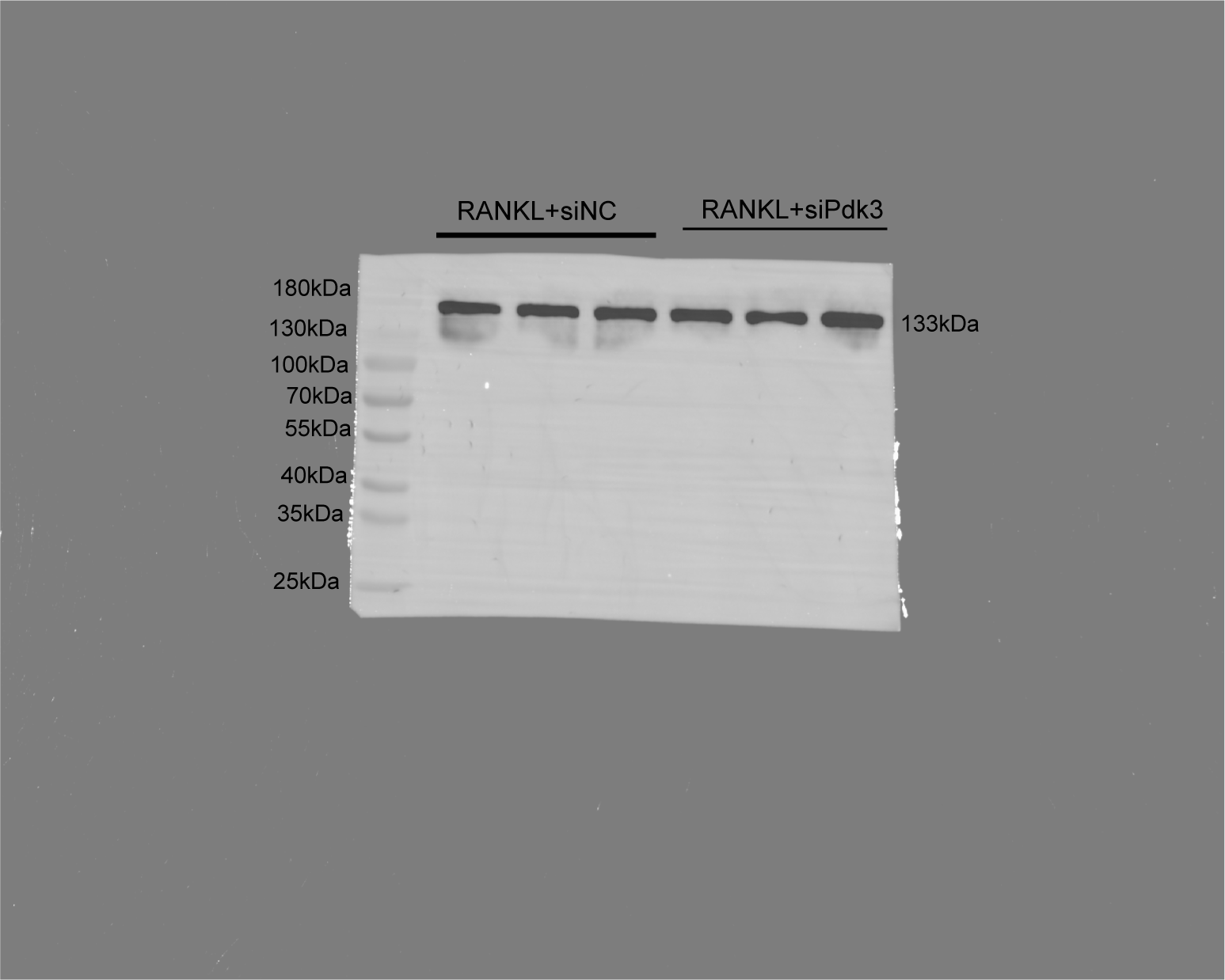

Supplement: Supplemental Information 3 [file peerj-12-18222-s003.zip › JAK1/pjak1+jak1 1_1(Composite)-01.tif]

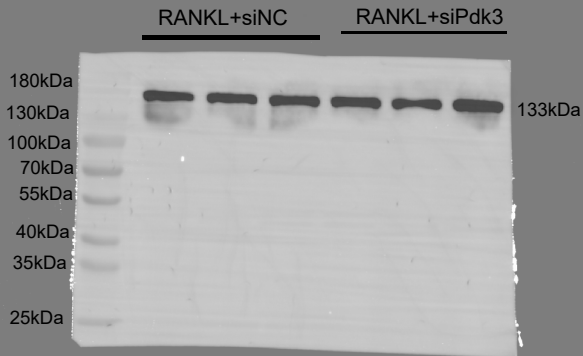

Supplement: Supplemental Information 3 [file peerj-12-18222-s003.zip › JAK1/pjak1+jak1 1_1(Composite).pdf]

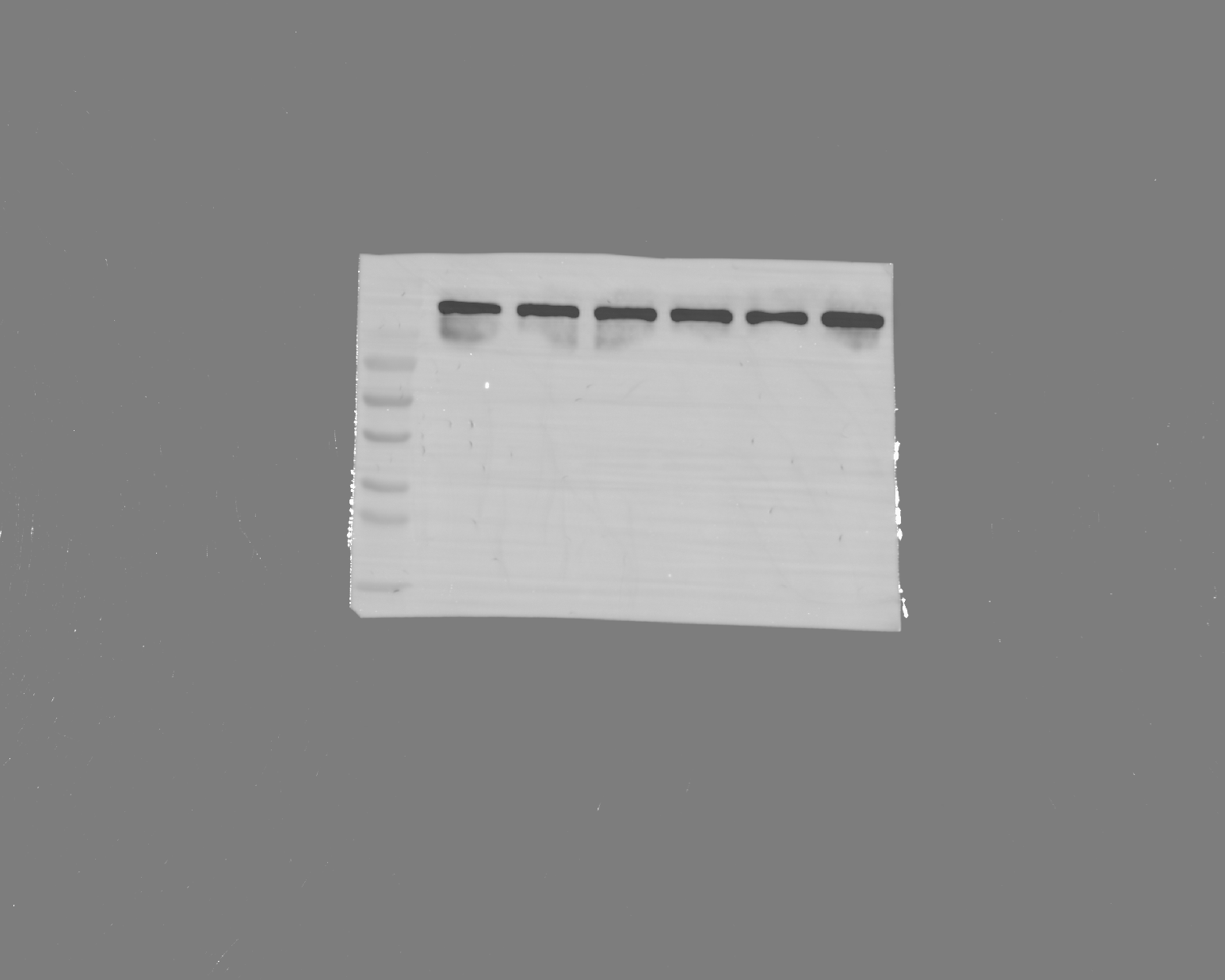

Supplement: Supplemental Information 3 [file peerj-12-18222-s003.zip › JAK1/pjak1+jak1 1_1(Composite).tif]

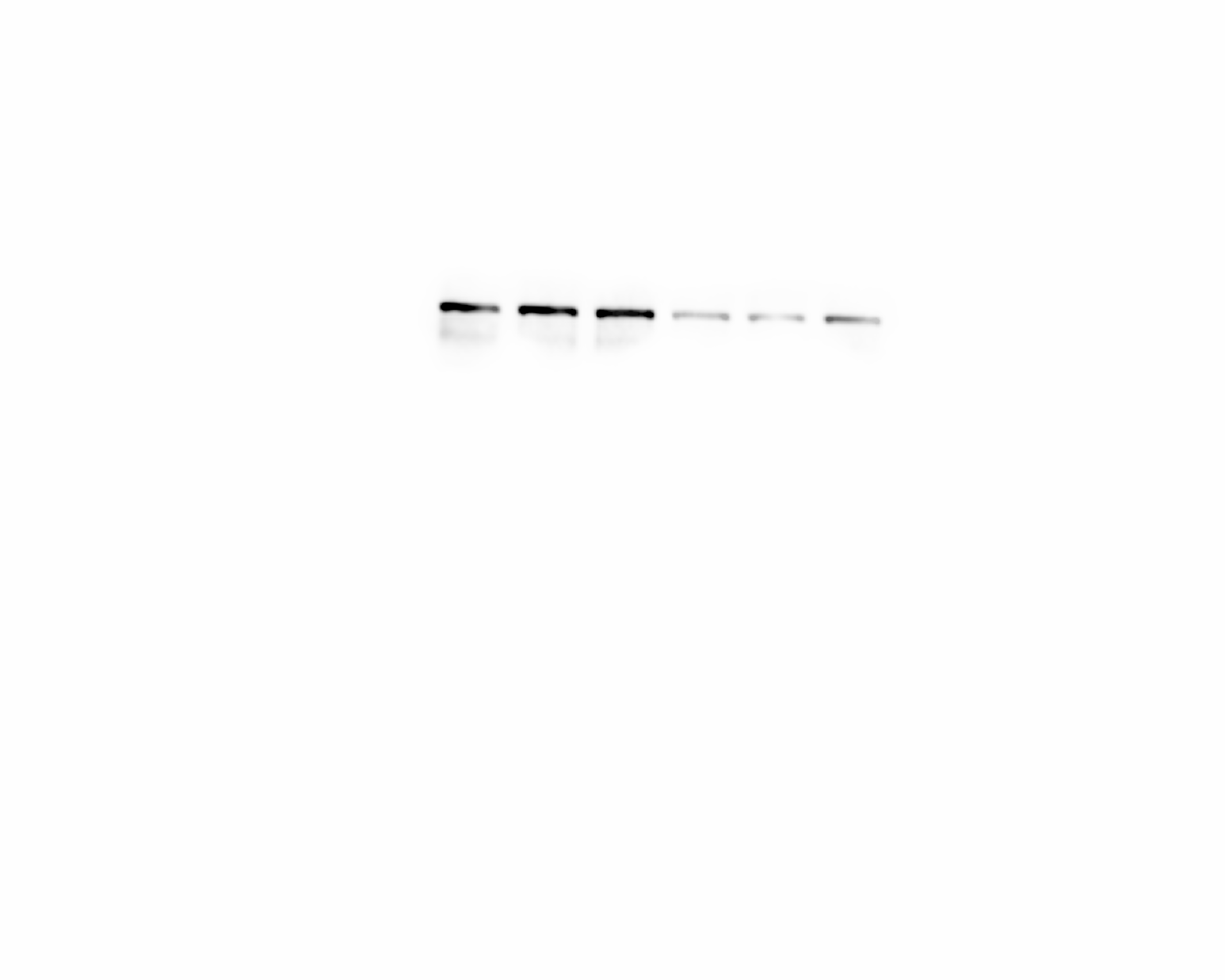

Supplement: Supplemental Information 3 [file peerj-12-18222-s003.zip › JAK1/pjak1+jak1 1_2(Chemiluminescence).tif]

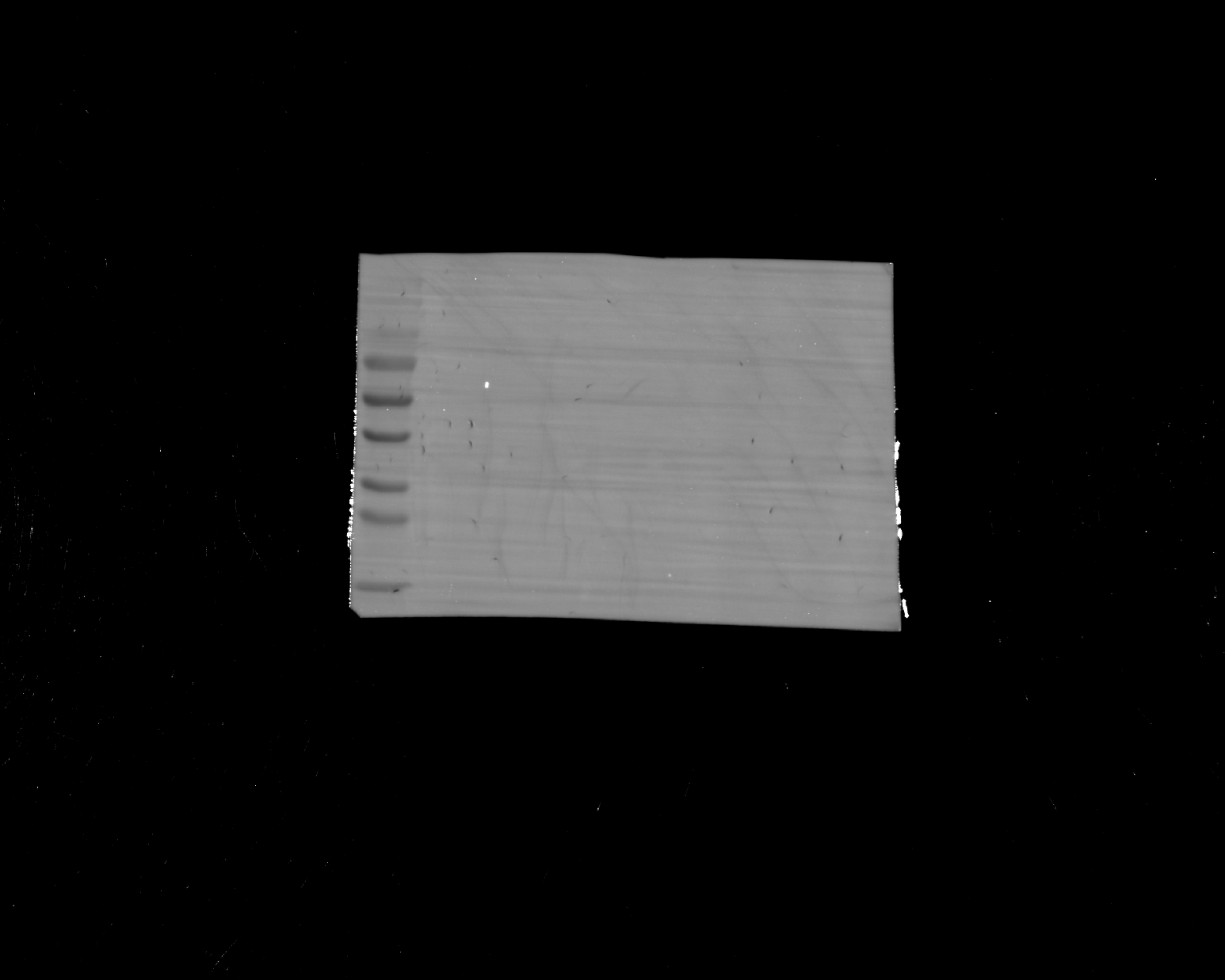

Supplement: Supplemental Information 3 [file peerj-12-18222-s003.zip › JAK1/pjak1+jak1 1_2(Colorimetric).tif]

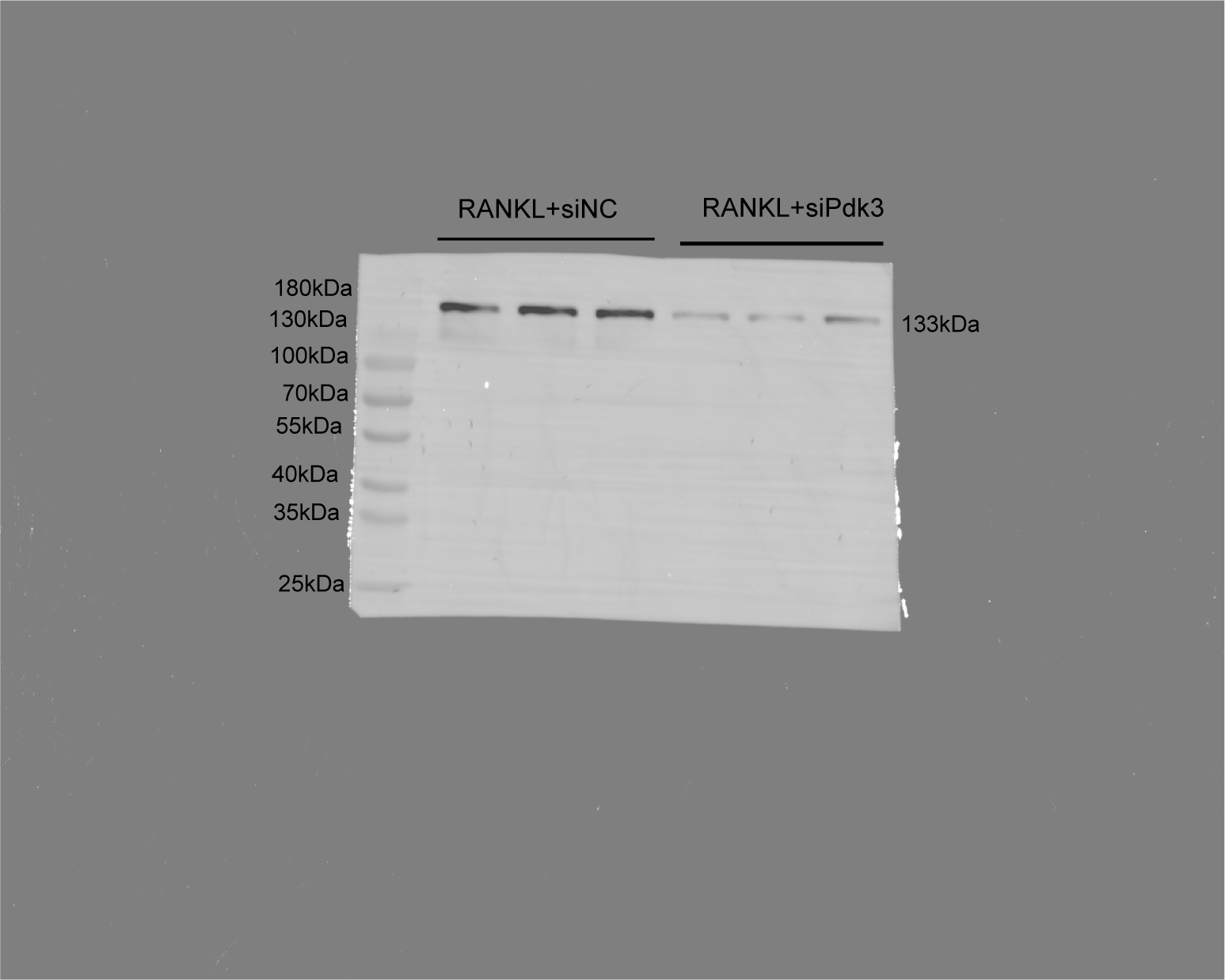

Supplement: Supplemental Information 3 [file peerj-12-18222-s003.zip › JAK1/pjak1+jak1 1_2(Composite)-01.tif]

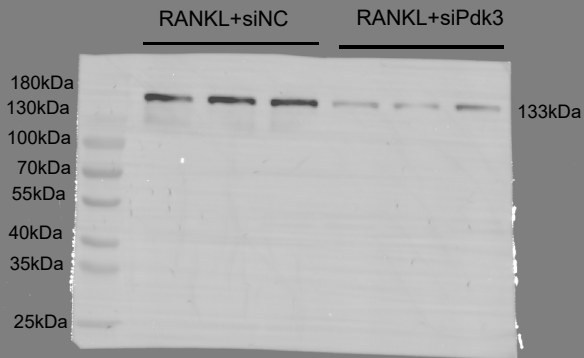

Supplement: Supplemental Information 3 [file peerj-12-18222-s003.zip › JAK1/pjak1+jak1 1_2(Composite).pdf]

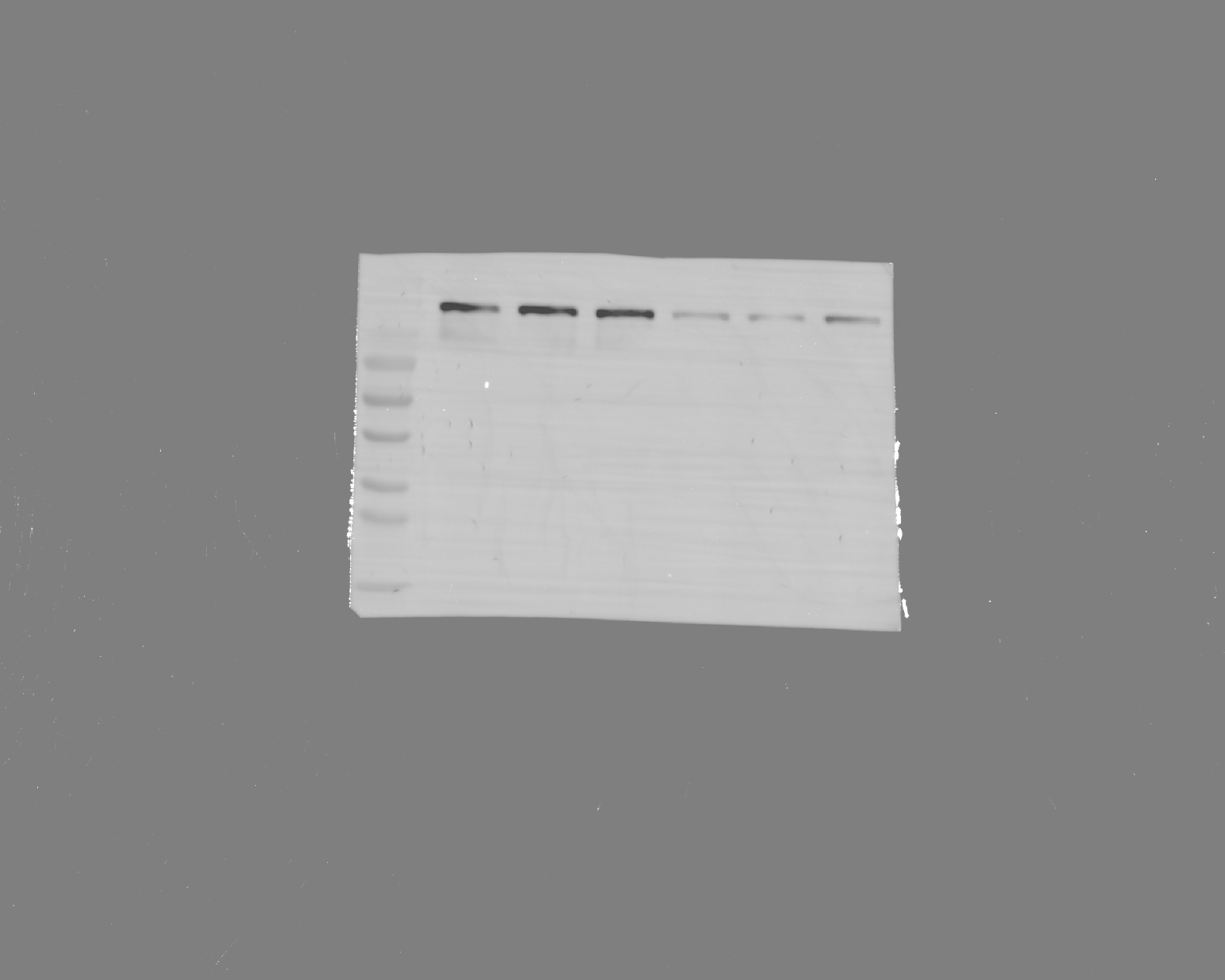

Supplement: Supplemental Information 3 [file peerj-12-18222-s003.zip › JAK1/pjak1+jak1 1_2(Composite).tif]

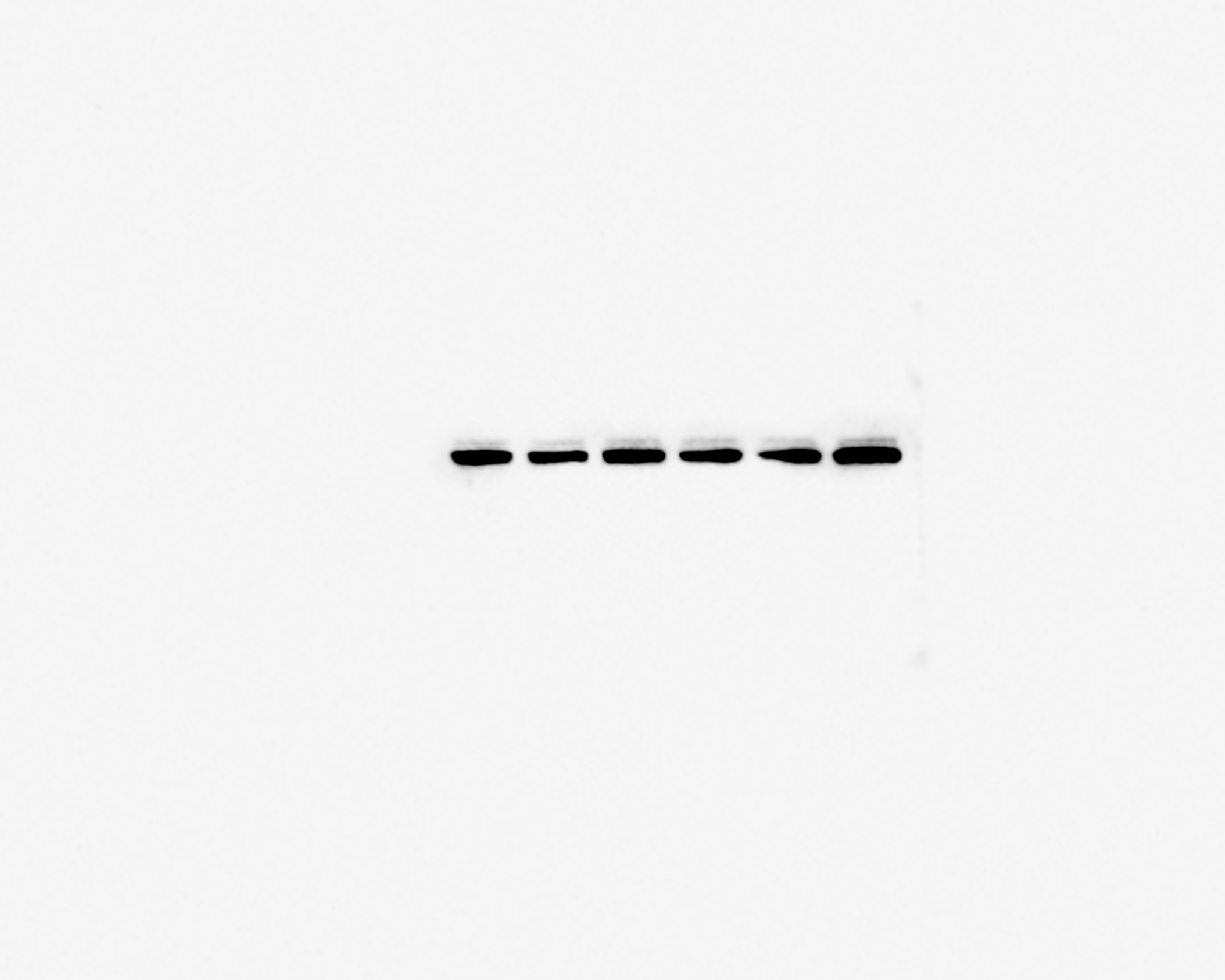

Supplement: Supplemental Information 3 [file peerj-12-18222-s003.zip › P65/pp65+p65 2_1(Chemiluminescence).tif]

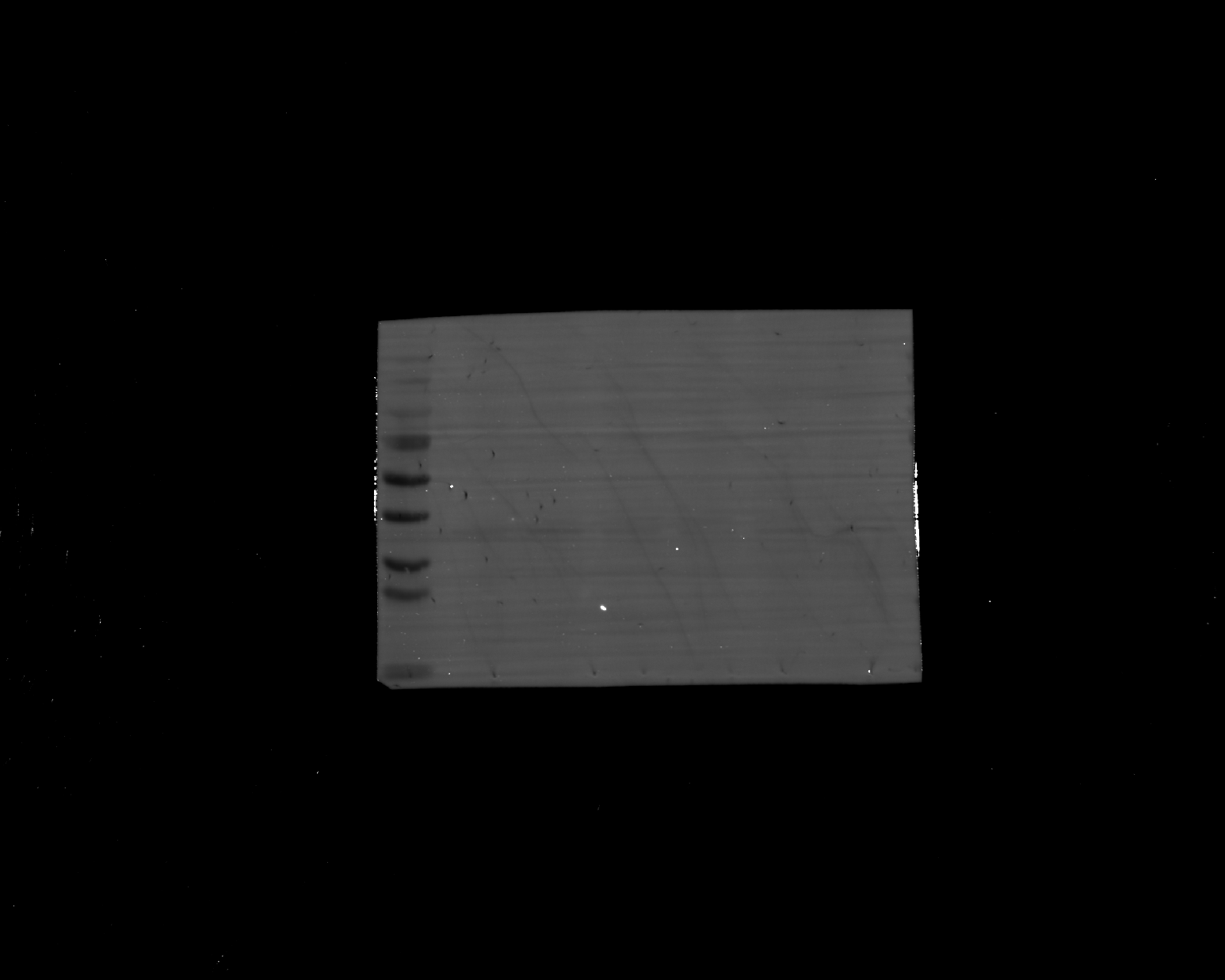

Supplement: Supplemental Information 3 [file peerj-12-18222-s003.zip › P65/pp65+p65 2_1(Colorimetric).tif]

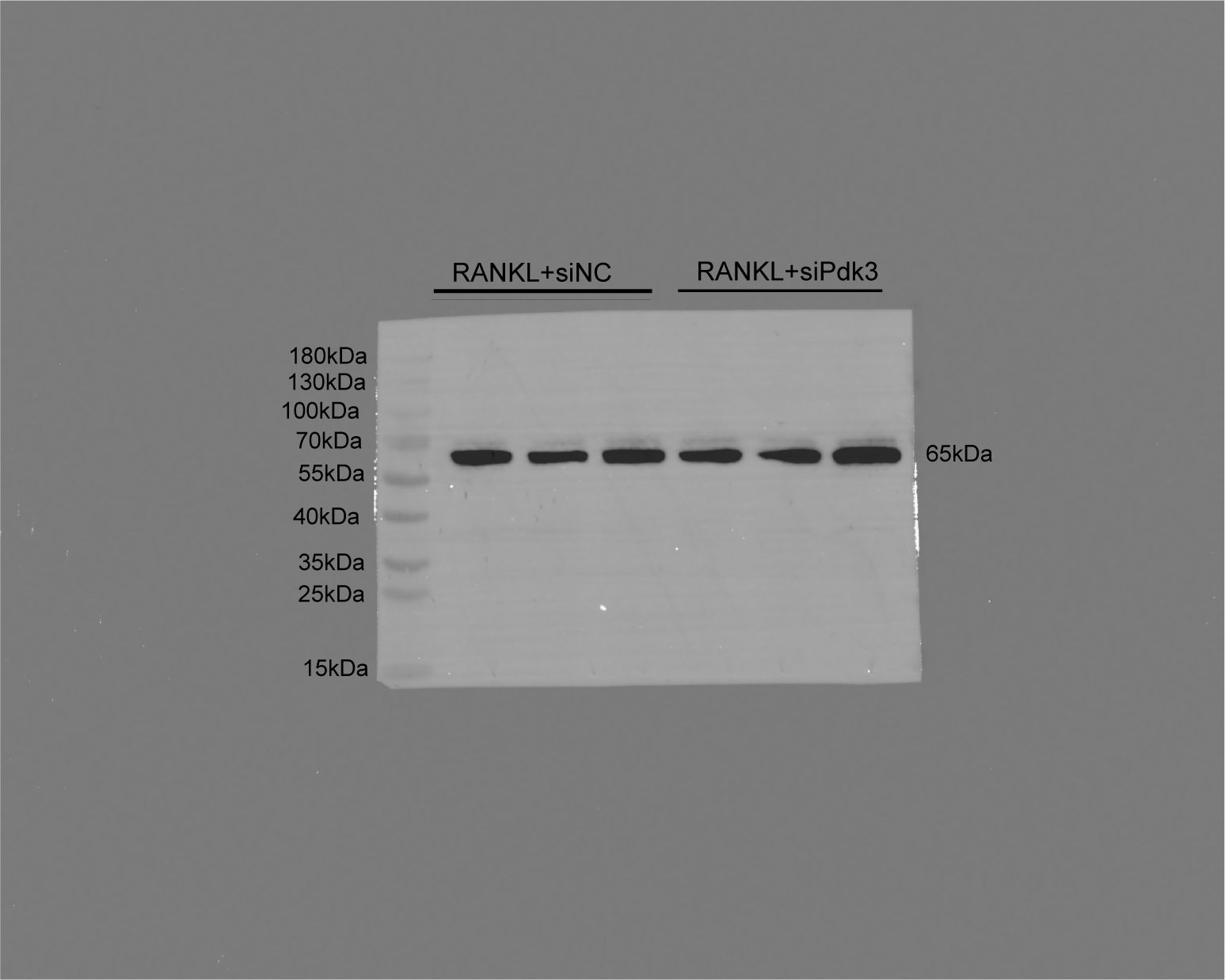

Supplement: Supplemental Information 3 [file peerj-12-18222-s003.zip › P65/pp65+p65 2_1(Composite)-01.tif]

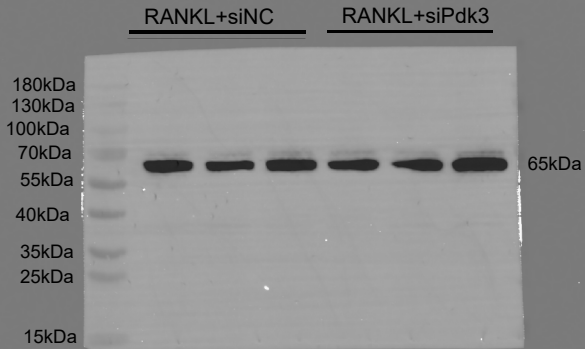

Supplement: Supplemental Information 3 [file peerj-12-18222-s003.zip › P65/pp65+p65 2_1(Composite).pdf]

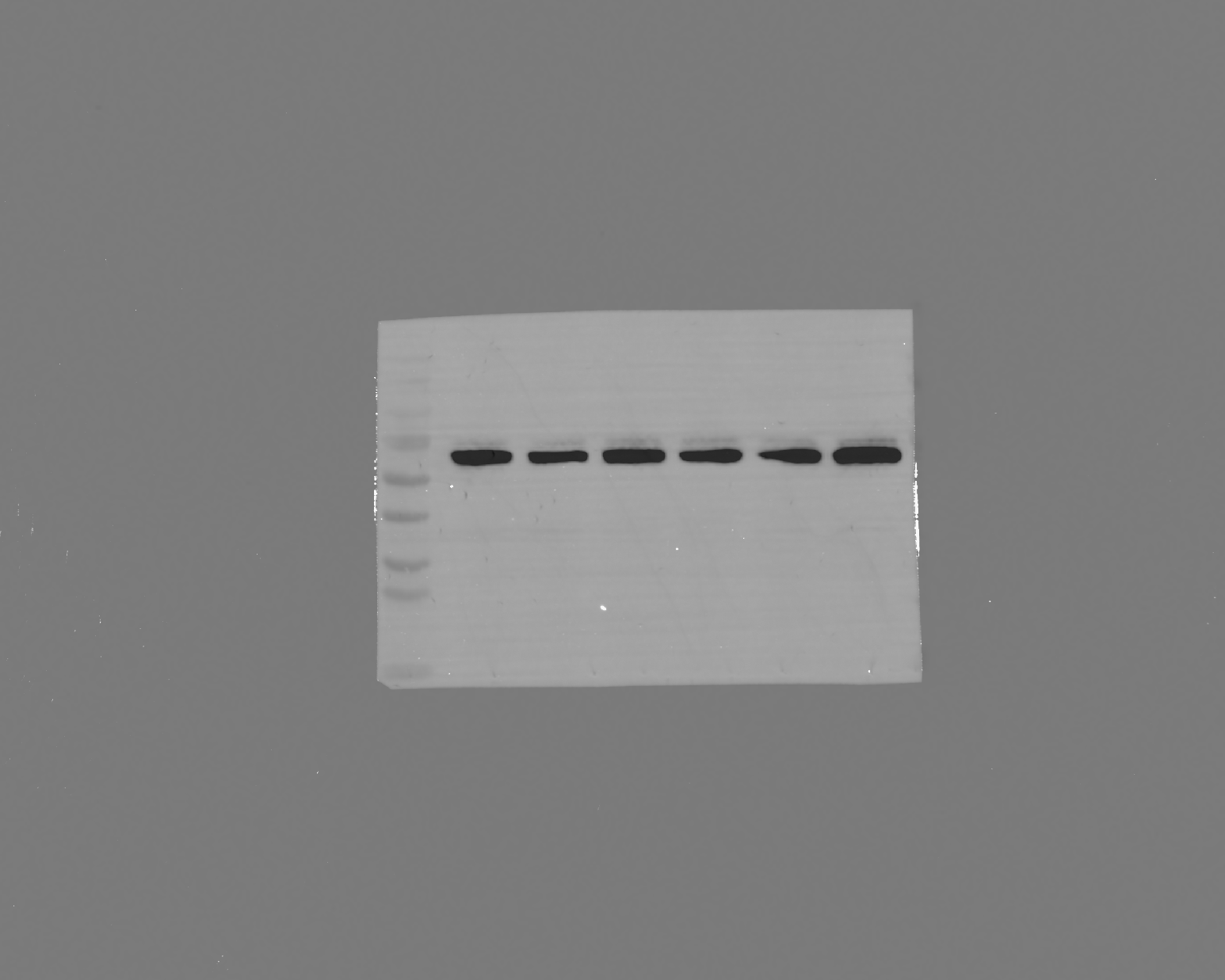

Supplement: Supplemental Information 3 [file peerj-12-18222-s003.zip › P65/pp65+p65 2_1(Composite).tif]

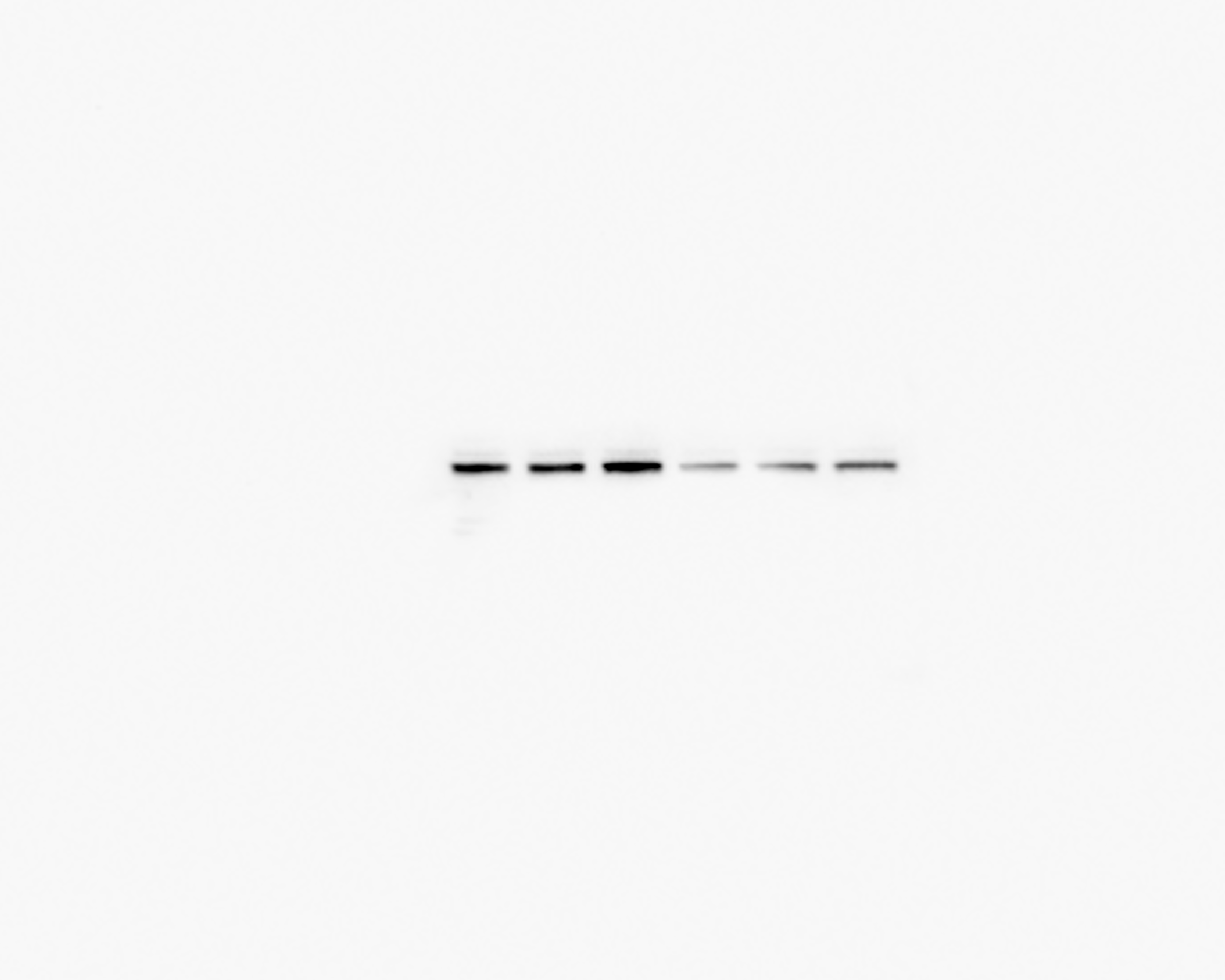

Supplement: Supplemental Information 3 [file peerj-12-18222-s003.zip › P65/pp65+p65 2_2(Chemiluminescence).tif]

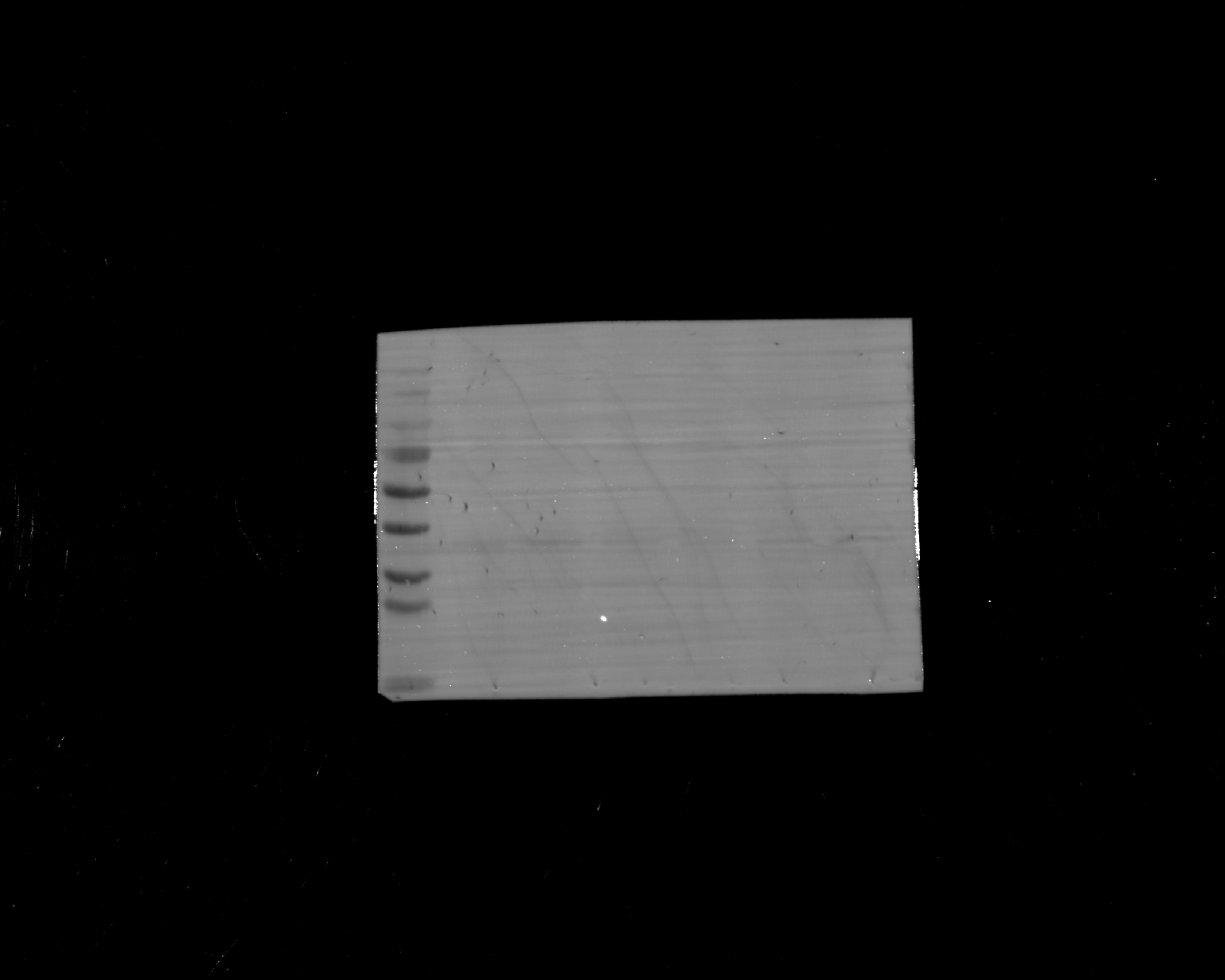

Supplement: Supplemental Information 3 [file peerj-12-18222-s003.zip › P65/pp65+p65 2_2(Colorimetric).tif]

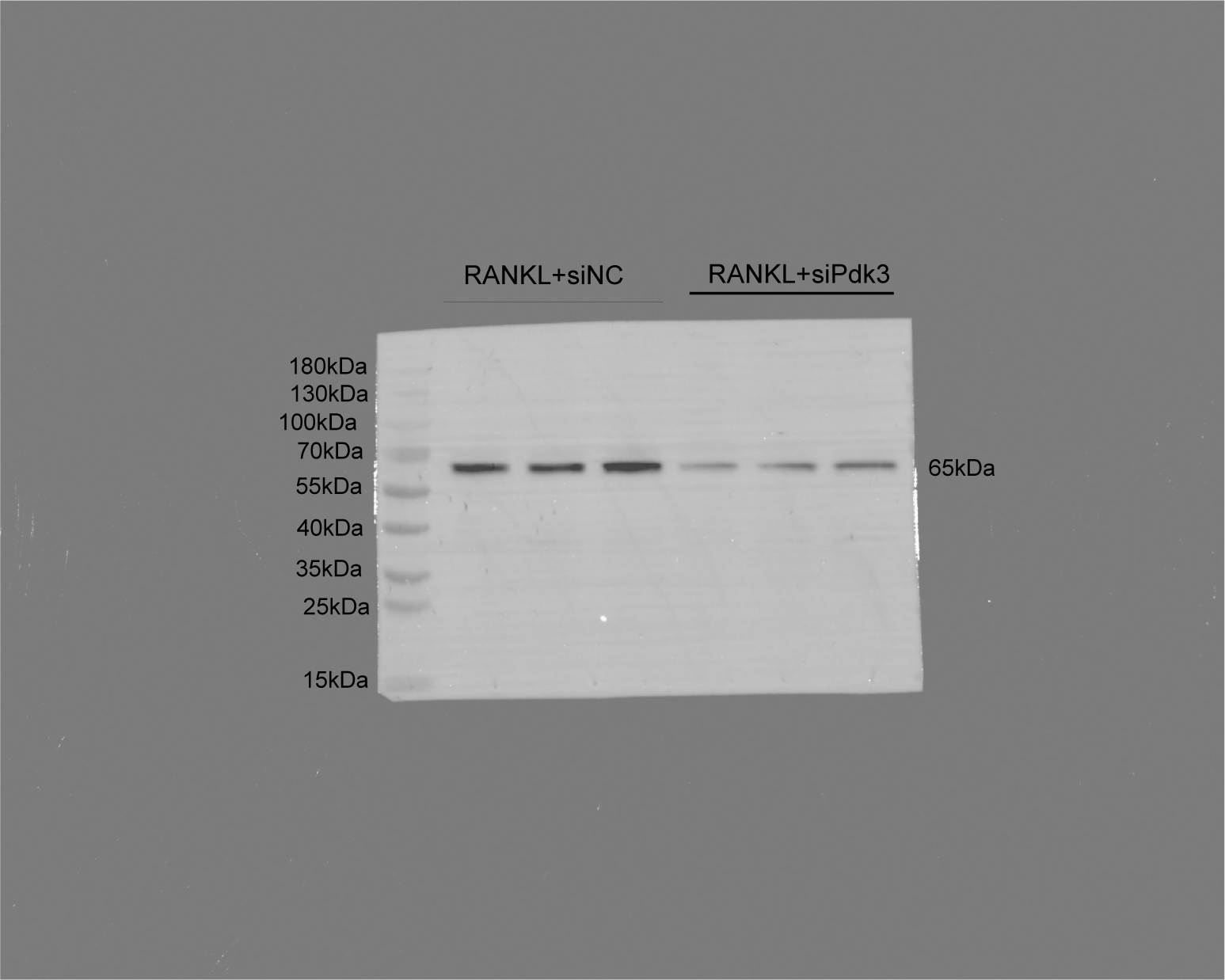

Supplement: Supplemental Information 3 [file peerj-12-18222-s003.zip › P65/pp65+p65 2_2(Composite)-01.tif]

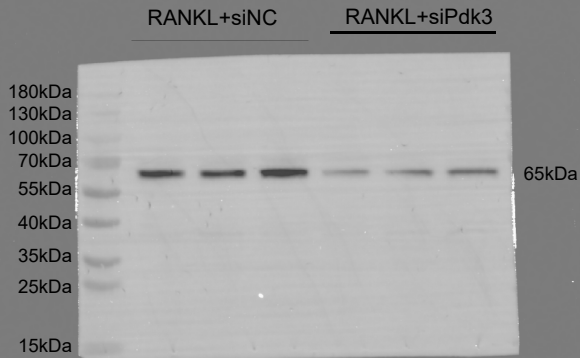

Supplement: Supplemental Information 3 [file peerj-12-18222-s003.zip › P65/pp65+p65 2_2(Composite).pdf]

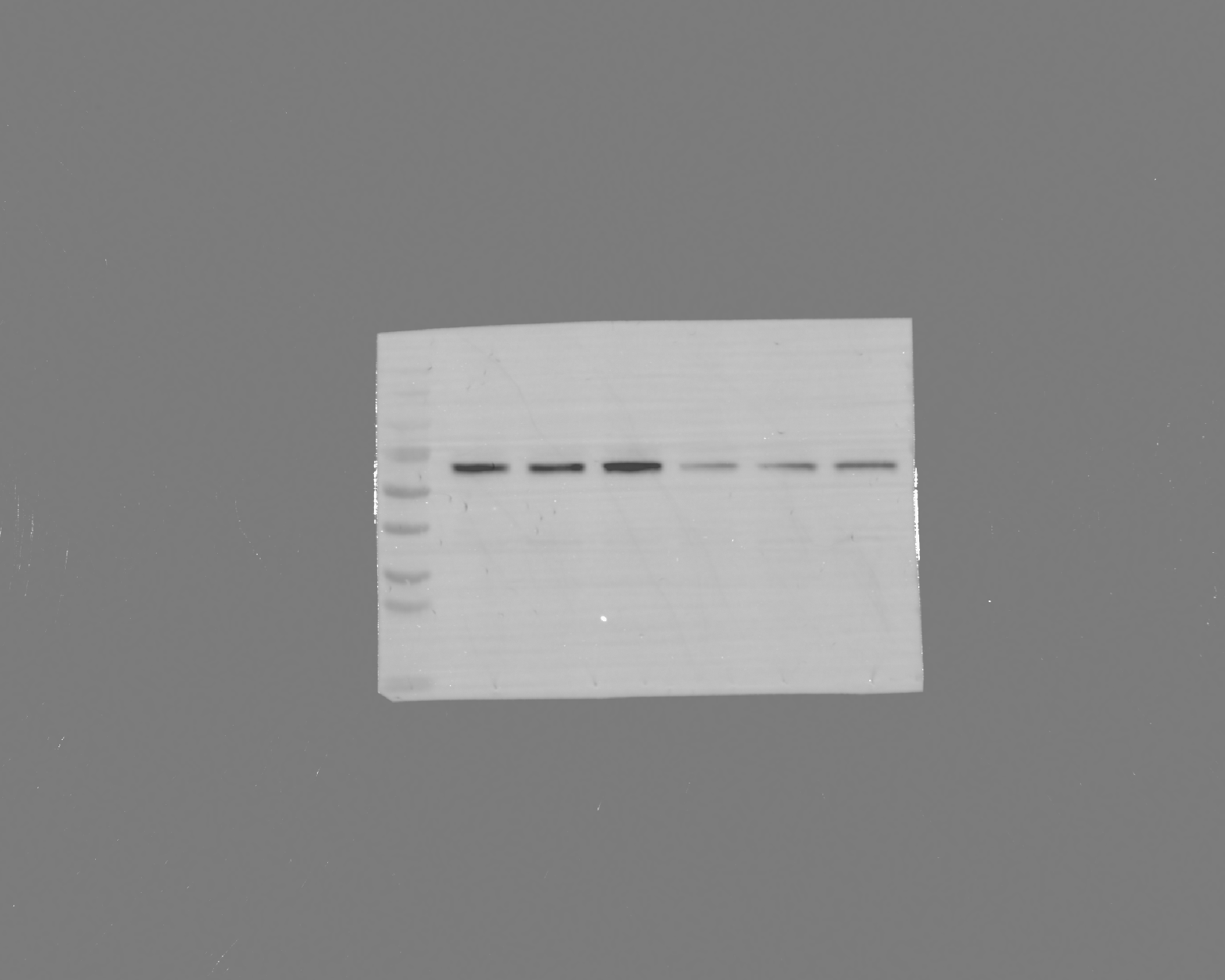

Supplement: Supplemental Information 3 [file peerj-12-18222-s003.zip › P65/pp65+p65 2_2(Composite).tif]

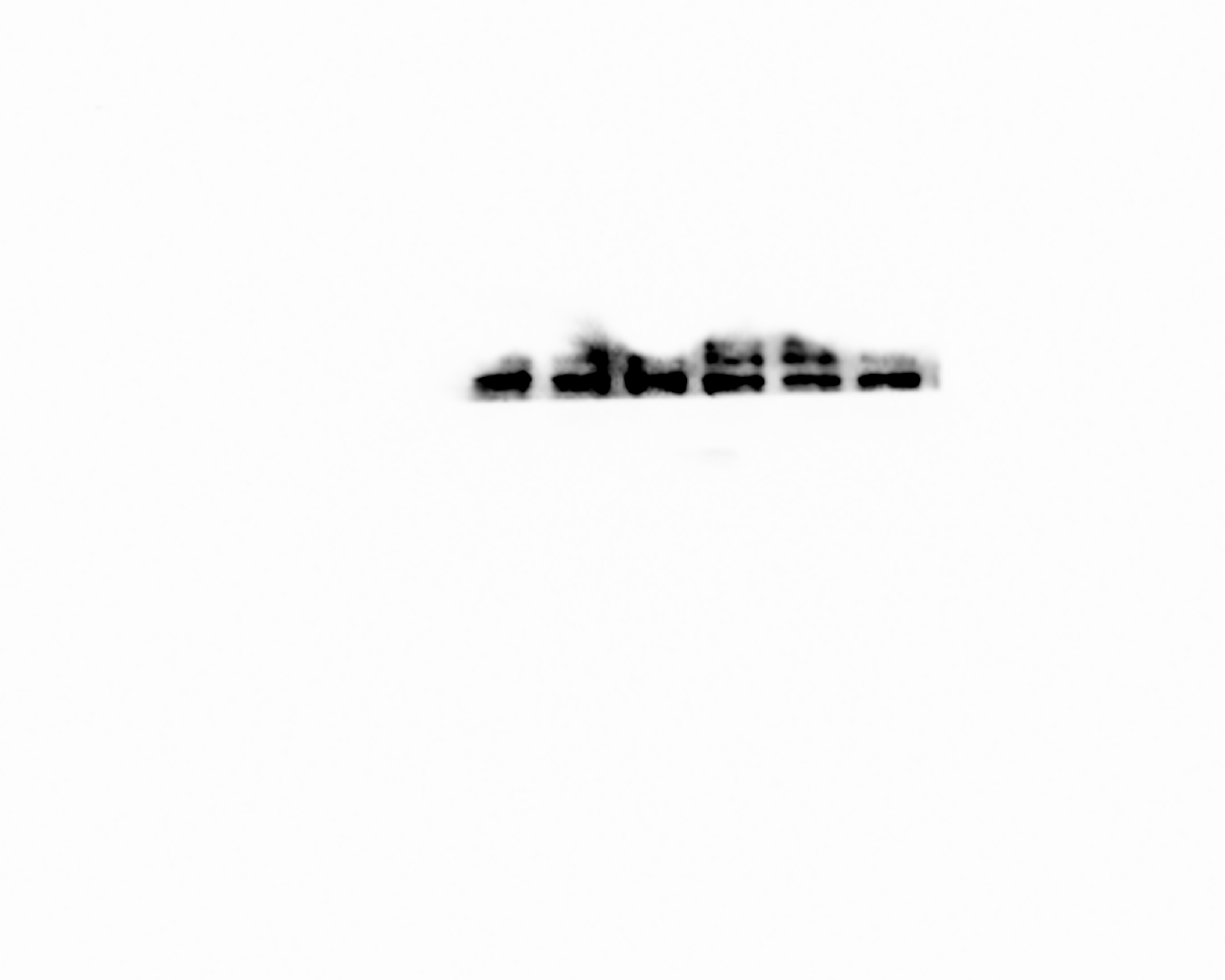

Supplement: Supplemental Information 3 [file peerj-12-18222-s003.zip › STAT1/6 pstat1+stat1 2_1(Chemiluminescence).tif]

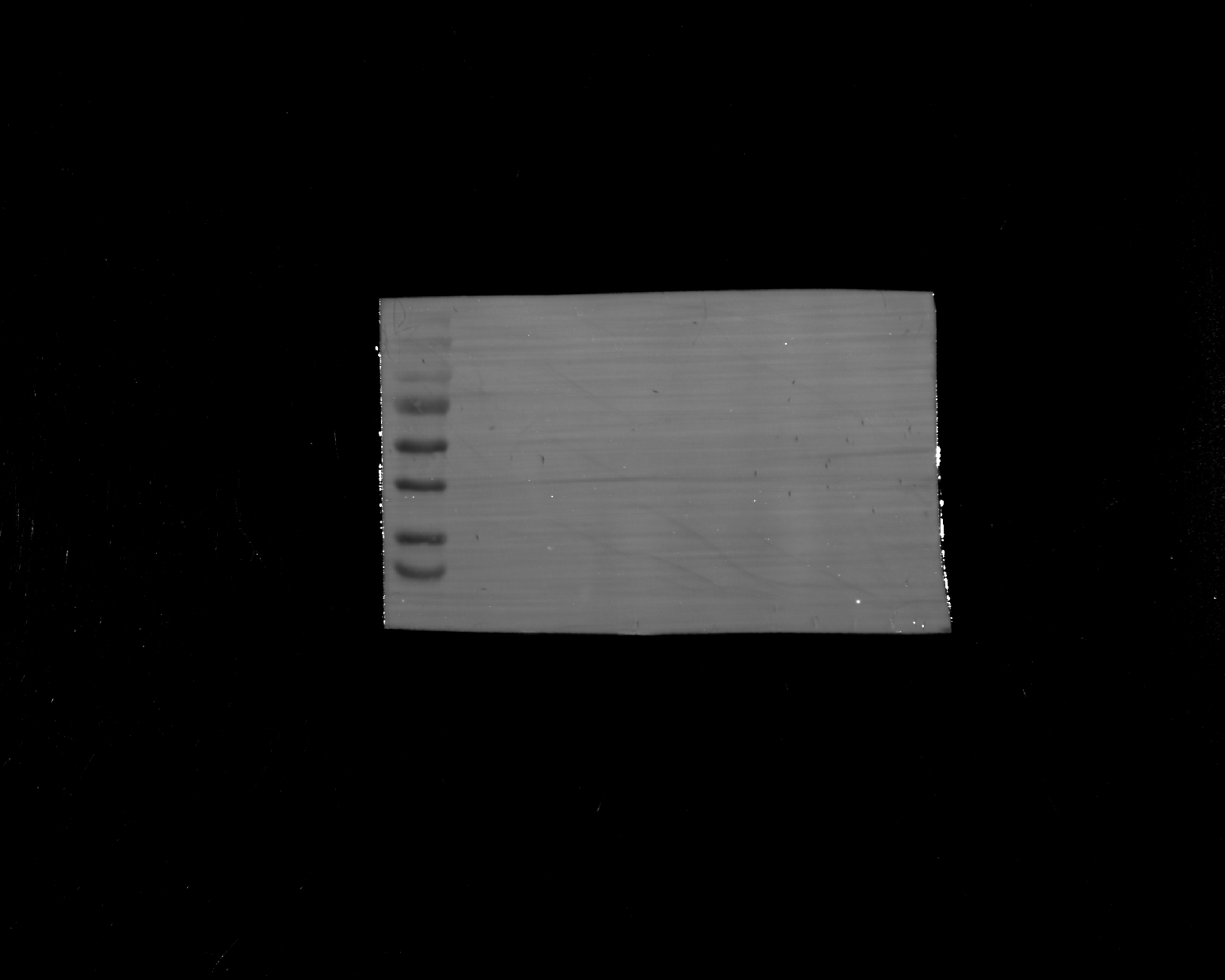

Supplement: Supplemental Information 3 [file peerj-12-18222-s003.zip › STAT1/6 pstat1+stat1 2_1(Colorimetric).tif]

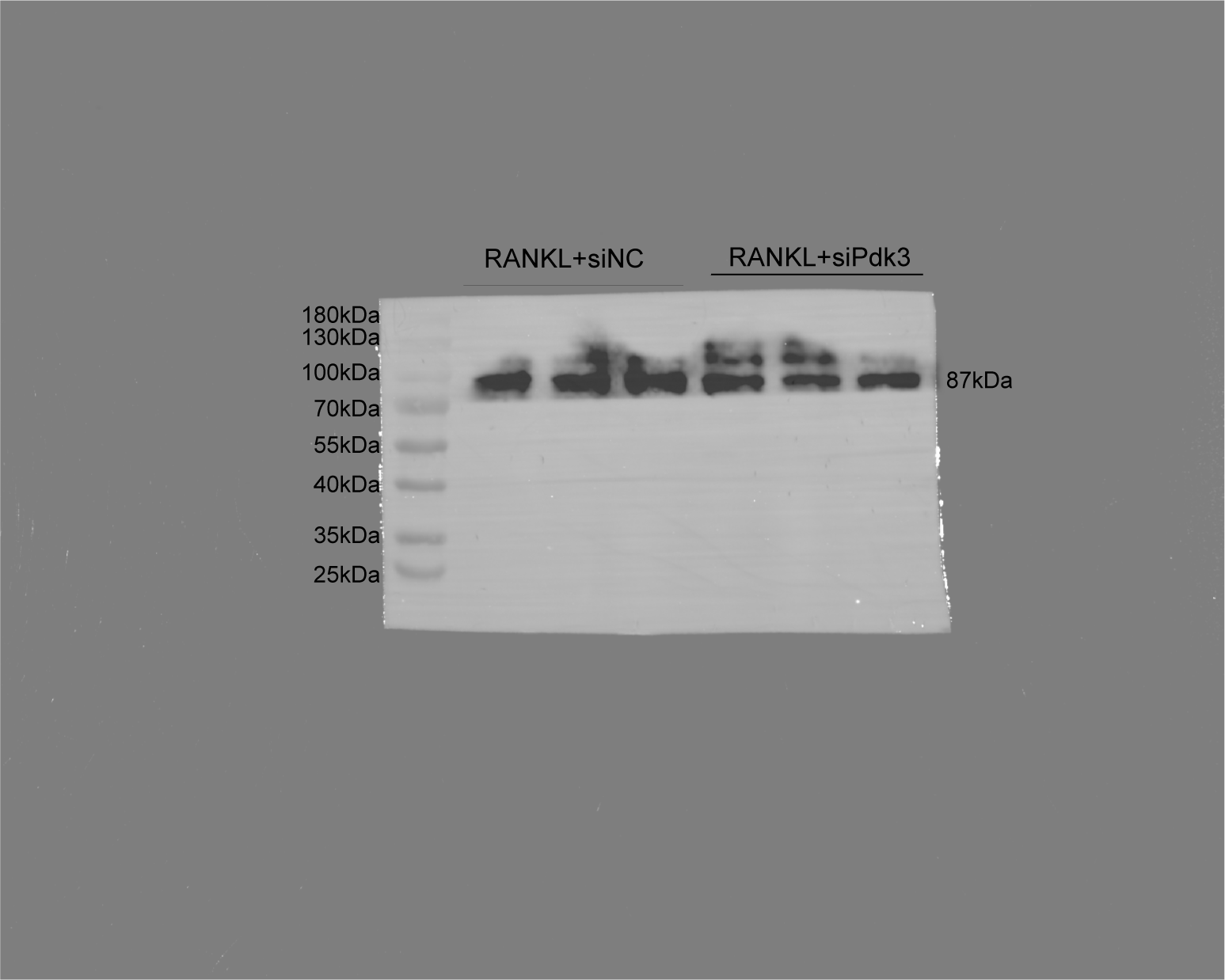

Supplement: Supplemental Information 3 [file peerj-12-18222-s003.zip › STAT1/6 pstat1+stat1 2_1(Composite)-01.tif]

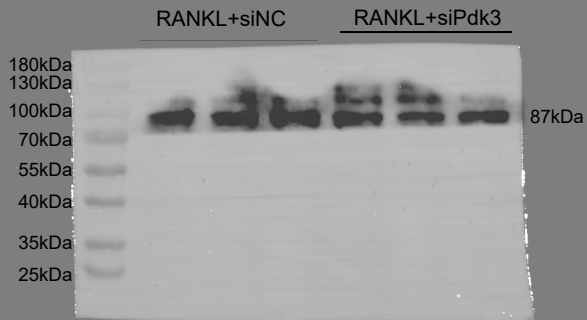

Supplement: Supplemental Information 3 [file peerj-12-18222-s003.zip › STAT1/6 pstat1+stat1 2_1(Composite).pdf]

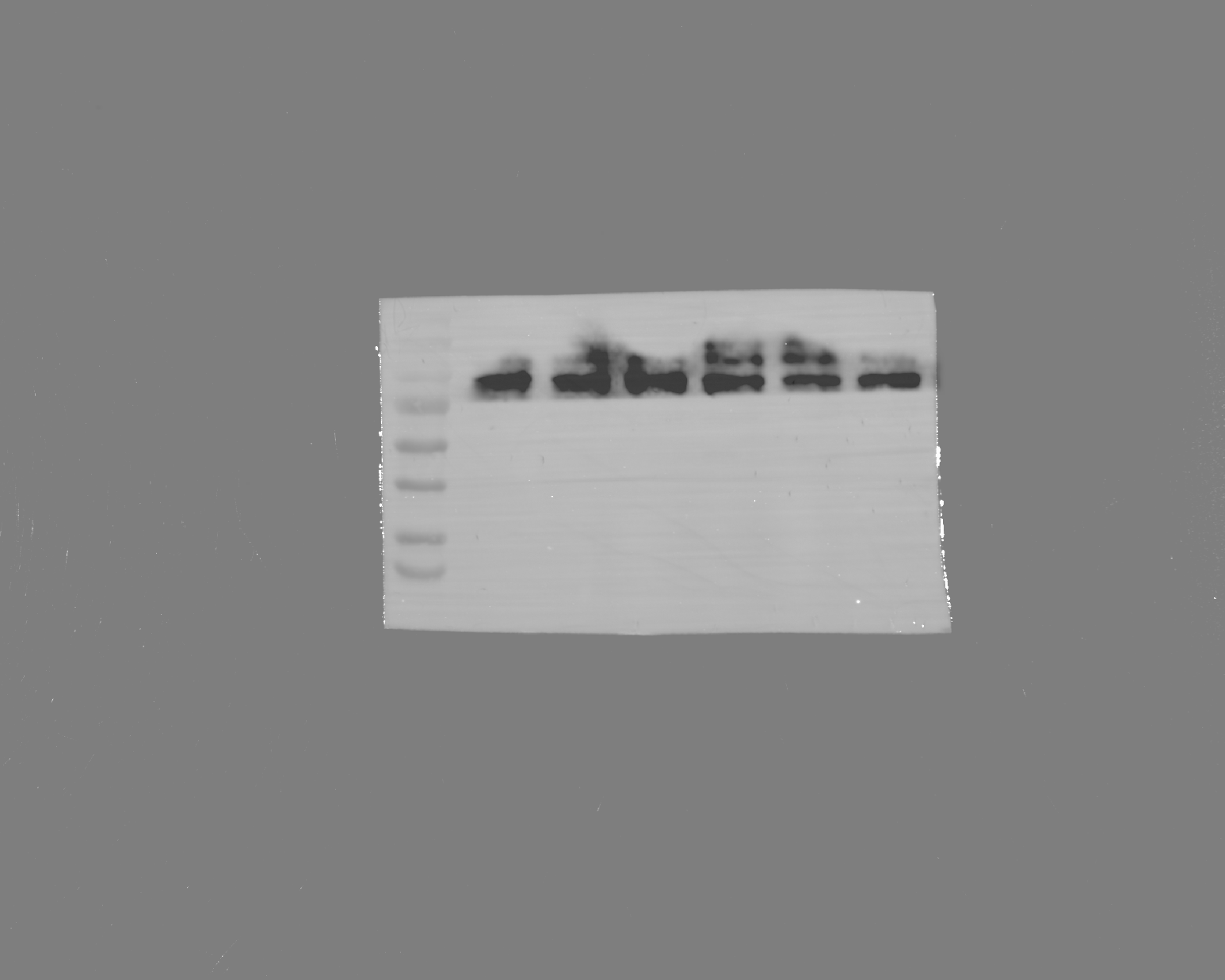

Supplement: Supplemental Information 3 [file peerj-12-18222-s003.zip › STAT1/6 pstat1+stat1 2_1(Composite).tif]

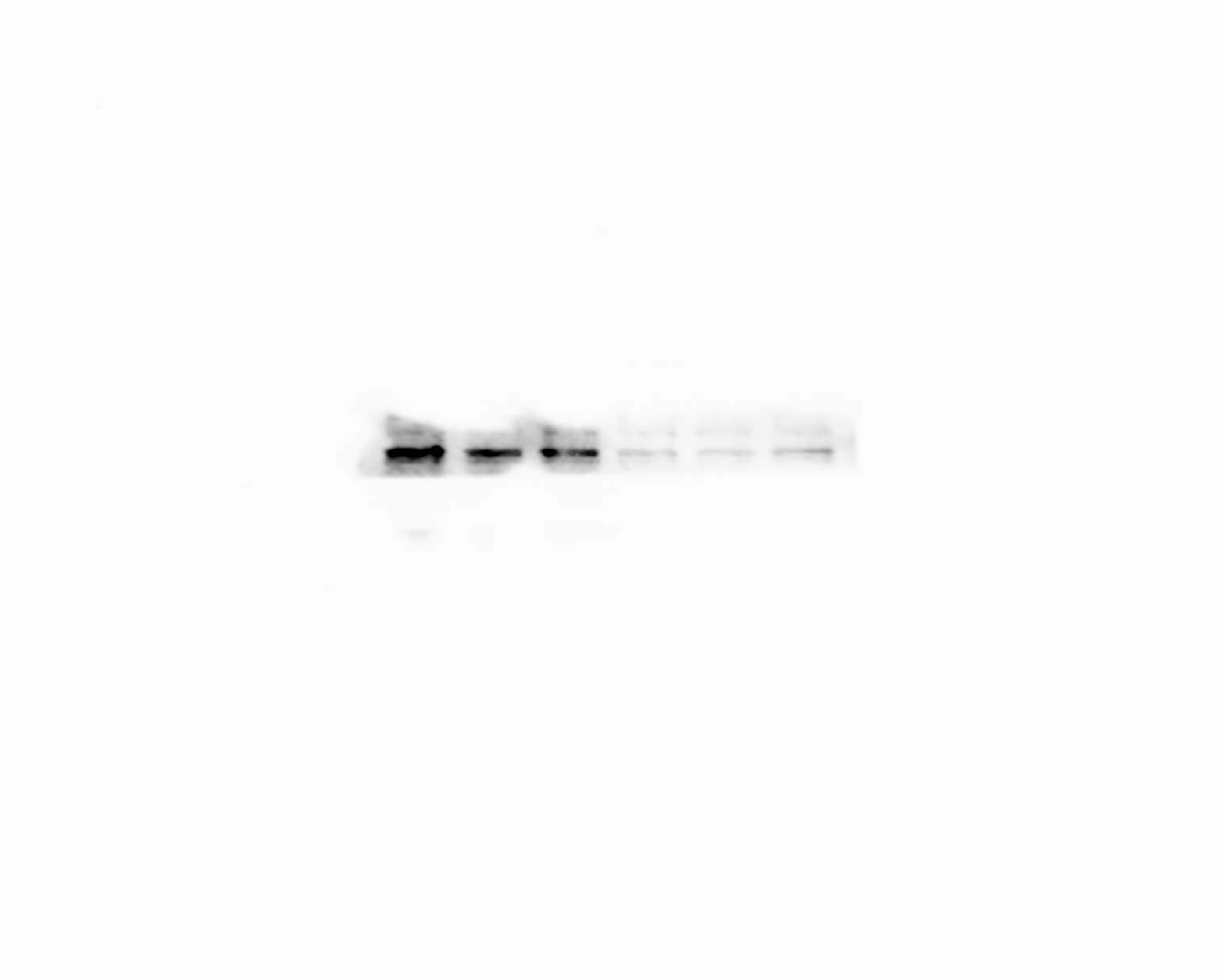

Supplement: Supplemental Information 3 [file peerj-12-18222-s003.zip › STAT1/6 pstat1+stat1 2_2(Chemiluminescence).tif]

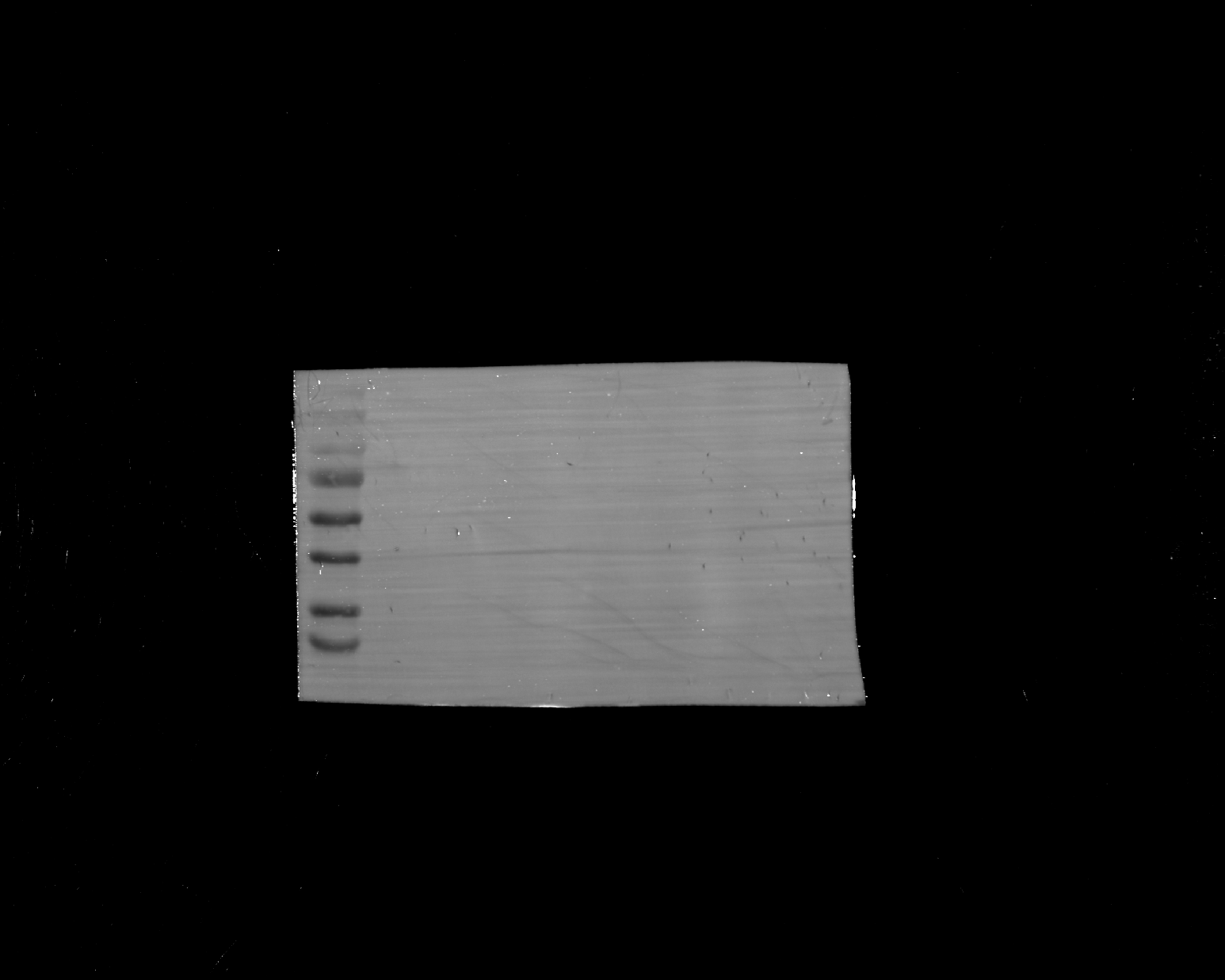

Supplement: Supplemental Information 3 [file peerj-12-18222-s003.zip › STAT1/6 pstat1+stat1 2_2(Colorimetric).tif]

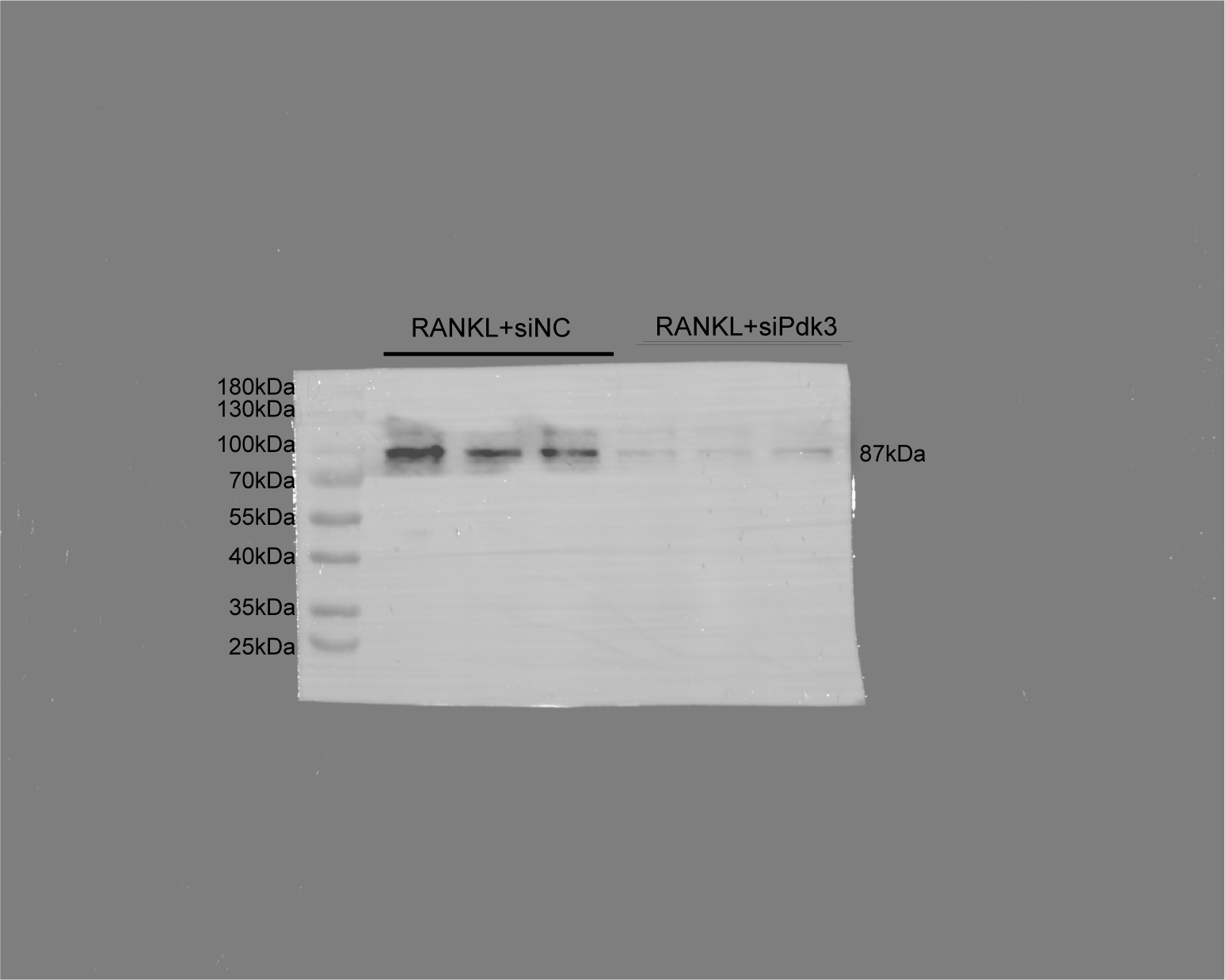

Supplement: Supplemental Information 3 [file peerj-12-18222-s003.zip › STAT1/6 pstat1+stat1 2_2(Composite)-01.tif]

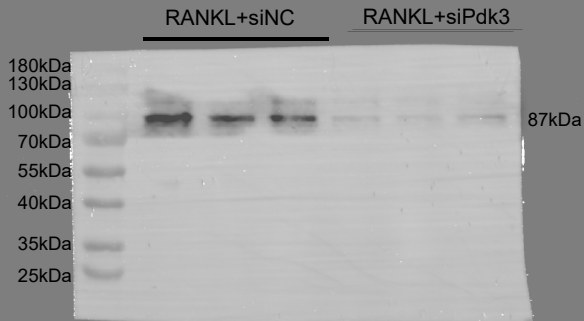

Supplement: Supplemental Information 3 [file peerj-12-18222-s003.zip › STAT1/6 pstat1+stat1 2_2(Composite).pdf]

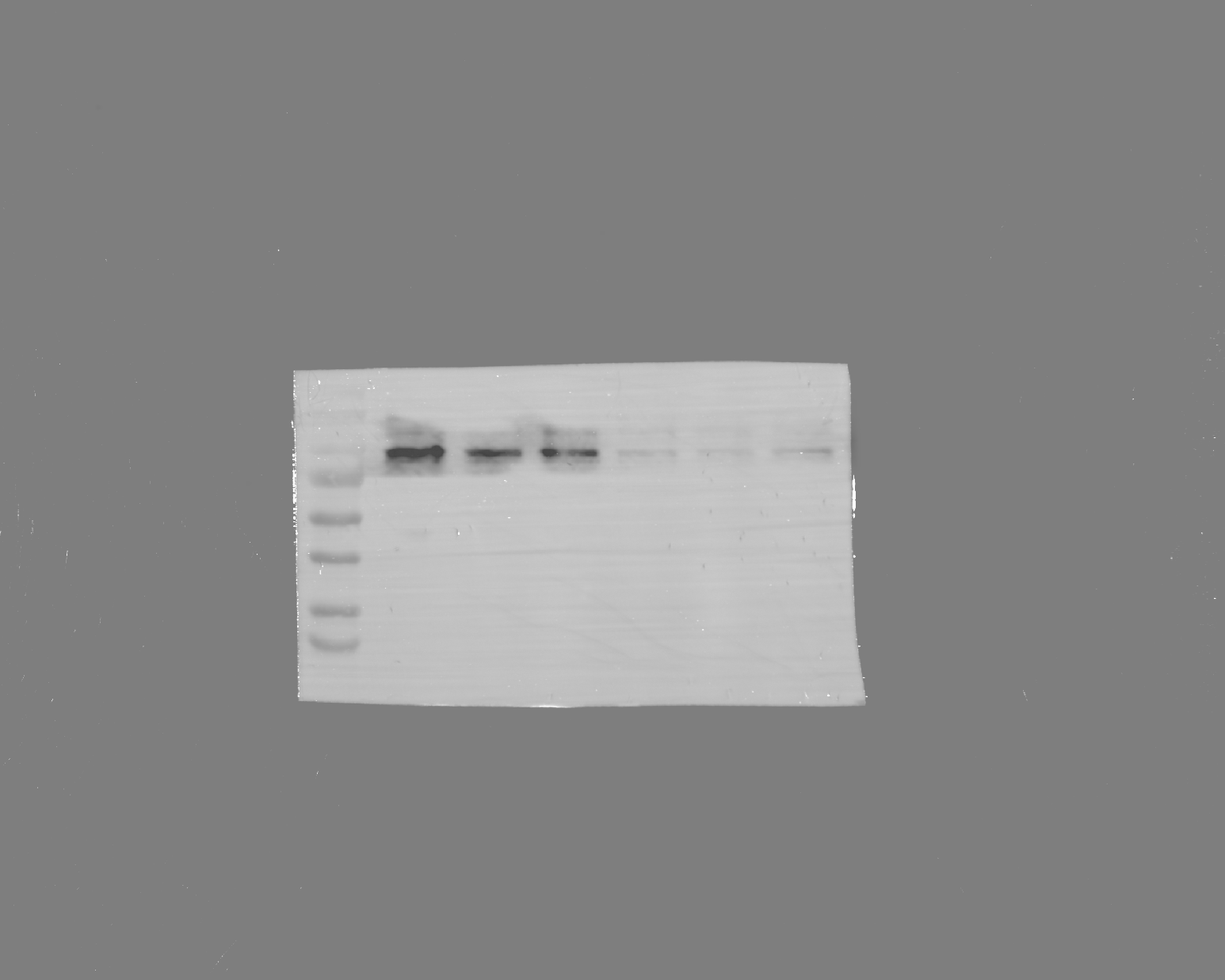

Supplement: Supplemental Information 3 [file peerj-12-18222-s003.zip › STAT1/6 pstat1+stat1 2_2(Composite).tif]
